# Global Transcriptomic Analysis of Bacteriophage-Host Interactions between a Kayvirus Therapeutic Phage and Staphylococcus aureus

**DOI:** 10.1128/spectrum.00123-22

**Published:** 2022-04-18

**Authors:** Adéla Finstrlová, Ivana Mašlaňová, Bob G. Blasdel Reuter, Jiří Doškař, Friedrich Götz, Roman Pantůček

**Affiliations:** a Department of Experimental Biology, Faculty of Science, Masaryk University, Brno, Czech Republic; b Vésale Bioscience, Vésale Pharma, Noville sur Mehaigne, Belgium; c Microbial Genetics, Interfaculty Institute of Microbiology and Infection Medicine Tübingen, University of Tübingen, Tübingen, Germany; Institut Pasteur

**Keywords:** phage-host interactions, *Staphylococcus* phages, Kayvirus, RNA-Seq, viral transcription, noncoding RNA, prophages, bacteriophage therapy, *Staphylococcus aureus*, transcriptome

## Abstract

Kayviruses are polyvalent broad host range staphylococcal phages with a potential to combat staphylococcal infections. However, the implementation of rational phage therapy in medicine requires a thorough understanding of the interactions between bacteriophages and pathogens at omics level. To evaluate the effect of a phage used in therapy on its host bacterium, we performed differential transcriptomic analysis by RNA-Seq from bacteriophage K of genus Kayvirus infecting two Staphylococcus aureus strains, prophage-less strain SH1000 and quadruple lysogenic strain Newman. The temporal transcriptional profile of phage K was comparable in both strains except for a few loci encoding hypothetical proteins. Stranded sequencing revealed transcription of phage noncoding RNAs that may play a role in the regulation of phage and host gene expression. The transcriptional response of S. aureus to phage K infection resembles a general stress response with differential expression of genes involved in a DNA damage response. The host transcriptional changes involved upregulation of nucleotide, amino acid and energy synthesis and transporter genes and downregulation of host transcription factors. The interaction of phage K with variable genetic elements of the host showed slight upregulation of gene expression of prophage integrases and antirepressors. The virulence genes involved in adhesion and immune evasion were only marginally affected, making phage K suitable for therapy.

**IMPORTANCE** Bacterium Staphylococcus aureus is a common human and veterinary pathogen that causes mild to life-threatening infections. As strains of S. aureus are becoming increasingly resistant to multiple antibiotics, the need to search for new therapeutics is urgent. A promising alternative to antibiotic treatment of staphylococcal infections is a phage therapy using lytic phages from the genus Kayvirus. Here, we present a comprehensive view on the phage-bacterium interactions on transcriptomic level that improves the knowledge of molecular mechanisms underlying the Kayvirus lytic action. The results will ensure safer usage of the phage therapeutics and may also serve as a basis for the development of new antibacterial strategies.

## INTRODUCTION

Lytic bacteriophages are natural viral predators of bacteria. They provide the basis for phage therapy, which is more and more applied in human and veterinary medicine in response to increasing antibiotic resistance. Basic phage research and its applications are intensively pursued worldwide (1). For the nontraditional treatment of infections caused by Staphylococcus aureus strains, which belongs to the ESKAPE pathogen group with increasing multidrug resistance (2), the most convenient phages for therapeutic application are lytic bacteriophages of the genus Kayvirus (3–6). These bacteriophages are effective therapeutic agents for the treatment of severe infections with multidrug-resistant staphylococci (7).

*In vitro* studies have demonstrated that kayviruses have an extremely broad host range and are capable of lysing up to 90% of S. aureus strains tested (8) along with numerous non-S. aureus staphylococcal species (9–11). Furthermore, the cultivation of phages with resistant bacteria and phage breeding has been shown to lead to the accessible selection of new phage variants with a widened host range (8, 12, 13). The genomic characterization of kayviruses shows that they do not carry bacterial virulence or antibiotic resistance genes, and that they lack the capacity to integrate into the bacterial genome or facilitate horizontal gene transfer (14). However, it still remains to be answered how the lytic phages interact with S. aureus pathogenicity islands (SaPIs) and prophages, which drive horizontal exchange of accessory genes associated with virulence and antimicrobial resistance (15).

To fully demonstrate the safety of the use of kayviruses in human and veterinary medicine, it is necessary to elucidate their relationship with their hosts with multi-omics approaches. In this study, we used phage K, the type species of the Kayvirus genus, frequently used as a model staphylococcal myovirus. The phage K genome is 148 kb long consisting of dsDNA with long terminal repeats (16) and exhibits more than 95% nucleotide identity to other Kayvirus strains, which are used as phage therapeutics. Structurally, kayviruses have a classical myovirus morphology with contractile tail and double-layered baseplate (17). We characterized the transcriptional regulation of phage K during the infection of two S. aureus strains with distinct prophage content. The RNA-Seq data revealed how the phage transcription proceeds and how it is controlled. By comparing the transcription of phage-infected with uninfected bacteria, we show the phage’s impact in terms of transcriptional changes in metabolic pathways and the expression of genes that play an important role in staphylococcal pathogenesis.

## RESULTS

### Model organisms and experimental design.

The transcriptomic progression of Staphylococcus phage K infected cells was studied by differential expression analysis in S. aureus strains Newman and SH1000 from clonal complex CC8, which differ in the regulators and prophage content (Table S1). Phage K efficiently propagates on both bacterial strains in liquid medium with similar growth characteristics. The phage K adsorption to SH1000 showed that 95% of the phage particles adsorbed in the first 2 min after phage addition, rising to 99% in 5 min, whereas adsorption to Newman showed that 87% of phage K virions in the first 2 min, rising to 97% in 5 min. The latent period was 30 min in SH1000 and 35 min in Newman, and the relative burst size was 27 ± 5 PFU in SH1000, which was higher than the 12 ± 5 PFU in Newman. Hence, sampling times of 2, 5, 10, 20, and 30 min were used for the stranded RNA sequencing performed in biological triplicates. The multiplicity of infection (MOI = 7) was chosen based on the depletion of CFU (CFU/mL) by at least 99.7% after the first 15 min of phage exposure. This demonstrates the synchronicity of infection and the validity of time points as relevant to a substantial majority of cells. Phage mRNA gradually replaced bacterial mRNA inside the cell, starting from 1% of phage reads at 2 min and increasing to 65% ± 7% at 30 min (Fig. S1).

### Phage K transcription.

The RNA-Seq data confirmed transcription from all 233 previously annotated CDS and 4 tRNAs of the phage K genome (18). The only untranscribed locus was the 450-bp-long region between genes gp191 and gp190, which had the lowest GC content in the whole phage genome (21.25%), strongly suggesting it as the location of the phage K origin of replication, which was further supported by the identification of two direct repeats serving as a putative binding place for Rep protein (19).

**FIG 1:**
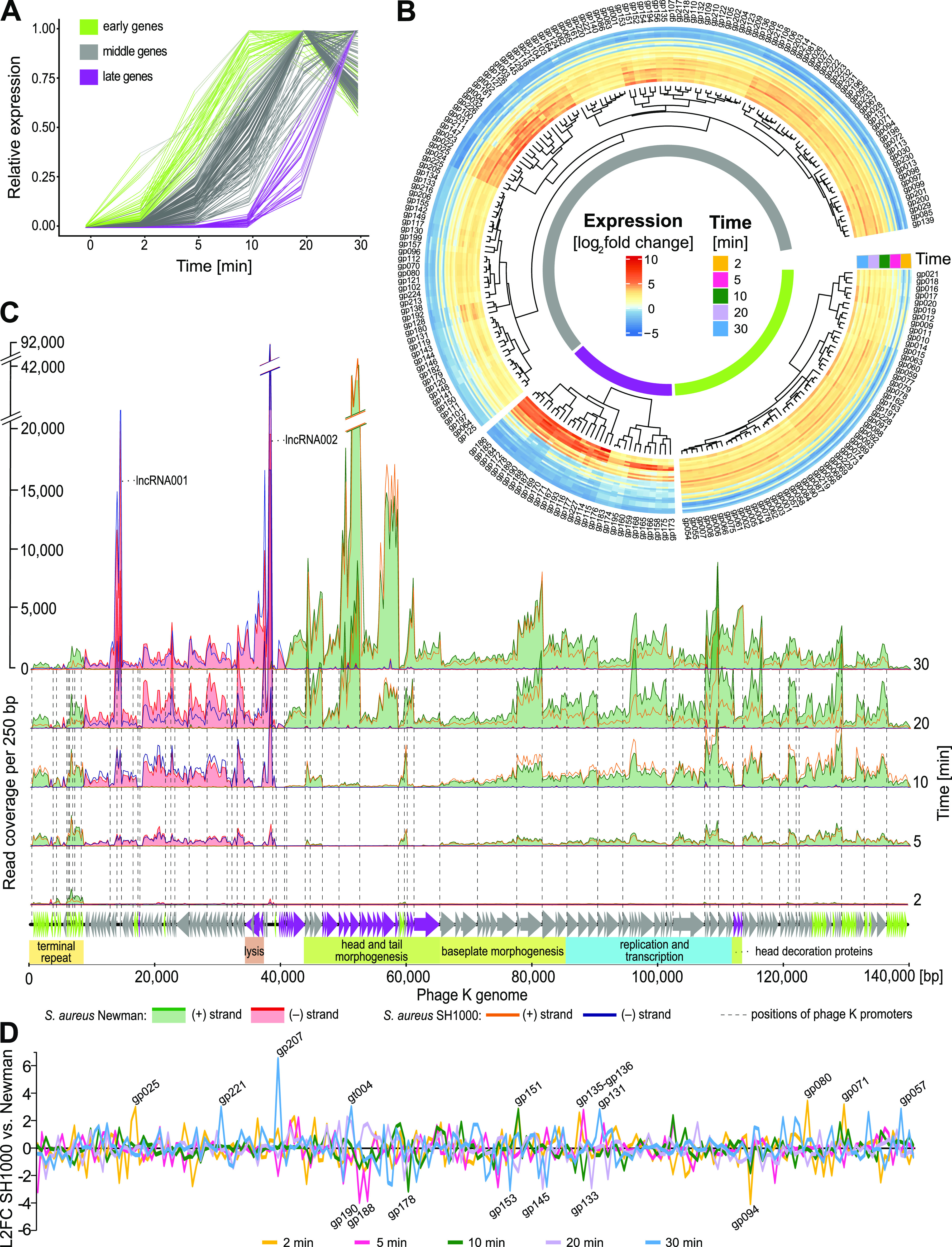
Phage K transcription analysis. (A) Normalized phage gene expression profile. Each line represents a gene. (B) Heatmap of phage log_2_-transformed normalized counts per gene generated using DESeq2. Clustering of phage genes by their expression divides the genes into three groups – early, middle, and late. (C) Transcriptional profile of phage K genome from plus and minus DNA strand in two S. aureus strains, Newman and SH1000. The genetic map of the phage genome is indicated by arrows showing the direction of transcription and the genome modules with known functions are depicted with color boxes bellow the ORF map. The depicted long terminal repeat (LTR) has aligned reads from both phage LTRs. The genes in panels A, B, and C are colored based on their transcription phase in the same way as in section (A). (D) Comparison of phage K gene expression in S. aureus SH1000 versus Newman. Each line represents relative expression in the analyzed sampling time points. Gene product numbers (gp) were obtained from previously published phage K genome (18).

The time periods selected between withdrawal of samples of infected cells for transcriptomic analysis (0, 2, 5, 10, 20, and 30 min) enable to divide phage genes into three distinct temporal transcript phases – early, middle, and late based on the time they reach maximum expression (Fig. 1A). The phage expression pattern complements the study of transcription of another Kayvirus vB_SauM-515A1, where only two transcriptional phases were reported (20). The heatmap constructed after DESeq2 normalization (21) of feature summarized reads also clusters the transcribed gene features into three major groups and several subgroups, indicating more structured expression control by phage regulators (Fig. 1B). The transcription is initiated from 83 putative promoters (Table S2A) by the host transcriptional machinery, because kayviruses do not encode their own RNA polymerase. The early and middle promoters were highly conserved (Fig. S2B), resembling host σ^70^ promoters, but late promoters lack the usual −35 sigma factor sequence motif, only retaining the conserved −10 motif (Fig. S2B). A marked end of transcription was found at 43 previously annotated terminators (18) as well as at additional 8 regions (Fig. S2A; Table S2B). The phage K transcriptional profile was comparable in both infected strains, but some expression differences were observed for different sampling times (Fig. 1C). The most differentially expressed (DE) genes between the strains were gp207 with higher expression in strain SH1000 and gp094 with higher expression in strain Newman both encoding hypothetical proteins (Fig. 1D).

Transcription of the phage K genes starts immediately after the infection of bacterial cells, as is evident from the detection of five abundant transcripts gp014-gp018 from left and right long terminal repeats (L- and R-LTR) at 2 min. The early genes reach their relative expression maximum within 10 min (Fig. 1C) and this group comprises 74 genes, which code for hypothetical proteins (Table S3). Only three genes are similar to genes in databases that code for protein domains of known function (Table S3). The middle genes represent the largest group of 132 genes, and reached their maximum relative transcript abundance within 20 min postinfection. The known middle genes are involved in transcription regulation, DNA metabolism and replication, and host DNA degradation (Table S3), such as gp097 sliding clamp inhibitor *sci*, an analogue of phage G1 gene gp240, which blocks host DNA replication (22). The four tRNAs are transcribed during the middle phase and are thus available for middle and late gene translation. The phage virion genes encoding baseplate components, putative hydrolase gp156, tail tube initiator gp154, putative baseplate components gp153 and gp145, tail sheath initiator gp152, and baseplate wedge protein gp151 are first to be expressed, followed by expression of genes for baseplate hub gp157, tail central spike gp155, baseplate arm proteins gp150 and gp149, tripod protein gp148, and receptor binding proteins gp146 and gp144. The late genes with 31 coding sequences have relatively low expression until 10 min, and they reached their expression maximum within 30 min and they comprise structural genes from the morphogenesis module, including large terminase gene gp183, portal protein gp176, prohead protease gp175, major capsid protein gp173, connector related gp172-gp167, tail sheath protein gp166, tail tube protein gp165, and tail tape measure protein gp158. Structural genes outside the morphogenesis module encoding head decoration proteins gp116-gp114 are also expressed in the late phase. The last genes that are expressed are holin gp193 and endolysin gp195 from the lysis module (Fig. 1C).

### Novel RNA species in phage K transcriptome.

Small and long noncoding RNAs (sncRNAs and lncRNAs) were detected in the bacteriophage transcription profile (Table 1). The highest number of reads aligned to the minus DNA strand in the intergenic region between gp191 and gt004 revealed the presence of lnc001, which is transcribed from its own early promoter (Fig. S2A; Table S2A). Lnc002 is located between gene gt001 and gp032 and transcribed from the promoter upstream of gt001 (Fig. S2A). Three putative sncRNAs were identified in the terminal repeat region, and four sncRNAs were scattered throughout the phage K genome. Possible targets of the sncRNAs in phage K and S. aureus Newman genome are listed in Table 1. Because most of the identified bacterial target genes were not differentially expressed on a transcriptional level by 30 min in infection, the sncRNAs possibly have a regulatory function impacting expression through differential translation.

**TABLE 1:** Noncoding RNA species in phage K genome^*a*^

Name	Genome position	Strand	Location with reference to other genes	Possible targets phage K	Possible targets S. aureus Newman
lnc001	13595-14229	Minus	Intergenic region gt001 and gp032	n.a.	n.a.
lnc002	37679-38832	Minus	Intergenic region gp191 and gt004	n.a.	n.a.
snc001	3114-3628	Plus	Antisense gp009	41488-41509 between genes gp184 and gp185	NWMN_RS07005 threonine synthase, NWMN_RS02270 hypothetical protein, NWMN_RS01465 hypothetical protein
snc002	5022-5067	Minus	Intergenic region gp012 and gp013	n.a.	NWMN_RS13395 hypothetical protein NWMN_RS00660 acyl-CoA dehydrogenase NWMN_RS09245 polysaccharide biosynthesis protein
snc003	6186-6352	Plus	Intergenic region gp015 and gp016	39055-39071 plus strand upstream of 5′ end gp191	NWMN_RS15600 hypothetical protein NWMN_RS07800 30S ribosomal protein S1 NWMN_RS11190 hypothetical protein
snc004	8370-8665	Minus	Intergenic region gp021 and gp022	n.a	NWMN_RS01765 NWMN_RS10130 NWMN_RS05865 FibU/BppU baseplate protein
snc005	40560-40861	Plus	Antisense to 5‘ end of gp186	9061-9122 overlap of genes gp023 and gp024	NWMN_RS06190 phospho-N-acetylmuramoyl-pentapeptide- transferase NWMN_RS07235 tryptophan synthase subunit beta NWMN_RS14215 NmrA/HSCARG family protein
snc006	119140-119325	Plus	Intergenic region gp104 and gp105	35440-35476 in intron gp194	NWMN_RS14275 fructosamine kinase family protein NWMN_RS03730 hypothetical protein NWMN_RS14870 DNA-binding protein
snc007	127601-127826	Plus	Intergenic region gp084 and gp085	15355-15429 upstream of stop codon gp231	NWMN_RS07550 virulence factor C NWMN_RS05190 quinol oxidase subunit 4 NWMN_RS02165 type II toxin-antitoxin system PemK/MazF family toxin NWMN_RS07435 nitric oxide reductase activation protein NorD

a For small ncRNA, one possible RNA target in the phage K genome (GenBank accession number NC_005880.2), and the three RNA targets with the highest interaction energy in the S. aureus Newman genome are listed. n.a., not available, no target was found.

### Transcriptional response of the host.

The two S. aureus strains, SH1000 and Newman, share 33 downregulated and 75 upregulated genes in response to phage K infection. The complete list of DE genes with log_2_ fold changes (L2FC) ± 0.58 with *P* < 0.05 corresponding to 1.5-fold upregulation or downregulation is given in Table S4 and selected annotated genes are listed in Table 2. The time expression profile was analyzed in a set of genes from strain Newman with L2FC ± 1.5 (Fig. S3).

**TABLE 2:** Differentially expressed bacterial genes: selected annotated genes of two S. aureus strains, Newman and SH1000, that are significantly up- or downregulated during phage K infection compared to uninfected control based on RNA-Seq data^*a*^

		Fold change			
Gene		Newman		SH1000	
(RefSeq gene ID)	Function, protein	10 min	20 min	10 min	20 min
Regulators				
NWMN_RS11225	Accessory gene regulator AgrA	n.s.	0.51	n.s.	n.s.
NWMN_RS11220	Accessory gene regulator AgrC	n.s.	0.65	n.s.	n.s.
NWMN_RS11395	RNA polymerase sigma factor SigB	1.94	1.58	1.65	n.s.
NWMN_RS07470	Response regulator transcription factor ArlR	2.18	n.s.	1.99	n.s.
NWMN_RS07465	Sensor histidine kinase ArlS	1.95	n.s.	2.11	n.s.
NWMN_RS08890	Sensor protein kinase WalK	1.97	n.s.	1.57	n.s.
NWMN_RS03715	Transcriptional regulator MgrA	0.46	0.48	n.s.	n.s.
Stress response				
NWMN_RS14920	Cold-shock protein CspG	0.14	0.14	0.18	0.28
NWMN_RS04305	Cold-shock protein CspC	0.20	0.19	0.21	0.31
NWMN_RS13180	Oxygen regulatory protein NreC	5.28	n.s.	n.s.	n.s.
NWMN_RS13185	Oxygen sensor histidine kinase NreB	3.23	3.00	n.s.	n.s.
NWMN_RS05970	Thioredoxin TrxA	n.s	0.59	n.s.	n.s.
NWMN_RS03810	Glycosyltransferase CsbB	3.15	n.s.	n.s.	n.s.
NWMN_RS07065	SOS-response protein LexA	0.48	n.s.	0.58	n.s.
NWMN_RS03125	Uracil-DNA glycosylase Ung	0.62	n.s.	n.s.	n.s.
Pathogenesis				
NWMN_RS06080	Alpha hemolysin Hly	1.76	2.12	n.s.	9.24
NWMN_RS13355	Gamma-hemolysin HlgAB/HlgCB subunit	2.63	3.80	n.s.	4.63
NWMN_RS13345	Gamma-hemolysin HlgAB subunit A	3.56	3.80	n.s.	n.s.
NWMN_RS13335	Immunoglobulin-binding protein Sbi	n.s.	3.03	n.s.	5.41
NWMN_RS13795	Fibronectin-binding protein FnbB	n.s.	3.24	n.s.	n.s.
NWMN_RS04280	MSCRAMM family adhesin clumping factor ClfA	3.93	3.06	n.s.	n.s.
NWMN_RS10800	Chemotaxis inhibiting protein Chp	n.s.	0.53	-	-
Nucleotide biosynthesis pathway				
NWMN_RS00455	Purine nucleoside phosphorylase DeoD	6.63	5.33	4.68	10.09
NWMN_RS00085	Adenylosuccinate synthetase PurA	2.00	1.85	1.75 (*P* = 0.1)	2.21
NWMN_RS07050	GMP reductase GuaC	2.42	2.18	4.19	3.69
Nitrogen cycle metabolic process				
NWMN_RS13190	GAF domain-containing protein	4.45	4.65	n.s.	n.s.
NWMN_RS13195	Respiratory nitrate reductase subunit gamma NarI	4.34	4.16	n.s.	n.s.
NWMN_RS13200	Nitrate reductase molybdenum cofactor assembly NarJ	5.68	4.96	n.s.	n.s.
NWMN_RS13205	Nitrate reductase subunit beta NarH	6.04	5.60	n.s.	n.s.
NWMN_RS13210	Nitrate reductase subunit alpha NarG	6.64	6.26	n.s.	n.s.
NWMN_RS13215	Uroporphyrinogen-III C-methyltransferase CobA	6.99	5.80	n.s.	n.s.
NWMN_RS13220	Nitrite reductase small subunit NirD	9.74	4.93	n.s.	n.s.
NWMN_RS13225	NAD(P)/FAD-dependent oxidoreductase NasD	26.63	13.53	n.s.	n.s.
NWMN_RS13230	Sirohydrochlorin chelatase SirB	22.27	10.99	n.s.	n.s.
Amino acid metabolism				
NWMN_RS04695	Argininosuccinate synthase ArgG	2.71	3.37	n.s.	n.s.
NWMN_RS06110	Ornithine carbamoyltransferase ArgF	4.63	3.67	3.36 (*P* = 0.1)	20.68
NWMN_RS06115	Carbamate kinase ArcC1	2.40	2.79	3.77	20.56
NWMN_RS07005	Threonine synthase ThrC	1.96	2.89	n.s.	n.s.
Fatty acid and lipid metabolism				
NWMN_RS06420	Fatty acid synthesis transcriptional factor FapR	0.63	n.s.	0.45	0.51
NWMN_RS04800	3-oxoacyl synthase FabH	0.56	n.s.	0.55	0.59

a n.s., statistically not significant (*P* > 0.05 or |L2FC| < 0.58); -, not present in the genome.

**FIG 2:**
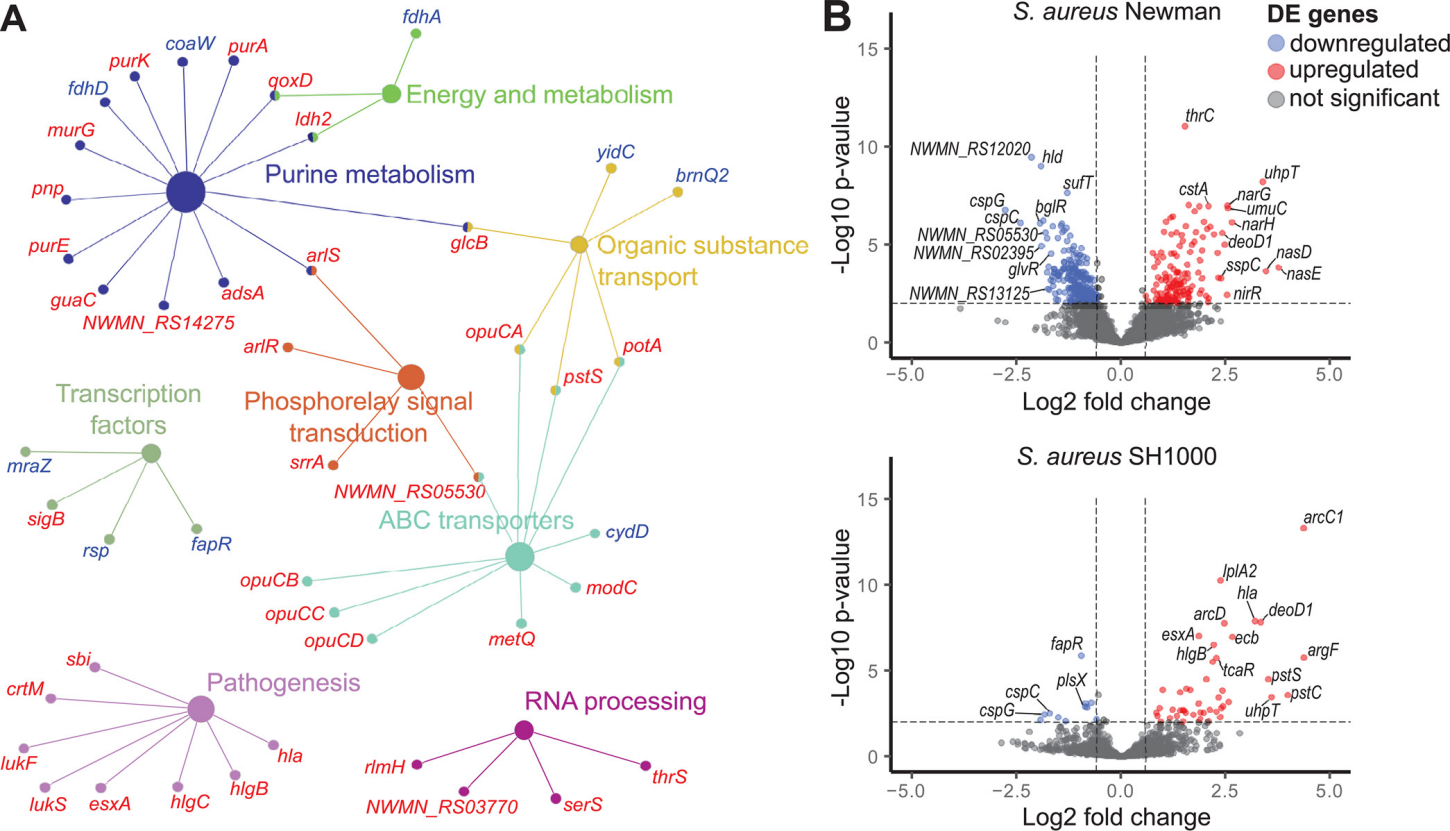
Differential expression of host S. aureus genes. (A) Gene ontology pathway enrichment analysis of downregulated (blue) and upregulated genes (red) in phage K-infected S. aureus Newman and SH1000 (|L2FC| < 0.58 and *P* < 0.05). The DE genes were involved in 8 gene ontology groups defined by ClueGO and KEGG, which are indicated with different colors. (B) Volcano plots showing DE genes of S. aureus Newman and SH1000 20 min post phage K infection compared to uninfected control. Points above the horizontal dashed line represent significantly expressed genes with *P* < 0.05. The total number of analyzed genes was 2815 strain Newman, and 2479 strain SH1000 and they are represented by the average L2FC values.

Functional associations of the bacterial DE genes were analyzed in the interaction network (Fig. 2A). An immediate impact on the abundance of host transcripts relative to each other was observed within 2 and 5 min postinfection with upregulation of the transcriptional regulator *sigB* and downregulation of genes involved in stress response, cold shock protein genes *cspG* and *cspC*, repressor *lexA*, and transcription regulator for a cell division *mraZ*, and *rsp* AraC-type regulator. From 10 min postinfection, the genes involved in cellular processes and transport, the panthotenate kinase *coaW*, acyphosphatase *acyP*, and of fatty acid metabolism were particularly downregulated. The upregulated genes in the first 10 min postinfection influence nucleotide synthesis pathways (*purAFM*, *guaC*, and *pyrFE*), arginine biosynthesis (*argGF*, *ArC1*, and *carB*), oxidative stress response (*ybaK*, *csbB*, and *trxA*), energy metabolism, and the choline ABC transport system *opuABCD*. Within 20 min, the phage infection caused the downregulation of the gene *ung* for uracil-DNA glycosylase and *rpsL* 30S ribosomal protein S12.

Virulence genes, namely, leucocidin components *lukF-G* and *lukS-H*, hemolysins *hlgCB* and *hly*, and virulence factor *sasH* were upregulated in both strains after 10 min of phage K infection (Table 2). Within 20 min, immunoglobulin-binding protein *sbi* expression, which protects the bacterium from the immune system also increased. Interestingly, the SAV0808 homolog encoding a hypothetical protein localized near the SaPI attachment site and the methyltransferase gene *rlmH* encompassing the staphylococcal cassette chromosome *mec* (SCC*mec*) attachment site showed higher transcription, too (Table S4).

In comparison to SH1000 in strain Newman (Table 2), additional genes for transcriptional regulators *saeS*, *sarA*, *sarS* and *yydK*, xenobiotics efflux *corA* and *mntH*, biofilm formation regulator *icaR*, and cell wall synthesis *fmtA*, *ugtP*, and *femB* were downregulated (Fig. 2B; Table S4). Numerous genes involved in *de novo* pyrimidine biosynthesis, amino acid biosynthesis pathways and genes ensuring energetic metabolism, including tricarboxylic acid (TCA) cycle were upregulated. In contrast to Newman, in strain SH1000, transcription of the regulator *sarV*, involved in virulence and autolysis, was upregulated. Strikingly, some of the *nir* and *nar* genes involved in dissimilatory nitrate metabolic process (Table 2) responded very differently during the phage K infection, showing an immediate decline in SH1000, compared to a significant increase in transcript level in strain Newman.

### Interaction of lytic phage with prophages.

Integrase genes from S. aureus Newman prophages Sa7int of φNM2, Sa5int of φNM1, and Sa3int of φNM3 and phage antirepressors were upregulated from 5 min after phage K infection. Later, from 10 to 20 min, there was an increased transcription of genes for phage structural proteins: baseplate tip from φNM2 and φNM1, phage tail tape measure and major capsid protein from φNM3. The chemotaxis-inhibiting protein gene *chp* from the immune evasion cluster of phage φNM3, a virulence factor, was downregulated from 20 min postinfection. Integrase type Sa6int φNM4 did not exhibit deregulation of any of its genes except for NWMN_RS01645 encoding a hypothetical protein.

## DISCUSSION

In this work we show that phage K follows a mostly host-independent transcriptional strategy, which is consistent with findings in transcriptomic studies of other large and midsized myoviruses infecting Staphylococcus (20), Pseudomonas (23, 24) and *Synechococcus* (25). Earlier release of phage K offspring with higher burst size in S. aureus SH1000 compared to Newman points to more effective host takeover in this strain. Early expressed genes are generally considered to play a role in the takeover of bacterial metabolism, but we found that previously identified host takeover genes were expressed during the middle transcription phase. Therefore, we hypothesize that some of the very early expressed genes are responsible for manipulating the phage genome inside the bacterium and establishing the phage replicating complex (26).

The slowdown of transcription from early and middle promoters and some bacterial operons is aided by antisigma factor Asf gp137 inhibiting σ^70^ (27) and/or a transcription factor binding to the conserved −35 region in the σ^70^ promoter sequence. Candidate inhibitors are gp126, coding for a protein with a DNA binding domain, and gp201 encoding a putative Cro/CI type repressor. The transition to late expression is likely regulated by the gene gp117, which is homologous to *Bacillus* phage SPO1 gene gp34 and encodes an alternative RNA polymerase sigma factor (19).

Noncoding RNAs participate in gene expression regulation (28), but it has also been difficult to ascertain function for the phage lncRNAs and sncRNAs in previous studies (23, 24, 29). Two lncRNAs in phage K genome resemble GOLLD and ROOL ribozymes from *Lactobacillus* prophages (30, 31) by their location relative to tRNA genes and by their abundant transcription. The role of the phage K lncRNAs could be also related to bacterial survival under stress conditions, as described in extremophilic bacteria OLE RNA (32), or may be linked to the phage genome copy number, which is the case for lncRNA expressed from a Lactobacillus salivarius megaplasmid (31).

Approximately one-quarter of the CDS of S. aureus was up- or downregulated relative to other host transcripts during phage K infection (Table S4) and the differential gene expression observed among bacteria is comparable to the previously described transcriptomic interactions between phage and host (24, 29, 33–35). As with phage K, other myoviruses upregulated purine and pyrimidine biosynthesis pathway genes (24, 34–36), whereas lytic infection by a temperate siphophage infection did not affect the genes in those pathways (33). The arginine biosynthesis pathway upregulation detected during phage K infection was also found in T4-like infection of Campylobacter jejuni (35), while it was not reported in other phages (24, 36). The upregulation of bacterial biosynthetic pathways does not necessarily have to be triggered by phage effectors; bacteria can also respond to the lack of dNTPs and amino acids needed for the synthesis of phage building blocks. On the other hand, Rees and Fry described the bacterial chromosome degradation from 5 min of phage K infection (37), thus the observed differences between the levels of particular mRNAs at later infection times reflects the differences in the stability of such mRNAs. Eventually, the upregulation of DE host genes could comprise new transcripts from the remnants of host DNA, where the transcription is not blocked by the phage. The degradation products from host chromosome are incorporated into phage DNA (37), their larger amount in the cytoplasm may be also related to DE of genes necessary for their utilization. The differential expression of the host genes that we observe is comparable to transcriptomic data describing general S. aureus response to DNA damage (38), characterized by upregulation of *recA* and *uvrABC* and downregulation of *lexA* genes. Candidate phage K nuclease genes involved in DNA degradation are gp140, gp138, gp111, and gp086.

The bacterial gene expression is controlled by global regulatory mechanisms, i.e., two-component systems, transcription factors, the alternative sigma factor SigB, and sncRNAs (39–41), thus S. aureus field strains exhibit extreme variability in protein expression (42). The higher number of DE genes in strain Newman could be partially assigned to its missense mutation in the *saeS* gene for histidine kinase involved in the expression of exoproteins related to adhesion and invasion, while SH1000 with lower number of DE genes produces truncated TcaR, which plays a minor role in exoprotein expression regulation (43). The DE of virulence-related genes did not clearly identify the involvement of one single global regulator and did not correspond with the detected low upregulation of ArlRS, and SrrAB and downregulation of AgrAC. This suggests that the phage targets virulence gene expression in a specific way, with the possible involvement of unknown regulators, and is thus able to overcome a wide variety of staphylococcal strains.

Most S. aureus isolates are lysogenic carrying one to four prophages, which have an impact on pathogenesis (44). Therefore, a better knowledge of the interaction of prophages with lytic phages is important for phage therapy. We hypothesize that the higher number of DE genes in strain Newman could be an emergent property off competition between phage K and the resident prophages. Chen et al. (45) showed that the induced *pac*-type phages φNM1, φNM2, and φNM4 of strain Newman facilitate amplification of the host DNA flanking the prophage, which could lead to horizontal gene transfer by lateral transduction (46, 47). Moreover, the temperate phages play a key role in the spread of the SaPIs (48, 49) and the generalized transduction of other mobile genetic elements (50, 51). Interestingly, sncRNA004 that we identified in phage K targets the conserved sequence of mRNA that encodes the FibU upper baseplate protein of φNM1, φNM2, and φNM4, which could block the translation of this protein important for virion assembly. FibU gene is homologous to the ORF68 from S. aureus phage 80α (52). The existence of a defense mechanism of lytic phages targeting crucial component of the temperate phages indicates ongoing arms race between the two phage groups. The temperate phages sense their density in bacterial communities (53) and a yet unknown analogous mechanism could be responsible for the recognition of lytic phages. During lytic infection with phage K, only a few prophage genes were upregulated before the entire bacterium was lysed, indicating that the prophage was unable to complete its replication and packaging of its own or foreign DNA. The observed response of the cell culture infected with phage K at high MOI should be verified at low MOI.

Although much work remains to be done to evaluate the safety of phage therapeutics, our transcriptomic data provide important insight into their rational use. Our results show that kayviruses used empirically for treatment over a long period of time do not cause adverse effects in S. aureus that would impede their use. The uncovered phage-host interactions could provide new impetus for the search for new antibacterial targets. The next important step in phage research is an assessment of the interactions between phage-bacteria and the human immune system at the transcriptomic, proteomic and metabolomic levels.

## MATERIALS AND METHODS

### Bacterial and bacteriophage strains and culture condition.

The Staphylococcus aureus strains RN4220 (54) and SH1000 (55) were used and strain Newman (56) was obtained from S. Foster (The University of Sheffield, United Kingdom). S. aureus strains were routinely grown in meat-peptone broth (MPB) on meat peptone agar (MPA) plates prepared according to Botka et al. (8). Bacteriophage K was obtained from Prof. C. Wolz (University of Tübingen, Germany). Phage K was propagated from sterile stock lysate stored at 4°C. Large-scale phage K propagation on S. aureus strains was done in 250 mL MPB (8).

### Phage Growth and Adsorption Assays.

The adsorption efficiency of phage K on S. aureus strains SH1000 and Newman was determined at an MOI of 0.1. The counting of PFU/mL was done using the soft agar overlay method, where 100 μL of the overnight culture supplemented with 10 μL CaCl_2_ (0.02 M) and 100 μL of the phage suspension were added to 2.5 mL of the 0.7% MPA, and then poured onto an MPA plate. The adsorption was calculated by determining the PFU of the unbound phage in the supernatant and subtracting it from the total number of input PFU. Experiments were carried out in triplicate.

The one-step growth curve of phage K was determined on S. aureus Newman and SH1000, which were infected at the early exponential phase (OD_600_ = 0.3) at an MOI of 0.1, and incubated at 37 °C with shaking (150 rpm). To remove non-adsorbed phages, after 10 min of incubation, the mixture was centrifuged at 10,000 × *g* for 3 min, and the pellet was resuspended in 10 mL of the MPB. The samples of 10 μL were taken at 0, 5, 10, 15, 20, 25, 30, 35, 40, 45, 50, 55, 60, 70, and 80 min postinfection. The PFU counts were determined using the soft agar overlay method. Experiments were carried out in triplicate.

### Whole-genome sequencing of S. aureus SH1000.

Genomic DNA of S. aureus strain SH1000 was isolated using a High Pure PCR Template Preparation kit (Roche) according to the manufacturer’s instructions with 5 μg/mL lysostaphin (Sigma-Aldrich) added to the suspension buffer. The 400-bp sequencing library was constructed using an Ion Plus Fragment Library kit (Thermo Fisher Scientific). The whole-genome sequencing was performed in an IonTorrent PGM (Thermo Fisher Scientific) using an Ion 318 Chip v2. The quality of the WGS single-end reads (714,872) was assessed using FastQC. The raw reads were error corrected and assembled using SPAdes v3.13.0 (57) (-k 21,33,55,77,99,127, -iontorrent, -cov-cutoff auto). Gene prediction and annotation for transcriptome analysis were performed using RAST v2.0 (58) (genetic code 11, RASTtk annotation scheme). Sequences were manipulated and examined in the cross-platform software Ugene v40.0 (59).

### RNA extraction and rRNA depletion.

For RNA sequencing, bacterial strains S. aureus Newman and SH1000 at an early exponential phase (OD_600_ = 0.35) were infected with phage K (MOI = 7) and incubated at 37°C with shaking (180 rpm). 2-mL aliquots of cell suspension were taken periodically at 0, 2, 5, 10, 20 and 30 min, and centrifuged at 11,000 g for 1 min. The supernatant was discarded, and pellets were snap-frozen in liquid nitrogen and stored at −70°C. Three biological replicates were analyzed for each time point during phage K infection, and were used for RNA extraction. The pellets were resuspended in 1 mL of TRIzol Reagent (Sigma-Aldrich), and cells were lysed using Lysing Matrix B (MP Biomedicals) in a FastPrep-24 Classic bead beating grinder and lysis system (MP Biomedicals) for 6 × 20 s. The total RNA was extracted according to the TRIzol Reagent manufacturer's recommendations. Genomic DNA was removed using a Turbo DNA-free kit (Thermo Fisher Scientific). Total nucleic acids were quantified at 260 nm in a NanoDrop 2000/2000c Spectrophotometer (Thermo Fisher Scientific), and RNA integrity was measured using a 2100 Bioanalyzer Instrument (Agilent). The rRNA (rRNA) depletion of all RNA samples was performed using a RiboMinus Bacteria 2.0 Transcriptome isolation kit (Thermo Fisher Scientific) according to the manufacturer’s instructions. The ribodepleted RNA was precipitated with glycogen (Thermo Fisher Scientific) using the manufacturer’s protocol, after that RNA integrity was measured again.

### RNA-sequencing.

Ribodepleted samples were converted to sequencing libraries using a NEBNext Ultra II Directional RNA Library Prep kit for Illumina kit (New England Biolabs), following the standard protocol. Libraries were sequenced at the Genomics Core Facility of CEITEC MU (Central European Institute of Technology, Masaryk University), by Illumina NextSeq500 single-end runs 500 using 75 cycles high-output chemistry.

### Bioinformatic analyses.

Phage K CDS homologs were searched using tblastn (database: nonredundant nucleotide collection), the CDD webserver (https://www.ncbi.nlm.nih.gov/Structure/cdd/wrpsb.cgi, database: CDD v3.19 – 58,235 PSSMs) and InterProScan v5 (60). Promoter sequences were predicted using the PePPER webserver (61). To detect promoters of late transcribed genes in the phage K genome, 250-bp sequences upstream and 50-bp downstream from start codons were scanned using the MEME Suite v5.4.1 (62) with default parameters. Sequence logos were generated with WebLogo v2.8.2 (63). Terminator sites were predicted using ARNold (64) and marked ends of transcription without predicted terminator were searched for GC-rich hairpin with stem-loop using RNAfold (65).

The presence of sncRNAs in the phage K genome was investigated with the computational tool nocoRNAc v1.23 (66) using the standard protocol. A sequence predicted as a putative sRNA was considered expressed when the minimum read coverage mapped to the correct strand was 5-fold. Possible interaction partners of the sRNAs were searched in the S. aureus Newman genome and in the phage K genome with the program IntaRNA v2.0.0 (67) using default parameters with the threshold set to *P* < 0.001.

The RNA-Seq raw sequence (11,951,019 ± 1,304,445 per sample) quality was assessed using FastQC v0.11.5 (https://www.bioinformatics.babraham.ac.uk/projects/fastqc/) both before and after quality trimming and filtering for quality, which were done using the Trimmomatic toolkit v0.36 (68) with the following parameters: ILLUMINACLIP:TruSeq3-SE:2:30:10 SLIDINGWINDOW:4:15 LEADING:22 TRAILING:22 MINLEN:20 AVGQUAL:22. Trimmed reads were aligned to the sequences of the reference genome of S. aureus Newman, SH1000, and phage K genomes using STAR v2.5.2b with the following parameters: outFilterMultimapScoreRange 0, outFilterMatchNmin 30, outFilterMatchNminOverLread, 0.95, outFilter-Nmax 999, outFilterMismatchNoverLmax 0.02, outFilterMismatchNoverReadLmax 1, alignIntronMin 20, alignIntronMax 1. Next, the alignment files were processed using Samtoools v1.9 (69) to create sorted BAM files, which were visualized in Tablet v1.21.02.08.

Reads aligning to the annotated features of the phage K genome and non rRNA or tRNA genes in S. aureus Newman and SH1000 were used for differential expression analysis in the statistical language R v4.1.1 (70) using the DESeq2 package v1.32.0 (21) at FDR (false discovery rate) <0.05. The hierarchal clustering and heatmap generation were performed from log-transformed normalized counts per gene in the package ComplexHeatmap v2.8.0 (71). The functional analysis and gene ontology classification of expressed genes was performed using Panther v16.0 (72), KEGG v98.0 (73) and the ClueGO v2.5.8/CluePedia v1.5.8 plugin of the software Cytoscape v3.9.0 (74–76). Details of specific statistical analyses are described in the relevant figure legends.

### Data availability.

The Whole Genome Shotgun project of the S. aureus strain SH1000 has been deposited in NCBI database under the Bioproject accession number PRJNA769253. The RNA-Seq data were deposited in GEO under accession GSE190637. The deposited data are publicly available as of the date of publication.
