# Supplementary material for: Global Transcriptomic Analysis of Bacteriophage-Host Interactions between a Kayvirus Therapeutic Phage and Staphylococcus aureus
Source: Microbiol Spectr. 2022 Apr 18;10(3):e00123-22. doi: 10.1128/spectrum.00123-22 (PMC9241854; doi:10.1128/spectrum.00123-22)
Supplement: SUPPLEMENTAL FILE 1 — Supplemental material. Download spectrum.00123-22-s001.pdf, PDF file, 0.8 MB [file spectrum.00123-22-s001.pdf]

TABLE S1 Genomic Properties of Host Strains Used in This Study

**Organism name**	***S. aureus* SH1000**	***S. aureus* Newman**
GenBank Accession	JAJAFP000000000	NC_009641.1
Genome sequence reference	In this study	Baba et al. (1)
Genome size (Mbp)	2.68	2.89
No. of protein-coding sequences	2484	2614
%GC	32.8	32.9
Clonal complex (WGS-based clade)	CC8a	CC8b
SCC*mec* type	None	None
Prophages	None	4
Genomic islands	4	4
Plasmids	None	None
SaPI	None	None
Regulators	*truncated TcaR*	saeRS constitutively expressed by SaeS T53C substitution, fnbA and fnbB mutated
No. of RNAs	74	73

REFERENCE

1. Baba T, Bae T, Schneewind O, Takeuchi F, Hiramatsu K. 2008. Genome sequence of *Staphylococcus aureus* strain Newman and comparative analysis of staphylococcal genomes: polymorphism and evolution of two major pathogenicity islands. J Bacteriol 190:300-310. https://doi.org/10.1128/JB.01000-07.

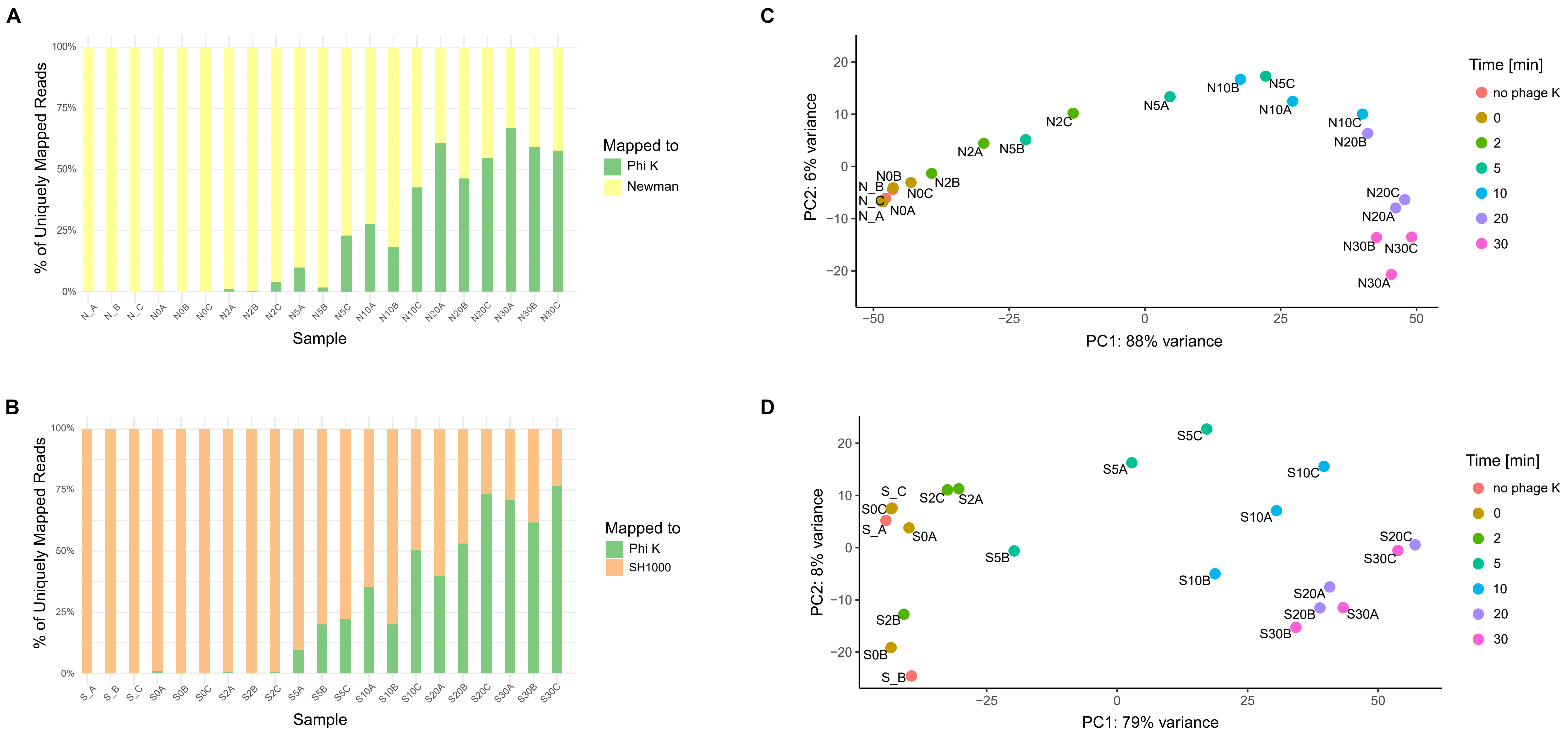

FIG S1 RNA-Seq Data Analysis

Percentage of uniquely mapped reads aligning to the phage K (Genbank Accession: NC_005880.2) and *S. aureus* Newman (**A**) and SH1000 (**B**) genome (Genbank Accessions: NC_009641.1 and JAJAFP000000000.1) at different time points post-infection. Principal component analysis (PCA) graph for RNA-Seq samples from *S. aureus* Newman (**C**) and SH1000 (**D**) infected with phage K. The individual samples are designated S for strain SH1000 and N for strain Newman, the number 0, 2, 5, 10, 20, and 30 stands for time (min) of sampling, the samples taken before phage K addition are designated N_ or S_. The biological replicates are distinguished by capitals A, B, and C.

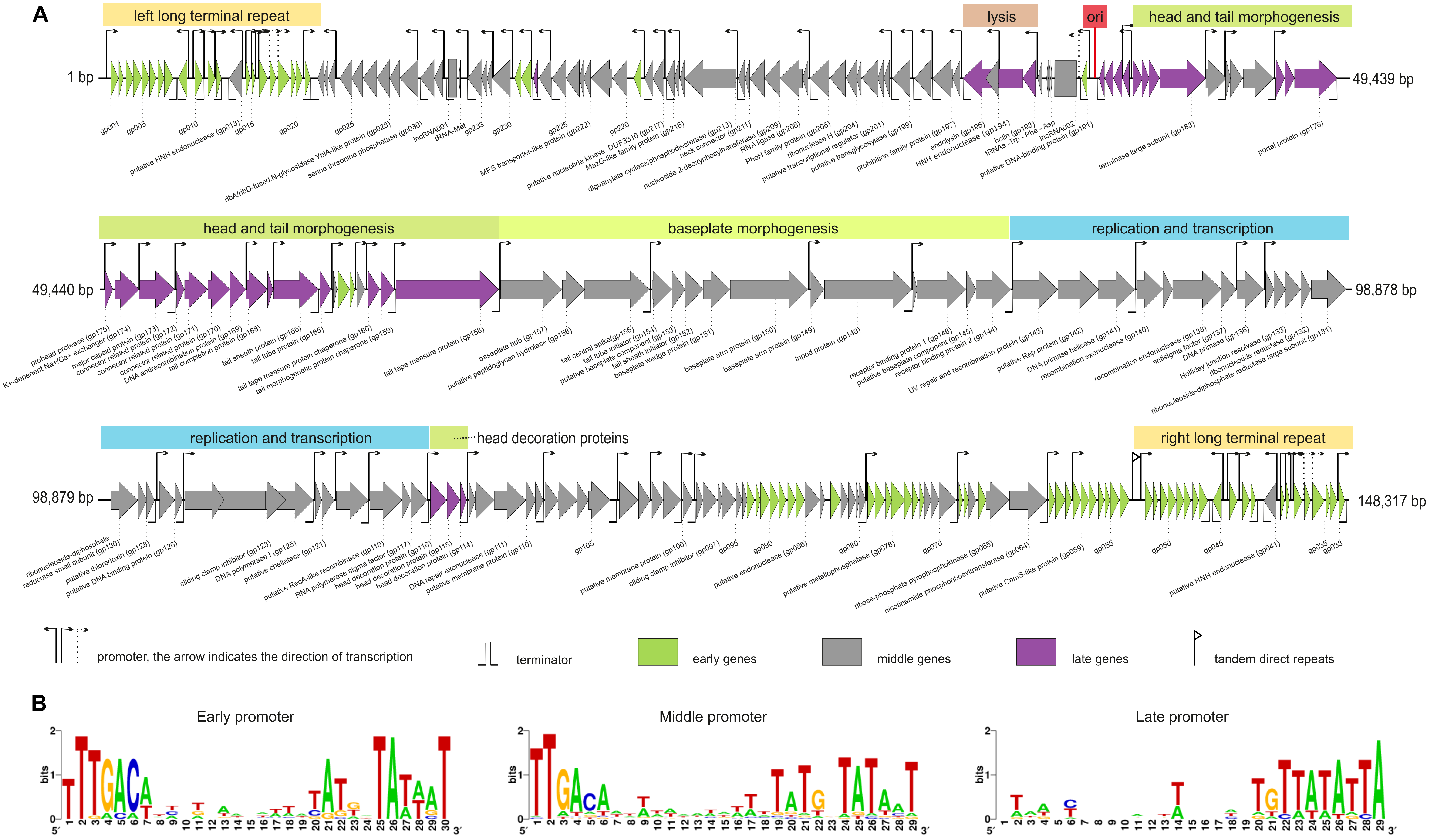

FIG S2 Phage K Genetic Map and Promoters

(**A**) Position of promoters and terminators in phage K genome (Genbank Accession: NC_005880.2). The genetic map of the phage K genome is indicated by arrows showing the direction of transcription. The genome modules with known functions are depicted with colour boxes above the ORF map. The genes are coloured based on their transcription phase – early, middle and late. (**B**) Consensus sequence weblogos of early, middle, and late promoters. Grouping of promoters according to their time of appearance during transcription revealed consensus sequences at - 35 region (TTGACA) and Pribnow box (TATAAT) in early and middle promoters. The late promoters have conserved only -10 region with TGTTATATTA motif.

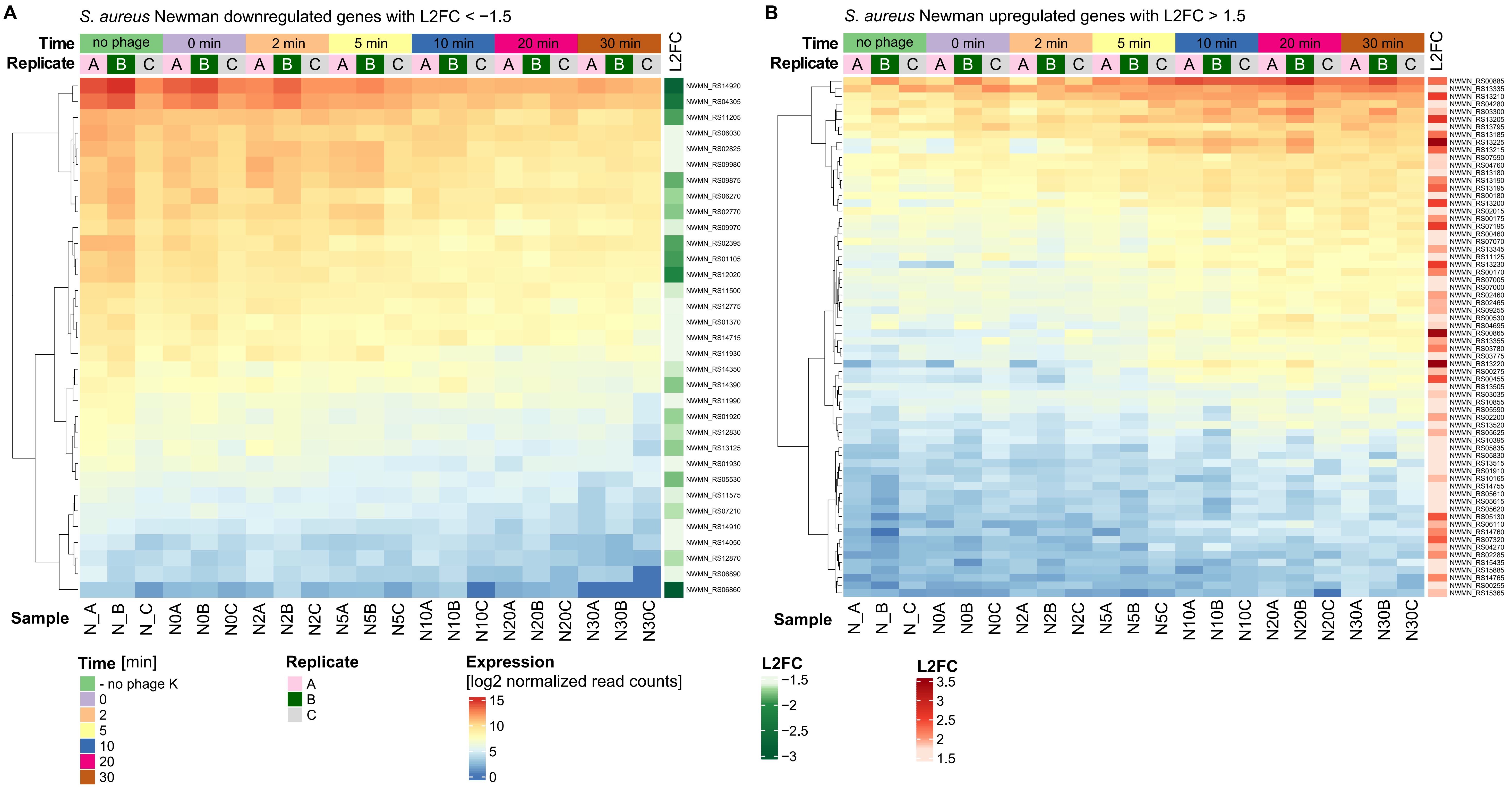

FIG S3 Hierarchical Clustering Heatmaps of Differentially Expressed Genes of *S. aureus* Newman

Differentially expressed genes with |L2FC| > 1.5 at 30 min after phage K infection with $p < 0.05$ were selected. The heatmap was created using ComplexHeatmap R package from log$_2$-transformed normalized counts per gene generated using DESeq2 as described in Materials and Methods. The columns represent different biological replicates (A, B, and C) of *S. aureus* Newman samples taken before phage K addition (designated N_) and in time 0, 2, 5, 10, 20, and 30 min after phage K addition (designated N0 – N30). (**A**) Downregulated genes with L2FC < 1.5, value of L2FC is depicted by green scale in the last column. The downregulated genes were divided into four clusters by initial transcript abundance and by the time when the expression change occurs. (**B**) Upregulated genes with L2FC > 1.5, value of L2FC is depicted by red scale in the last column. Upregulated genes clustered into three groups. Genes with high initial transcript count show an expression increase within 5 to 20 min followed by a decrease in the late phase of the phage infection. Second cluster of upregulated genes is distinguished by significant rise within 10 min post infection and the last cluster includes genes with a low initial number of transcripts that gradually increase during the phage life cycle.

TABLE S2A **Promoters of bacteriophage K**

Promoters were predicted using the Pepper webserver. Marked starts of transcription without predicted promoters were inspected 250-bp upstream and 50-bp downstream from start codon using the program MEME as described in Materials and methods.

#	Position	Strand	Gene promotor	Sequence	Transcription phase	Prediction
1	425	+	gp001	TTGACAACTATGAAGCGGGTATGCTATAAT	early	yes
2	3586	-	gp009	TTGACTTCTGAATAACTATACTGTAATAT	early	yes
3	3709	+	gp010	TTGACTTTATTATCATATGGTAGTAATAT	early	yes
4	4219	+	gp011	TTGACACCTTACAAGATACATGTTATTAT	early	yes
5	5691	-	gp013	TTGACATTAAGACCGAATTATTATATAAT	middle	yes
6	5765	+	gp014	TTGACTTTAATATCATTATAGTTTAATAT	early	yes
7	5988	+	gp015	TTGACAACCTAGAAACAACATGTTAATAT	early	yes
8	6189	+	snc003	TTGACAACTTAAACACTACATGTTATTAT	early	yes
9	6285	+	gp016	TTGACAGTCACTTGAAACCATGATATTAT	early	yes
10	6690	+	gp017	TTGACTTTCAAGCCCTACCATGTTATTAT	early	yes
11	7021	+	gp018	TTGACATCCTAACATATAGATGGTAATAT	early	yes
12	8082	+	gp021	TTTACAATCTTTTAGTTTGTATGATATAAT	early	yes
13	9588	-	gp024	TTATCATTTATAGTATTTGTTGCTGTAAA	middle	no
14	12635	-	gp030	TTGACTTTTTTTACTAAGTATGGTAAGAT	middle	yes
15	13630	-	gp032	TTAAACTAATAGGTGTTTTTTTGTTATAT	middle	no
16	14555	-	gt001	TTGACATTAAGTATAATTTATGGTATATT	middle	no
17	15832	-	gp231	TTGTTAAGTTTTATACATTCTATAATCAT	middle	no
18	16303	-	gp230	TTGACATTATTATCAATATATGTTATTAT	middle	yes
19	17113	-	gp228	TTGACAAAATATAAAAAATAGTGTATAGT	early	no
20	17901	-	gp226	TTAATAAGTCTGCTTTTCTCTTATATTAG	middle	no
21	21456	-	gp219	TTGACAAATACAAATACTTGTAATATAAT	early	yes
22	22332	-	gp217	TTGTAGCCAAGCAGGGTGTTTTTTTTTAT	middle	no
23	22950	-	gp215	TTGACAATAGTATCATAATATGATATAAT	middle	yes
24	25219	-	gp213	TTGACAAATATTATTTACTATGGTATGAT	middle	yes
25	28067	-	gp207	TTGACAAATCCCCTTAGTTATGGTATAAT	middle	yes
26	31280	-	gp200	TTGACTTCATAAGTTAACTATGCTATAAT	middle	yes
27	32109	-	gp199	TTGCGTTATTTAAAGATATATGTTATGAT	middle	no
28	32893	-	gp198	TTGACATAGGTGGTTTTTTATGCTATAGT	middle	no
29	34069	-	gp196	TTGACAAAATTAAATACATAGTGTATAGT	middle	no
30	35571	-	gp194	TTGACAACATAATAACTTTCCTATATACT	middle	yes
31	37055	-	gp193	ATAAACATGACCGACCTACTGTTATATTA	late	no
32	38602	-	lnc002	TTGACAAGTAATAAAAATTATGTTATAAT	middle	yes
33	39090	-	gp191	TTGACTTATTTATCAATATAGTATATAGT	early	yes
34	40448	+	nc005	TTTAAATTTACTTATTTTGTGGTATAAT	middle	yes
35	40779	-	gp187	TTTTATGACTGGCTTTTAATGTTATATTA	late	no
36	43757	+	gp182	TTGACAGATAAATTATTTTATGGTACAAT	middle	yes
37	44504	+	gp181	TTAAGAAAAAAAGATGTAAAACAATTCTT	middle	no
38	46428	+	gp178	ATTACTCCTCTTTTTTGTTTGCTATATTA	late	no
39	49058	+	gp175	TTCACTTACAAGTTATTATTGTTATATTA	late	no
40	50872	+	gp173	TAATTAATGCACAAAGTTGTGTTATATTA	late	no
41	52417	+	gp172	TAAACTTAGGGTTTATCCCTTTTTTATTA	late	no
42	55174	+	gp168	ATGAACTGGAAGATGAAAAATTTAAATTA	late	no
43	56251	+	gp166	CTAGGTAGGAAATTAGATATATAATAACA	late	no
44	58601	+	gp164	TTGACACTTTAAAATTTATATGTTATTAT	middle	yes
45	59530	+	gp161	TTGACAAATTAAAACTAATAAATTATAAT	middle	yes
46	59946	+	gp160	GTAAGCCTACACTAGTCCGTGTTATATTA	late	no
47	61042	+	gp158	GAAGACCTAGATGATGACTGGTATATGTA	late	no
48	65195	+	gp157	TTGACACAAGAGTAGTATCATAGTATACT	middle	yes
49	71171	+	gp154	TTGTAATTTAAACTAGTTCGTGATATATT	middle	no
50	77412	+	gp149	TTGACAGAAAGTTAATAATATGGTATACT	middle	yes
51	81525	+	gp147	TTGATTGACCCCTCTTTATTTAATAAG	middle	no
52	85456	+	gp143	TTGACTTGAAAAGGATTCTGTGGTATACT	middle	yes
53	90343	+	gp140	TTGACATTTTATATGTTAGGTGGTATAAT	middle	yes
54	94281	+	gp136	CTTAGGACAAATTTATAATATGTTGTTAT	middle	no
55	95417	+	gp135	TTGACCTTAGAGAAGTTTTATGTTATACT	middle	no
56	101120	+	gp127	TTGGAATTTCCTACTATCTGTGCTATACT	middle	no
57	102253	+	gp125	TTGACAAGGTTTAAAATATATGGTATAGT	middle	no
58	107419	+	gp122	TTGACAATATAGTTAACTTATGTTATACT	middle	yes
59	108242	+	gp120	TTGACAAATATAAAAAACTATGTTATAAT	middle	yes
60	109609	+	gp119	TTGAGATATAATTACTAGGAGGATATTAA	middle	no
61	111899	+	gp116	AGTTACAGTGCCTTCCTTGTGTTATATTA	late	no
62	113473	+	gp113	TTGACAATTTATAATATCTATGATACACT	middle	yes
63	116490	+	gp108	TTGACTCTTTTTACTATATATGGTATATT	middle	yes

TABLE S2A continued

#	Position	Strand	Gene promotor	Sequence	Transcription phase	Prediction
64	119249	+	gp104	ATGAAAAGTTAAAATATCTTTTAGGTAAA	middle	no
65	120704	+	gp102	TTGACAGCTCCTATAGTTTATGATATAGT	middle	no
66	121927	+	gp100	TTAATAGTAGTCTCCTCTTATATTATAAT	middle	no
67	122483	+	gp099	TTGACTCTCTTTTTGTTTTATGGTATATT	middle	yes
68	129226	+	gp079	TTGACATTAGGTTTCTTTTATTATATACT	early	yes
69	132831	+	gp069	TTGACAGCAGGTATTTTTTATAGTATACT	early	yes
70	136385	+	gp063	TTGACAAAGGGAGTTTTTTATTATATAGT	early	no
71	137365	+	gp060	TTGACTTAGGTAGATACTTATTATATAAT	early	yes
72	140256	+	gp053	TTGACAACTATGAAGCGGGTATGCTATAAT	early	yes
73	143417	-	gp045	TTGACTTCTGAATAACTATACTGTAATAT	early	yes
74	143540	+	gp044	TTGACTTTATTATCATATGGTAGTAATAT	early	yes
75	144050	+	gp043	TTGACACCTTACAAGATACATGTTATTAT	early	yes
76	145522	-	gp041	TTGACATTAAGACCGAATTATTATATAAT	middle	yes
77	145596	+	gp040	TTGACTTTAATATCATTATAGTTTAATAT	early	yes
78	145819	+	gp039	TTGACAACCTAGAAACAACATGTTAATAT	early	yes
79	146020	+	snc010	TTGACAACTTAAACACTACATGTTATTAT	early	yes
80	146116	+	gp038	TTGACAGTCACTTGAAACCATGATATTAT	early	yes
81	146549	+	gp037	TTGACTTTCAAGCCCTACCATGTTATTAT	early	yes
82	146852	+	gp036	TTGACATCCTAACATATAGATGGTAATAT	early	yes
83	147913	+	gp033	TTTACAATCTTTTAGTTTGTATGATATAAT	early	yes

TABLE S2B Terminators of bacteriophage K

Terminators were predicted using the ARNold webserver. Sequences with marked ends of transcription without predicted terminators were searched for typical GC-stem loop using the RNAfold websuite as described in Materials and methods.

#	Position	Strand	Gene terminator	Sequence	Prediction
1	3067	+	gp008	CAGGTGTCTATATATATATATAGACGGTTG	no
2	3123	-	gp009	TACACTGGGAATAATATCCTAGTGTA	yes
3	4128	+	gp010	GTTGCAATCCTCAAGCATCTATAGTAATATAATAGGTGTAGGGGATAGCAAC	no
4	4874	+	gp012	TCCCTAGGATTAGATTTCTAGGGA	yes
5	5150	-	gp013	GAAAAGGGTTGACCTTTTC	yes
6	8370	+	gp021	GAGGGAATAAAATCCCTC	yes
6	8370	-	gp022	GAGGGATTTTATTCCCTC	yes
7	12672	-	gp031	CACCTATTAATTTAATAGGTG	yes
8	13644	-	lncRNA001	CACCTATTTAAACTAATAGGTG	yes
9	14563	-	gp233	ATAAATTAACTTGACATTAAGTATAATTTAT	no
10	17150	-	gp227	GGCTACTTTAATTAGTAGCC	yes
11	17917	-	gp225	GCAGACTTTTAATAAGTCTGC	yes
12	21525	-	gp218	TACCTTACCCTATGTTAAGTTATAGGTGTAAGGTA	yes
13	22343	-	gp216	CACCTTGCTTGTAGCCAAGCAGGGTG	yes
14	25256	-	gp212	GAAGGACTTTAAAAAGTTCTTC	yes
15	28048	-	gp206	CCTTAGTTATGGTATAATTATTCTATAATAACTAAGG	yes
16	31327	-	gp199	GACTAAGATTAATTTCTTAGTC	yes
17	32221	-	gp198	GAGTGGTAAATATAATTACCTCTC	yes
18	32909	-	gp197	CCACCTATTGACATAGGTGG	yes
19	34117	-	gp195	TAGACGGATTTAAAATCCGTCTA	yes
20	38650	-	gp191	TAGAAGTAGGTAAACGTCCCTACTTCTA	yes
21	39504	-	gp190	GTGACTTTAAGTAGTCAC	yes
22	44524	+	gp182	ACAATTCTTAGGTGATTATAGTGATTTTATTTAGCACTATAGTCATCTATTCTATTGT	no
23	46420	+	gp179	GAGGAGTAATTACTCCTC	yes
24	48946	+	gp176	GCCTAGAATAAATCTAGGC	yes
25	52413	+	gp173	GGGATAAACTTAGGGTTTATCCC	yes
26	58075	+	gp166	GGAGTACCTGGATTAGGTACTCC	yes
27	58573	+	gp165	GACCAACTAAAAAGTTGGTC	yes
28	61075	+	gp159	GGGTGGTAGGTGATACTACCATCC	yes
29	65202	+	gp158	AAGAGTAGTATCATAGTATACTACTCTT	no
30	71144	+	gp155	GACCTATTAATTTAGGTC	yes
31	81519	+	gp148	GAGGGGTTGATTGACCCCTC	yes
32	85424	+	gp144	GACTAGGAGAAATTTCCTAGTC	yes
33	90335	+	gp141	TATACCACTTGACATTTTATATGTTAGGTGGTATA	yes
34	101128	+	gp128	TCCTACTATCTGTGCTATACTATAATAGTACAAGGTAGTAGGA	yes
35	102225	+	gp126	GAAGAGAAATAATTCTCTTC	yes
36	107425	+	gp125	ATATAGTTAACTTATGTTATACTATAT	no
37	109585	+	gp120	GAGTGCCTTAGAGCACTC	yes
38	111908	+	gp117	GCCTTCCTTGTGTTATATTATTATCGAGAATTCAATAATAAAGCATAGGGAAGGC	yes
39	113445	+	gp114	GACCAACTAAAAAGTTGGTC	yes
40	116475	+	gp109	GAGTCAAGTCTTTACTTGACTC	yes
41	119195	+	gp105	ACAGAAACTAGTTAAGTTTTTTCTACTTGCTCTAGTTTCTGT	no
42	121936	+	gp101	GTCTCCTCTTATATTATAATTGTAAGAGGGGAC	yes
43	122468	+	gp100	GAGTCAAGTTAATTCTTGACTC	yes
44	129218	+	gp080	GAAACCTATTGACATTAGGTTTC	yes
45	132814	+	gp070	TACCTGTTGACAGCCTGTTGACAGCAGGTA	yes
46	136378	+	gp064	CTCCCTATTGACAAAGGGAG	yes
47	142898	+	gp046	CAGGTGTCTATATATATATATAGACGGTTG	no
48	142954	-	gp045	TACACTGGGAATAATATCCTAGTGTA	yes
49	144705	+	gp042	TCCCTAGGATTAGATTTCTAGGGA	yes
50	144981	-	gp041	GAAAAGGGTTGACCTTTTC	yes
51	148201	+	gp033	GAGGGAATAAAATCCCTC	yes

TABLE S3 **Expression of *Staphylococcus* phage K genes.**

Phage K CDSs (products) were designated according to the blast and CDD hits. The expression cluster of each genes was deduced from the the time the gene reach its maximum and based on the heatmap clustering of phage K log2-transformed normalized counts per gene generated using DESeq2 as described in Materials and methods. Transcription unit was assigned based on the promoters localization, which control the transcription of the genes in the unit.

Accession	From	To	Locus	Strand	Feature	Protein ID	Product (CDD and blast hits)	Expression cluster	Transcription unit
NC_005880.2	486	785	CPT_phageK_gp001	+	CDS	YP_009041223.1	hypothetical protein	early	TU1
NC_005880.2	801	986	CPT_phageK_gp002	+	CDS	YP_009041224.1	putative membrane protein	early	TU1
NC_005880.2	1093	1380	CPT_phageK_gp003	+	CDS	YP_009041225.1	hypothetical protein	early	TU1
NC_005880.2	1380	1706	CPT_phageK_gp004	+	CDS	YP_009041226.1	hypothetical protein	early	TU1
NC_005880.2	1722	2015	CPT_phageK_gp005	+	CDS	YP_009041227.1	hypothetical protein	early	TU1
NC_005880.2	2019	2276	CPT_phageK_gp006	+	CDS	YP_009041228.1	hypothetical protein	early	TU1
NC_005880.2	2354	2593	CPT_phageK_gp007	+	CDS	YP_009041229.1	hypothetical protein	early	TU1
NC_005880.2	2604	2951	CPT_phageK_gp008	+	CDS	YP_009041230.1	hypothetical protein	early	TU1
NC_005880.2	3162	3500	CPT_phageK_gp009	-	CDS	YP_009041231.1	hypothetical protein	early	TU2
NC_005880.2	3811	4119	CPT_phageK_gp010	+	CDS	YP_009041232.1	hypothetical protein	early	TU3
NC_005880.2	4325	4612	CPT_phageK_gp011	+	CDS	YP_009041233.1	hypothetical protein	early	TU4
NC_005880.2	4662	4853	CPT_phageK_gp012	+	CDS	YP_009041234.1	hypothetical protein	early	TU4
NC_005880.2	5170	5658	CPT_phageK_gp013	-	CDS	YP_009041235.1	putative HNH endonuclease	middle	TU5
NC_005880.2	5826	5984	CPT_phageK_gp014	+	CDS	YP_009041236.1	putative membrane protein	early	TU6
NC_005880.2	6054	6185	CPT_phageK_gp015	+	CDS	YP_009041237.1	hypothetical protein	early	TU7
NC_005880.2	6353	6676	CPT_phageK_gp016	+	CDS	YP_009041238.1	hypothetical protein	early	TU9
NC_005880.2	6776	7012	CPT_phageK_gp017	+	CDS	YP_009041239.1	hypothetical protein	early	TU10
NC_005880.2	7092	7562	CPT_phageK_gp018	+	CDS	YP_009041240.1	hypothetical protein	early	TU11
NC_005880.2	7621	7794	CPT_phageK_gp019	+	CDS	YP_009041241.1	hypothetical protein	early	TU11
NC_005880.2	7794	8063	CPT_phageK_gp020	+	CDS	YP_009041242.1	hypothetical protein	early	TU11
NC_005880.2	8148	8369	CPT_phageK_gp021	+	CDS	YP_009041243.1	hypothetical protein	early	TU12
NC_005880.2	8666	8902	CPT_phageK_gp022	-	CDS	YP_009041244.1	hypothetical protein	middle	TU13
NC_005880.2	8902	9075	CPT_phageK_gp023	-	CDS	YP_009041245.1	hypothetical protein	middle	TU13
NC_005880.2	9082	9336	CPT_phageK_gp024	-	CDS	YP_009041246.1	hypothetical protein	middle	TU13
NC_005880.2	9527	10012	CPT_phageK_gp025	-	CDS	YP_009041247.1	putative membrane protein	middle	TU14
NC_005880.2	10005	10436	CPT_phageK_gp026	-	CDS	YP_009041248.1	hypothetical protein	middle	TU14
NC_005880.2	10450	10992	CPT_phageK_gp027	-	CDS	YP_009041249.1	nucleotidyl transferase	middle	TU14
NC_005880.2	11004	11492	CPT_phageK_gp028	-	CDS	YP_009041250.1	conserved hypothetical protein, ribA/ribD-fused, N-glycosidase YbiA-like protein	middle	TU14
NC_005880.2	11505	11903	CPT_phageK_gp029	-	CDS	YP_009041251.1	hypothetical protein	middle	TU14
NC_005880.2	11900	12607	CPT_phageK_gp030	-	CDS	YP_009041252.1	serine threonine phosphatase	middle	TU14
NC_005880.2	12707	13261	CPT_phageK_gp031	-	CDS	YP_009041253.1	hypothetical protein	middle	TU15
NC_005880.2	13277	13594	CPT_phageK_gp032	-	CDS	YP_009041254.1	hydrolase domain protein	middle	TU15
NC_005880.2	14229	14300	CPT_phageK_gt001	-	tRNA	N.A.		middle	TU16
NC_005880.2	14580	15128	CPT_phageK_gp233	-	CDS	YP_009041255.1	conserved hypothetical protein	middle	TU17
NC_005880.2	15132	15350	CPT_phageK_gp232	-	CDS	YP_009041256.1	hypothetical protein	middle	TU17
NC_005880.2	15351	15545	CPT_phageK_gp231	-	CDS	YP_009041257.1	hypothetical protein	middle	TU17
NC_005880.2	15535	16272	CPT_phageK_gp230	-	CDS	YP_009041258.1	hypothetical protein	middle	TU18
NC_005880.2	16451	16690	CPT_phageK_gp229	-	CDS	YP_009041259.1	hypothetical protein	early	TU19
NC_005880.2	16692	17081	CPT_phageK_gp228	-	CDS	YP_009041260.1	hypothetical protein	early	TU19
NC_005880.2	17180	17353	CPT_phageK_gp227	-	CDS	YP_009041261.1	hypothetical protein	late	TU20
NC_005880.2	17394	17876	CPT_phageK_gp226	-	CDS	YP_009041262.1	conserved hypothetical protein	middle	TU20
NC_005880.2	17926	18468	CPT_phageK_gp225	-	CDS	YP_009041263.1	hypothetical protein	middle	TU21
NC_005880.2	18468	19001	CPT_phageK_gp224	-	CDS	YP_009041264.1	hypothetical protein	middle	TU21
NC_005880.2	19004	19168	CPT_phageK_gp223	-	CDS	YP_009041265.1	putative membrane protein	middle	TU21
NC_005880.2	19171	19446	CPT_phageK_gp222	-	CDS	YP_009041266.1	MFS transporter-like protein	middle	TU21
NC_005880.2	19446	20291	CPT_phageK_gp221	-	CDS	YP_009041267.1	hypothetical protein	middle	TU21
NC_005880.2	20303	20887	CPT_phageK_gp220	-	CDS	YP_009041268.1	AAA family ATPase	middle	TU21
NC_005880.2	21156	21422	CPT_phageK_gp219	-	CDS	YP_009041269.1	AAA family ATPase	early	TU21
NC_005880.2	21576	21902	CPT_phageK_gp218	-	CDS	YP_009041270.1	hypothetical protein	middle	TU22
NC_005880.2	21895	22311	CPT_phageK_gp217	-	CDS	YP_009041271.1	putative nucleotide kinase, DUF3310 domain protein	middle	TU22
NC_005880.2	22445	22747	CPT_phageK_gp216	-	CDS	YP_009041272.1	MazG-like family protein Hmzg	middle	TU23
NC_005880.2	22747	22935	CPT_phageK_gp215	-	CDS	YP_009041273.1	hypothetical protein	middle	TU23
NC_005880.2	22979	23140	CPT_phageK_gp214	-	CDS	YP_009041274.1	hypothetical protein	middle	TU24
NC_005880.2	23140	25188	CPT_phageK_gp213	-	CDS	YP_009041275.1	diguanylate cyclase/phosphodiesterase	middle	TU24
NC_005880.2	25266	25529	CPT_phageK_gp212	-	CDS	YP_009041276.1	hypothetical protein	middle	TU25
NC_005880.2	25546	25719	CPT_phageK_gp211	-	CDS	YP_009041277.1	LysM peptidoglycan binding domain protein, neck connector	middle	TU25
NC_005880.2	25726	26304	CPT_phageK_gp210	-	CDS	YP_009041278.1	conserved hypothetical protein	middle	TU25
NC_005880.2	26297	26923	CPT_phageK_gp209	-	CDS	YP_009041279.1	nucleoside 2-deoxyribosyltransferase	middle	TU25
NC_005880.2	26916	27812	CPT_phageK_gp208	-	CDS	YP_009041280.1	RNA ligase	middle	TU25
NC_005880.2	27812	28036	CPT_phageK_gp207	-	CDS	YP_009041281.1	putative membrane protein	middle	TU25
NC_005880.2	28105	28845	CPT_phageK_gp206	-	CDS	YP_009041282.1	PhoH family protein	middle	TU26
NC_005880.2	28897	29511	CPT_phageK_gp205	-	CDS	YP_009041283.1	conserved hypothetical protein	middle	TU26
NC_005880.2	29527	29952	CPT_phageK_gp204	-	CDS	YP_009041284.1	ribonuclease H	middle	TU26
NC_005880.2	29942	30133	CPT_phageK_gp203	-	CDS	YP_009041285.1	hypothetical protein	middle	TU26
NC_005880.2	30156	30797	CPT_phageK_gp202	-	CDS	YP_009041286.1	hypothetical protein	middle	TU26
NC_005880.2	30787	31017	CPT_phageK_gp201	-	CDS	YP_009041287.1	HTH XRE family protein, putative transcriptional regulator	middle	TU26
NC_005880.2	31020	31247	CPT_phageK_gp200	-	CDS	YP_009041288.1	hypothetical protein	middle	TU26
NC_005880.2	31357	32049	CPT_phageK_gp199	-	CDS	YP_009041289.1	putative transglycosylase, putative cell division protein FtsN domain protein, phage tail lysozyme domain protein	middle	TU27
NC_005880.2	32236	32871	CPT_phageK_gp198	-	CDS	YP_009041290.1	hypothetical protein	middle	TU28
NC_005880.2	32938	33729	CPT_phageK_gp197	-	CDS	YP_009041291.1	prohibition family protein	middle	TU29
NC_005880.2	33729	34037	CPT_phageK_gp196	-	CDS	YP_009041292.1	putative membrane protein	middle	TU29
NC_005880.2	35716	36513	CPT_phageK_gp195	-	CDS	YP_009041293.1	endolysin	late	TU31
NC_005880.2	35050	35550	CPT_phageK_gp194	-	CDS	YP_009041294.1	HNH endonuclease	middle	TU30
NC_005880.2	36513	37016	CPT_phageK_gp193	-	CDS	YP_009041295.1	holin	late	TU31
NC_005880.2	37101	37286	CPT_phageK_gp192	-	CDS	YP_009041296.1	hypothetical protein	middle	TU32
NC_005880.2	37448	37519	CPT_phageK_gt002	-	tRNA	N.A.		middle	TU32
NC_005880.2	37526	37598	CPT_phageK_gt003	-	tRNA	N.A.		middle	TU32
NC_005880.2	37605	37678	CPT_phageK_gt004	-	tRNA	N.A.		middle	TU32
NC_005880.2	38833	39051	CPT_phageK_gp191	-	CDS	YP_009041297.1	putative DNA binding protein, ribosome associated inhibitor raiA	early	TU33
NC_005880.2	39529	39738	CPT_phageK_gp190	-	CDS	YP_009041298.1	hypothetical protein	late	TU34
NC_005880.2	39751	40083	CPT_phageK_gp189	-	CDS	YP_009041299.1	conserved hypothetical protein	late	TU34

TABLE S3 continued

Accession	From	To	Locus	Strand	Feature	Protein ID	Product (CDD and blast hits)	Expression cluster	Transcription unit
NC_005880.2	40096	40422	CPT_phageK_gp188	-	CDS	YP_009041300.1	putative membrane protein	late	TU34
NC_005880.2	40455	40721	CPT_phageK_gp187	-	CDS	YP_009041301.1	hypothetical protein	late	TU34
NC_005880.2	40862	41248	CPT_phageK_gp186	+	CDS	YP_009041302.1	putative membrane protein	late	TU35
NC_005880.2	41226	41504	CPT_phageK_gp185	+	CDS	YP_009041303.1	hypothetical protein	late	TU35
NC_005880.2	41501	41911	CPT_phageK_gp184	+	CDS	YP_009041304.1	hypothetical protein	late	TU35
NC_005880.2	41926	43743	CPT_phageK_gp183	+	CDS	YP_009041305.1	terminase large subunit	late	TU35
NC_005880.2	43736	44557	CPT_phageK_gp182	+	CDS	YP_009041306.1	hypothetical protein	middle	TU36
NC_005880.2	44544	44717	CPT_phageK_gp181	+	CDS	YP_009041307.1	hypothetical protein	middle	TU37
NC_005880.2	44714	45193	CPT_phageK_gp180	+	CDS	YP_009041308.1	hypothetical protein	middle	TU37
NC_005880.2	45235	46428	CPT_phageK_gp179	+	CDS	YP_009041309.1	hypothetical protein	middle	TU37
NC_005880.2	46513	46854	CPT_phageK_gp178	+	CDS	YP_009041310.1	conserved hypothetical protein	late	TU38
NC_005880.2	46872	47243	CPT_phageK_gp177	+	CDS	YP_009041311.1	hypothetical protein	late	TU38
NC_005880.2	47247	48938	CPT_phageK_gp176	+	CDS	YP_009041312.1	portal protein	late	TU38
NC_005880.2	49132	49905	CPT_phageK_gp175	+	CDS	YP_009041313.1	prohead protease	late	TU39
NC_005880.2	49924	50874	CPT_phageK_gp174	+	CDS	YP_009041314.1	K+-dependent Na+/Ca+ exchanger	late	TU39
NC_005880.2	50990	52381	CPT_phageK_gp173	+	CDS	YP_009041315.1	major capsid protein	late	TU40
NC_005880.2	52473	52769	CPT_phageK_gp172	+	CDS	YP_009041316.1	connector related protein	late	TU41
NC_005880.2	52782	53690	CPT_phageK_gp171	+	CDS	YP_009041317.1	connector related protein	late	TU41
NC_005880.2	53704	54582	CPT_phageK_gp170	+	CDS	YP_009041318.1	connector related protein	late	TU41
NC_005880.2	54582	55202	CPT_phageK_gp169	+	CDS	YP_009041319.1	DNA anti-recombination protein RmuC	late	TU41
NC_005880.2	55221	56057	CPT_phageK_gp168	+	CDS	YP_009041320.1	tail completion protein	late	TU42
NC_005880.2	56059	56274	CPT_phageK_gp167	+	CDS	YP_009041321.1	hypothetical protein	late	TU42
NC_005880.2	56301	58064	CPT_phageK_gp166	+	CDS	YP_009041322.1	tail sheath protein	late	TU43
NC_005880.2	58137	58565	CPT_phageK_gp165	+	CDS	YP_009041323.1	tail tube protein	late	TU43
NC_005880.2	58662	58802	CPT_phageK_gp164	+	CDS	YP_009041324.1	hypothetical protein	middle	TU44
NC_005880.2	58845	59303	CPT_phageK_gp163	+	CDS	YP_009041325.1	conserved hypothetical protein	early	TU44
NC_005880.2	59316	59510	CPT_phageK_gp162	+	CDS	YP_009041326.1	putative membrane protein	early	TU44
NC_005880.2	59592	59903	CPT_phageK_gp161	+	CDS	YP_009041327.1	hypothetical protein	middle	TU45
NC_005880.2	60035	60493	CPT_phageK_gp160	+	CDS	YP_009041328.1	tail tape measure protein chaperone	late	TU46
NC_005880.2	60537	61073	CPT_phageK_gp159	+	CDS	YP_009041329.1	tail morphogenetic protein chaperone	late	TU46
NC_005880.2	61129	65184	CPT_phageK_gp158	+	CDS	YP_009041330.1	tail tape measure protein	late	TU47
NC_005880.2	65263	67689	CPT_phageK_gp157	+	CDS	YP_009041331.1	baseplate hub	middle	TU48
NC_005880.2	67703	68590	CPT_phageK_gp156	+	CDS	YP_009041332.1	putative peptidoglycan hydrolase	middle	TU48
NC_005880.2	68590	71136	CPT_phageK_gp155	+	CDS	YP_009041333.1	tail central spike	middle	TU48
NC_005880.2	71243	72034	CPT_phageK_gp154	+	CDS	YP_009041334.1	tail tube initiator	middle	TU49
NC_005880.2	72034	72558	CPT_phageK_gp153	+	CDS	YP_009041335.1	putative baseplate component	middle	TU49
NC_005880.2	72558	73262	CPT_phageK_gp152	+	CDS	YP_009041336.1	tail sheath initiator	middle	TU49
NC_005880.2	73277	74323	CPT_phageK_gp151	+	CDS	YP_009041337.1	baseplate wedge protein	middle	TU49
NC_005880.2	74344	77403	CPT_phageK_gp150	+	CDS	YP_009041338.1	baseplate arm protein	middle	TU49
NC_005880.2	77514	78035	CPT_phageK_gp149	+	CDS	YP_009041339.1	baseplate arm protein	middle	TU50
NC_005880.2	78056	81514	CPT_phageK_gp148	+	CDS	YP_009041340.1	tripod protein	middle	TU50
NC_005880.2	81563	81721	CPT_phageK_gp147	+	CDS	YP_009041341.1	hypothetical protein	middle	TU51
NC_005880.2	81722	83644	CPT_phageK_gp146	+	CDS	YP_009041342.1	receptor binding protein 1	middle	TU51
NC_005880.2	83667	84041	CPT_phageK_gp145	+	CDS	YP_009041343.1	putative baseplate component	middle	TU51
NC_005880.2	84048	85424	CPT_phageK_gp144	+	CDS	YP_009041344.1	receptor binding protein 2	middle	TU51
NC_005880.2	85516	87264	CPT_phageK_gp143	+	CDS	YP_009041345.1	UV repair and recombination protein UvsW	middle	TU52
NC_005880.2	87276	88889	CPT_phageK_gp142	+	CDS	YP_009041346.1	putative Rep protein	middle	TU52
NC_005880.2	88882	90324	CPT_phageK_gp141	+	CDS	YP_009041347.1	DNA primase helicase	middle	TU52
NC_005880.2	90403	91440	CPT_phageK_gp140	+	CDS	YP_009041348.1	recombination exonuclease	middle	TU53
NC_005880.2	91440	91817	CPT_phageK_gp139	+	CDS	YP_009041349.1	hypothetical protein	middle	TU53
NC_005880.2	91817	93736	CPT_phageK_gp138	+	CDS	YP_009041350.1	recombination endonuclease	middle	TU53
NC_005880.2	93736	94332	CPT_phageK_gp137	+	CDS	YP_009041351.1	antisigma factor Asf	middle	TU53
NC_005880.2	94347	95414	CPT_phageK_gp136	+	CDS	YP_009041352.1	DNA primase	middle	TU54
NC_005880.2	95481	95819	CPT_phageK_gp135	+	CDS	YP_009041353.1	hypothetical protein	middle	TU55
NC_005880.2	95819	96271	CPT_phageK_gp134	+	CDS	YP_009041354.1	conserved hypothetical protein	middle	TU55
NC_005880.2	96258	96866	CPT_phageK_gp133	+	CDS	YP_009041355.1	Holliday junction resolvase	middle	TU55
NC_005880.2	96883	97275	CPT_phageK_gp132	+	CDS	YP_009041356.1	ribonucleotide reductase	middle	TU55
NC_005880.2	97290	99404	CPT_phageK_gp131	+	CDS	YP_009041357.1	ribonucleoside-diphosphate reductase large subunit	middle	TU55
NC_005880.2	99418	100467	CPT_phageK_gp130	+	CDS	YP_009041358.1	ribonucleoside-diphosphate reductase small subunit	middle	TU55
NC_005880.2	100485	100814	CPT_phageK_gp129	+	CDS	YP_009041359.1	hypothetical protein	middle	TU55
NC_005880.2	100798	101118	CPT_phageK_gp128	+	CDS	YP_009041360.1	putative thioredoxin	middle	TU55
NC_005880.2	101325	101921	CPT_phageK_gp127	+	CDS	YP_009041361.1	hypothetical protein	middle	TU56
NC_005880.2	101931	102236	CPT_phageK_gp126	+	CDS	YP_009041362.1	putative DNA binding protein	middle	TU56
NC_005880.2	102312	103181	CPT_phageK_gp125	+	CDS	YP_009041363.1	DNA polymerase I	middle	TU57
NC_005880.2	103392	103862	CPT_phageK_gp124	+	CDS	YP_009041364.1	hypothetical protein	middle	TU57
NC_005880.2	105507	106316	CPT_phageK_gp123	+	CDS	YP_009041365.1	HNH endonuclease	middle	TU57
NC_005880.2	107479	107721	CPT_phageK_gp122	+	CDS	YP_009041366.1	hypothetical protein	middle	TU58
NC_005880.2	107738	108220	CPT_phageK_gp121	+	CDS	YP_009041367.1	putative chellatase	middle	TU58
NC_005880.2	108307	109578	CPT_phageK_gp120	+	CDS	YP_009041368.1	hypothetical protein	middle	TU59
NC_005880.2	109638	110894	CPT_phageK_gp119	+	CDS	YP_009041369.1	putative RecA-like DNA recombinase	middle	TU60
NC_005880.2	110898	111251	CPT_phageK_gp118	+	CDS	YP_009041370.1	hydrolase domain protein	middle	TU60
NC_005880.2	111238	111900	CPT_phageK_gp117	+	CDS	YP_009041371.1	RNA polymerase sigma factor	middle	TU60
NC_005880.2	112028	112660	CPT_phageK_gp116	+	CDS	YP_009041372.1	head decoration protein	late	TU61
NC_005880.2	112683	113195	CPT_phageK_gp115	+	CDS	YP_009041373.1	head decoration protein	late	TU61
NC_005880.2	113210	113437	CPT_phageK_gp114	+	CDS	YP_009041374.1	head decoration protein	late	TU61
NC_005880.2	113533	113793	CPT_phageK_gp113	+	CDS	YP_009041375.1	hypothetical protein	middle	TU62
NC_005880.2	113797	114552	CPT_phageK_gp112	+	CDS	YP_009041376.1	hypothetical protein	middle	TU62
NC_005880.2	114545	115795	CPT_phageK_gp111	+	CDS	YP_009041377.1	putative DNA repair exonuclease	middle	TU62
NC_005880.2	115809	116177	CPT_phageK_gp110	+	CDS	YP_009041378.1	putative membrane protein	middle	TU62
NC_005880.2	116164	116475	CPT_phageK_gp109	+	CDS	YP_009041379.1	hypothetical protein	middle	TU62
NC_005880.2	116539	117075	CPT_phageK_gp108	+	CDS	YP_009041380.1	hypothetical protein	middle	TU63
NC_005880.2	117068	117835	CPT_phageK_gp107	+	CDS	YP_009041381.1	hypothetical protein	middle	TU63
NC_005880.2	117813	118259	CPT_phageK_gp106	+	CDS	YP_009041382.1	hypothetical protein	middle	TU63
NC_005880.2	118259	119122	CPT_phageK_gp105	+	CDS	YP_009041383.1	hypothetical protein	middle	TU63
NC_005880.2	119494	120225	CPT_phageK_gp104	+	CDS	YP_009041384.1	hypothetical protein	middle	TU64
NC_005880.2	120243	120701	CPT_phageK_gp103	+	CDS	YP_009041385.1	hypothetical protein	middle	TU64
NC_005880.2	120766	121209	CPT_phageK_gp102	+	CDS	YP_009041386.1	hypothetical protein	middle	TU65
NC_005880.2	121226	121930	CPT_phageK_gp101	+	CDS	YP_009041387.1	hypothetical protein	middle	TU65
NC_005880.2	121992	122390	CPT_phageK_gp100	+	CDS	YP_009041388.1	putative membrane protein	middle	TU66
NC_005880.2	122537	122779	CPT_phageK_gp099	+	CDS	YP_009041389.1	hypothetical protein	middle	TU67
NC_005880.2	122784	123341	CPT_phageK_gp098	+	CDS	YP_009041390.1	hypothetical protein	middle	TU67
NC_005880.2	123377	123553	CPT_phageK_gp097	+	CDS	YP_009041391.1	sliding clamp inhibitor Sci	middle	TU67
NC_005880.2	123543	124076	CPT_phageK_gp096	+	CDS	YP_009041392.1	putative membrane protein	middle	TU67

TABLE S3 continued

Accession	From	To	Locus	Strand	Feature	Protein ID	Product (CDD and blast hits)	Expression cluster	Transcription unit
NC_005880.2	124091	124339	CPT_phageK_gp095	+	CDS	YP_009041393.1	hypothetical protein	middle	TU67
NC_005880.2	124351	124527	CPT_phageK_gp094	+	CDS	YP_009041394.1	hypothetical protein	middle	TU67
NC_005880.2	124520	124816	CPT_phageK_gp093	+	CDS	YP_009041395.1	hypothetical protein	early	TU67
NC_005880.2	124864	125046	CPT_phageK_gp092	+	CDS	YP_009041396.1	putative membrane protein	early	TU67
NC_005880.2	125059	125427	CPT_phageK_gp091	+	CDS	YP_009041397.1	hypothetical protein	early	TU67
NC_005880.2	125440	125787	CPT_phageK_gp090	+	CDS	YP_009041398.1	hypothetical protein	early	TU67
NC_005880.2	125793	126065	CPT_phageK_gp089	+	CDS	YP_009041399.1	putative membrane protein	early	TU67
NC_005880.2	126135	126440	CPT_phageK_gp088	+	CDS	YP_009041400.1	hypothetical protein	early	TU67
NC_005880.2	126455	126805	CPT_phageK_gp087	+	CDS	YP_009041401.1	hypothetical protein	early	TU67
NC_005880.2	126805	127407	CPT_phageK_gp086	+	CDS	YP_009041402.1	putative endonuclease	middle	TU67
NC_005880.2	127421	127600	CPT_phageK_gp085	+	CDS	YP_009041403.1	hypothetical protein	middle	TU67
NC_005880.2	127827	128228	CPT_phageK_gp084	+	CDS	YP_009041404.1	putative membrane protein	early	TU67
NC_005880.2	128230	128523	CPT_phageK_gp083	+	CDS	YP_009041405.1	hypothetical protein, N,N-dimethylguanosine tRNA methyltransferase hit	middle	TU67
NC_005880.2	128540	128827	CPT_phageK_gp082	+	CDS	YP_009041406.1	putative membrane protein	middle	TU67
NC_005880.2	128838	128954	CPT_phageK_gp081	+	CDS	YP_009041407.1	hypothetical protein, alanyl-tRNA synthetase hit	middle	TU67
NC_005880.2	128944	129207	CPT_phageK_gp080	+	CDS	YP_009041408.1	hypothetical protein	middle	TU67
NC_005880.2	129284	129589	CPT_phageK_gp079	+	CDS	YP_009041409.1	hypothetical protein	early	TU68
NC_005880.2	129589	129960	CPT_phageK_gp078	+	CDS	YP_009041410.1	hypothetical protein	early	TU68
NC_005880.2	129998	130234	CPT_phageK_gp077	+	CDS	YP_009041411.1	hypothetical protein	early	TU68
NC_005880.2	130231	130758	CPT_phageK_gp076	+	CDS	YP_009041412.1	putative DNA binding protein, putative metallophosphatase	early	TU68
NC_005880.2	130739	131059	CPT_phageK_gp075	+	CDS	YP_009041413.1	hypothetical protein	early	TU68
NC_005880.2	131059	131289	CPT_phageK_gp074	+	CDS	YP_009041414.1	putative membrane protein	early	TU68
NC_005880.2	131342	131521	CPT_phageK_gp073	+	CDS	YP_009041415.1	putative membrane protein	early	TU68
NC_005880.2	131536	131799	CPT_phageK_gp072	+	CDS	YP_009041416.1	hypothetical protein	middle	TU68
NC_005880.2	131802	132119	CPT_phageK_gp071	+	CDS	YP_009041417.1	hypothetical protein	middle	TU68
NC_005880.2	132120	132800	CPT_phageK_gp070	+	CDS	YP_009041418.1	hypothetical protein	middle	TU68
NC_005880.2	132889	133047	CPT_phageK_gp069	+	CDS	YP_009041419.1	putative membrane protein	early	TU69
NC_005880.2	133082	133282	CPT_phageK_gp068	+	CDS	YP_009041420.1	hypothetical protein	early	TU69
NC_005880.2	133283	133573	CPT_phageK_gp067	+	CDS	YP_009041421.1	putative membrane protein	middle	TU69
NC_005880.2	133665	133973	CPT_phageK_gp066	+	CDS	YP_009041422.1	hypothetical protein	early	TU69
NC_005880.2	133970	134878	CPT_phageK_gp065	+	CDS	YP_009041423.1	putative ribose-phosphate pyrophosphokinase	middle	TU69
NC_005880.2	134896	136365	CPT_phageK_gp064	+	CDS	YP_009041424.1	putative nicotinamide phosphoribosyltransferase	middle	TU69
NC_005880.2	136444	136689	CPT_phageK_gp063	+	CDS	YP_009041425.1	hypothetical protein	early	TU70
NC_005880.2	136706	137101	CPT_phageK_gp062	+	CDS	YP_009041426.1	hypothetical protein	early	TU70
NC_005880.2	137098	137349	CPT_phageK_gp061	+	CDS	YP_009041427.1	hypothetical protein	early	TU70
NC_005880.2	137414	137710	CPT_phageK_gp060	+	CDS	YP_009041428.1	hypothetical protein	early	TU71
NC_005880.2	137714	138025	CPT_phageK_gp059	+	CDS	YP_009041429.1	putative CamS-like protein	early	TU71
NC_005880.2	138031	138330	CPT_phageK_gp058	+	CDS	YP_009041430.1	hypothetical protein	early	TU71
NC_005880.2	138409	138636	CPT_phageK_gp057	+	CDS	YP_009041431.1	hypothetical protein	early	TU71
NC_005880.2	138664	138858	CPT_phageK_gp056	+	CDS	YP_009041432.1	hypothetical protein	early	TU71
NC_005880.2	138877	139230	CPT_phageK_gp055	+	CDS	YP_009041433.1	hypothetical protein	early	TU71
NC_005880.2	139249	139635	CPT_phageK_gp054	+	CDS	YP_009041434.1	hypothetical protein	early	TU71
NC_005880.2	140317	140616	CPT_phageK_gp053	+	CDS	YP_009041435.1	hypothetical protein	early	TU72
NC_005880.2	140632	140817	CPT_phageK_gp052	+	CDS	YP_009041436.1	putative membrane protein	early	TU71
NC_005880.2	140924	141211	CPT_phageK_gp051	+	CDS	YP_009041437.1	hypothetical protein	early	TU71
NC_005880.2	141211	141537	CPT_phageK_gp050	+	CDS	YP_009041438.1	hypothetical protein	early	TU71
NC_005880.2	141553	141846	CPT_phageK_gp049	+	CDS	YP_009041439.1	hypothetical protein	early	TU71
NC_005880.2	141850	142107	CPT_phageK_gp048	+	CDS	YP_009041440.1	hypothetical protein	early	TU71
NC_005880.2	142185	142424	CPT_phageK_gp047	+	CDS	YP_009041441.1	hypothetical protein	early	TU71
NC_005880.2	142435	142782	CPT_phageK_gp046	+	CDS	YP_009041442.1	hypothetical protein	early	TU71
NC_005880.2	142993	143331	CPT_phageK_gp045	-	CDS	YP_009041443.1	hypothetical protein	early	TU73
NC_005880.2	143642	143950	CPT_phageK_gp044	+	CDS	YP_009041444.1	hypothetical protein	early	TU74
NC_005880.2	144156	144443	CPT_phageK_gp043	+	CDS	YP_009041445.1	hypothetical protein	early	TU75
NC_005880.2	144493	144684	CPT_phageK_gp042	+	CDS	YP_009041446.1	hypothetical protein	early	TU75
NC_005880.2	145001	145489	CPT_phageK_gp041	-	CDS	YP_009041447.1	putative HNH endonuclease	middle	TU76
NC_005880.2	145657	145815	CPT_phageK_gp040	+	CDS	YP_009041448.1	putative membrane protein	early	TU77
NC_005880.2	145885	146016	CPT_phageK_gp039	+	CDS	YP_009041449.1	hypothetical protein	early	TU78
NC_005880.2	146184	146507	CPT_phageK_gp038	+	CDS	YP_009041450.1	hypothetical protein	early	TU80
NC_005880.2	146607	146843	CPT_phageK_gp037	+	CDS	YP_009041451.1	hypothetical protein	early	TU81
NC_005880.2	146923	147393	CPT_phageK_gp036	+	CDS	YP_009041452.1	hypothetical protein	early	TU82
NC_005880.2	147452	147625	CPT_phageK_gp035	+	CDS	YP_009041453.1	hypothetical protein	early	TU82
NC_005880.2	147625	147894	CPT_phageK_gp034	+	CDS	YP_009041454.1	hypothetical protein	early	TU82
NC_005880.2	147979	148200	CPT_phageK_gp033	+	CDS	YP_009041455.1	hypothetical protein	early	TU83

Legend

N.A. not available

TABLE S4A Significantly differentially expressed genes in *Staphylococcus aureus* Newman

Genomic sequence of *S. aureus* Newman deposited in the RefSeq database (Accession number: NC_009641.1) was used for the differential expression analysis with the DESeq2 R package. Genes with log2-fold changes (L2FC) ± 0.58 with $p < 0.05$ corresponding to 1.5-fold upregulation or downregulation are listed below.

	Time 2 min			Time 5 min			Time 10 min			Time 20 min			Time 30 min		
#	gene ID	log2 Fold Change	p adj. value	gene ID	log2 Fold Change	p adj. value	gene ID	log2 Fold Change	p adj. value	gene ID	log2 Fold Change	p adj. value	gene ID	log2 Fold Change	p adj. value
1	NWMN_RS02230	-1.6345	0.0190	NWMN_RS02395	-2.5374	0.0000	NWMN_RS02395	-2.9816	0.0000	NWMN_RS14920	-2.9323	0.0000	NWMN_RS06860	-3.8450	0.0184
2	NWMN_RS02395	-1.3154	0.0073	NWMN_RS12170	-2.4248	0.0330	NWMN_RS14920	-2.8132	0.0000	NWMN_RS04305	-2.4590	0.0000	NWMN_RS14920	-2.7799	0.0000
3	NWMN_RS13950	-1.2513	0.0172	NWMN_RS14920	-1.9239	0.0008	NWMN_RS04305	-2.3435	0.0000	NWMN_RS09960	-2.2457	0.0007	NWMN_RS04305	-2.4093	0.0000
4	NWMN_RS01370	-1.2228	0.0087	NWMN_RS02230	-1.7914	0.0055	NWMN_RS09960	-2.1714	0.0007	NWMN_RS09940	-2.2396	0.0000	NWMN_RS12020	-2.1469	0.0000
5	NWMN_RS05335	-1.1253	0.0075	NWMN_RS07400	-1.7597	0.0000	NWMN_RS12020	-2.1131	0.0000	NWMN_RS09875	-2.1542	0.0000	NWMN_RS01105	-1.9385	0.0000
6	NWMN_RS12020	-1.1225	0.0037	NWMN_RS01930	-1.7378	0.0000	NWMN_RS01105	-2.1117	0.0000	NWMN_RS01105	-2.1540	0.0000	NWMN_RS11205	-1.9181	0.0000
7	NWMN_RS01105	-1.0712	0.0223	NWMN_RS12020	-1.6955	0.0000	NWMN_RS09945	-1.9992	0.0002	NWMN_RS12020	-2.1318	0.0000	NWMN_RS02395	-1.9071	0.0000
8	NWMN_RS08575	-1.0077	0.0367	NWMN_RS04780	-1.6123	0.0014	NWMN_RS08715	-1.9775	0.0217	NWMN_RS11930	-2.1266	0.0001	NWMN_RS09875	-1.8634	0.0000
9	NWMN_RS07400	-1.0058	0.0167	NWMN_RS04305	-1.6007	0.0028	NWMN_RS09940	-1.9065	0.0003	NWMN_RS02395	-2.1241	0.0000	NWMN_RS05530	-1.8049	0.0000
10	NWMN_RS05340	-0.9989	0.0017	NWMN_RS09390	-1.5936	0.0027	NWMN_RS09935	-1.8274	0.0002	NWMN_RS09970	-2.0767	0.0000	NWMN_RS14390	-1.7674	0.0000
11	NWMN_RS13850	-0.9790	0.0157	NWMN_RS06270	-1.5881	0.0060	NWMN_RS01930	-1.8042	0.0000	NWMN_RS09975	-2.0092	0.0452	NWMN_RS02770	-1.7670	0.0003
12	NWMN_RS07065	-0.9438	0.0158	NWMN_RS01925	-1.5585	0.0096	NWMN_RS09975	-1.7866	0.0446	NWMN_RS09885	-1.9586	0.0002	NWMN_RS13125	-1.7442	0.0018
13	NWMN_RS12595	-0.9261	0.0017	NWMN_RS13770	-1.5173	0.0021	NWMN_RS09955	-1.7192	0.0075	NWMN_RS02770	-1.9575	0.0000	NWMN_RS01920	-1.7279	0.0001
14	NWMN_RS12795	-0.9083	0.0078	NWMN_RS11990	-1.4896	0.0005	NWMN_RS11930	-1.7070	0.0019	NWMN_RS09980	-1.9500	0.0000	NWMN_RS06270	-1.7120	0.0021
15	NWMN_RS13445	-0.8975	0.0183	NWMN_RS09825	-1.4753	0.0001	NWMN_RS09885	-1.6804	0.0012	NWMN_RS09890	-1.9055	0.0000	NWMN_RS12870	-1.6798	0.0419
16	NWMN_RS04660	-0.8957	0.0088	NWMN_RS05150	-1.4692	0.0001	NWMN_RS01920	-1.6454	0.0001	NWMN_RS09985	-1.9044	0.0057	NWMN_RS07210	-1.6730	0.0006
17	NWMN_RS01930	-0.8937	0.0421	NWMN_RS09465	-1.4629	0.0140	NWMN_RS01925	-1.5987	0.0100	NWMN_RS09935	-1.9013	0.0001	NWMN_RS12830	-1.6639	0.0000
18	NWMN_RS04335	-0.8542	0.0006	NWMN_RS01920	-1.4613	0.0007	NWMN_RS09985	-1.5880	0.0147	NWMN_RS09945	-1.8958	0.0007	NWMN_RS14350	-1.6418	0.0004
19	NWMN_RS02190	-0.8294	0.0075	NWMN_RS13010	-1.4402	0.0004	NWMN_RS09890	-1.5618	0.0002	NWMN_RS09990	-1.8728	0.0001	NWMN_RS11500	-1.6349	0.0000
20	NWMN_RS04320	-0.7960	0.0395	NWMN_RS13760	-1.4298	0.0445	NWMN_RS11945	-1.5532	0.0243	NWMN_RS13125	-1.8462	0.0007	NWMN_RS09970	-1.6203	0.0007
21	NWMN_RS09745	-0.7818	0.0249	NWMN_RS04320	-1.4289	0.0000	NWMN_RS09965	-1.5510	0.0004	NWMN_RS01925	-1.7683	0.0082	NWMN_RS11575	-1.6183	0.0013
22	NWMN_RS07405	-0.7581	0.0179	NWMN_RS13850	-1.4256	0.0001	NWMN_RS09995	-1.5486	0.0138	NWMN_RS01920	-1.6995	0.0001	NWMN_RS06890	-1.6067	0.0347
23	NWMN_RS05345	-0.7580	0.0005	NWMN_RS09395	-1.4150	0.0052	NWMN_RS09990	-1.5354	0.0011	NWMN_RS01930	-1.6060	0.0001	NWMN_RS01370	-1.6025	0.0002
24	NWMN_RS04875	-0.7488	0.0071	NWMN_RS01105	-1.3542	0.0012	NWMN_RS09970	-1.5313	0.0014	NWMN_RS09920	-1.5989	0.0016	NWMN_RS11990	-1.6018	0.0003
25	NWMN_RS01430	-0.7468	0.0462	NWMN_RS15395	-1.3231	0.0462	NWMN_RS05030	-1.5308	0.0000	NWMN_RS06890	-1.5945	0.0240	NWMN_RS09980	-1.5971	0.0003
26	NWMN_RS03715	-0.7410	0.0111	NWMN_RS07925	-1.3197	0.0036	NWMN_RS09390	-1.5068	0.0044	NWMN_RS06270	-1.5801	0.0047	NWMN_RS14715	-1.5970	0.0002
27	NWMN_RS03705	-0.7398	0.0157	NWMN_RS09495	-1.3127	0.0030	NWMN_RS04320	-1.5047	0.0000	NWMN_RS09955	-1.5554	0.0180	NWMN_RS14050	-1.5784	0.0420
28	NWMN_RS05480	-0.7222	0.0240	NWMN_RS05340	-1.3123	0.0000	NWMN_RS09395	-1.4859	0.0052	NWMN_RS09965	-1.5497	0.0005	NWMN_RS14910	-1.5679	0.0032
29	NWMN_RS03560	-0.7179	0.0363	NWMN_RS07290	-1.2976	0.0204	NWMN_RS03765	-1.4356	0.0000	NWMN_RS12800	-1.5487	0.0000	NWMN_RS11930	-1.5427	0.0063
30	NWMN_RS00120	-0.6946	0.0402	NWMN_RS08595	-1.2857	0.0363	NWMN_RS13125	-1.4260	0.0081	NWMN_RS00120	-1.5343	0.0000	NWMN_RS01930	-1.5272	0.0003
31	NWMN_RS04705	-0.6846	0.0360	NWMN_RS09740	-1.2724	0.0002	NWMN_RS09925	-1.4067	0.0020	NWMN_RS12975	-1.5330	0.0001	NWMN_RS06030	-1.5174	0.0000
32	NWMN_RS12800	-0.6802	0.0451	NWMN_RS03350	-1.2612	0.0006	NWMN_RS09950	-1.3799	0.0013	NWMN_RS14350	-1.5245	0.0009	NWMN_RS12775	-1.5052	0.0004
33	NWMN_RS08745	-0.6635	0.0388	NWMN_RS13130	-1.2529	0.0001	NWMN_RS06270	-1.3711	0.0166	NWMN_RS02825	-1.5204	0.0003	NWMN_RS02825	-1.5003	0.0004
34	NWMN_RS05370	-0.6399	0.0337	NWMN_RS05030	-1.2464	0.0002	NWMN_RS11840	-1.3600	0.0000	NWMN_RS09395	-1.5190	0.0083	NWMN_RS09390	-1.4949	0.0066
35	NWMN_RS04580	-0.6372	0.0411	NWMN_RS03765	-1.2386	0.0003	NWMN_RS11380	-1.3475	0.0000	NWMN_RS11205	-1.5121	0.0000	NWMN_RS06990	-1.4900	0.0006
36	NWMN_RS03710	-0.6302	0.0249	NWMN_RS05035	-1.2247	0.0005	NWMN_RS03755	-1.3439	0.0019	NWMN_RS01285	-1.5084	0.0084	NWMN_RS02760	-1.4767	0.0004
37	NWMN_RS03815	-0.5995	0.0046	NWMN_RS04875	-1.2109	0.0000	NWMN_RS12795	-1.3437	0.0000	NWMN_RS06790	-1.4976	0.0019	NWMN_RS06790	-1.4737	0.0026
38	NWMN_RS09845	0.7309	0.0017	NWMN_RS01370	-1.2008	0.0077	NWMN_RS09375	-1.3175	0.0002	NWMN_RS09925	-1.4844	0.0012	NWMN_RS15690	-1.4693	0.0002
39	NWMN_RS06200	0.7846	0.0289	NWMN_RS05480	-1.1948	0.0000	NWMN_RS07430	-1.3062	0.0005	NWMN_RS11945	-1.4844	0.0335	NWMN_RS09985	-1.4469	0.0403
40	NWMN_RS09475	0.7959	0.0292	NWMN_RS04050	-1.1945	0.0001	NWMN_RS09920	-1.2972	0.0080	NWMN_RS12830	-1.4486	0.0002	NWMN_RS06145	-1.4426	0.0000
41	NWMN_RS03035	0.9902	0.0262	NWMN_RS09400	-1.1937	0.0499	NWMN_RS13130	-1.2891	0.0001	NWMN_RS08915	-1.4360	0.0000	NWMN_RS12975	-1.4249	0.0002
42	NWMN_RS13190	1.1431	0.0289	NWMN_RS13445	-1.1907	0.0005	NWMN_RS01370	-1.2864	0.0030	NWMN_RS12475	-1.4313	0.0000	NWMN_RS08915	-1.4185	0.0000
43	NWMN_RS13205	1.5124	0.0186	NWMN_RS09745	-1.1547	0.0001	NWMN_RS13850	-1.2784	0.0005	NWMN_RS11840	-1.4238	0.0000	NWMN_RS11840	-1.4123	0.0000
44	NWMN_RS06310	1.5714	0.0172	NWMN_RS06845	-1.1473	0.0208	NWMN_RS12830	-1.2684	0.0007	NWMN_RS01115	-1.4150	0.0001	NWMN_RS01285	-1.4064	0.0210
45	NWMN_RS13210	1.6178	0.0026	NWMN_RS04710	-1.1426	0.0001	NWMN_RS09815	-1.2563	0.0004	NWMN_RS02780	-1.4053	0.0134	NWMN_RS01955	-1.3887	0.0002
46	NWMN_RS00865	1.7093	0.0167	NWMN_RS02445	-1.1384	0.0005	NWMN_RS04335	-1.2316	0.0000	NWMN_RS12775	-1.3967	0.0010	NWMN_RS04335	-1.3691	0.0000
47	NWMN_RS06305	1.7289	0.0142	NWMN_RS05335	-1.1180	0.0060	NWMN_RS08915	-1.2283	0.0000	NWMN_RS09390	-1.3933	0.0096	NWMN_RS12790	-1.3687	0.0000
48	NWMN_RS06295	1.8145	0.0280	NWMN_RS03610	-1.1150	0.0002	NWMN_RS01115	-1.2223	0.0007	NWMN_RS02760	-1.3850	0.0008	NWMN_RS14295	-1.3663	0.0002
49	NWMN_RS06300	1.9113	0.0111	NWMN_RS03535	-1.1145	0.0002	NWMN_RS03535	-1.2179	0.0000	NWMN_RS09435	-1.3823	0.0023	NWMN_RS12895	-1.3660	0.0007
50				NWMN_RS04660	-1.1081	0.0004	NWMN_RS05035	-1.2118	0.0005	NWMN_RS03920	-1.3731	0.0000	NWMN_RS12795	-1.3644	0.0002
51				NWMN_RS03750	-1.1071	0.0265	NWMN_RS09825	-1.2107	0.0013	NWMN_RS11940	-1.3730	0.0070	NWMN_RS05030	-1.3618	0.0001
52				NWMN_RS12595	-1.1021	0.0001	NWMN_RS03600	-1.2046	0.0004	NWMN_RS09825	-1.3679	0.0003	NWMN_RS05035	-1.3570	0.0001

TABLE S4A continued

	Time 2 min			Time 5 min			Time 10 min			Time 20 min			Time 30 min		
#	gene ID	log2 Fold Change	p adj. value	gene ID	log2 Fold Change	p adj. value	gene ID	log2 Fold Change	p adj. value	gene ID	log2 Fold Change	p adj. value	gene ID	log2 Fold Change	p adj. value
53				NWMN_RS03010	-1.0988	0.0371	NWMN_RS11540	-1.2042	0.0004	NWMN_RS01955	-1.3393	0.0001	NWMN_RS09885	-1.3519	0.0147
54				NWMN_RS09530	-1.0960	0.0055	NWMN_RS12775	-1.1879	0.0060	NWMN_RS15770	-1.3367	0.0181	NWMN_RS13845	-1.3460	0.0027
55				NWMN_RS03095	-1.0953	0.0077	NWMN_RS11940	-1.1866	0.0204	NWMN_RS13130	-1.3360	0.0000	NWMN_RS03250	-1.3392	0.0436
56				NWMN_RS06030	-1.0917	0.0021	NWMN_RS09745	-1.1797	0.0001	NWMN_RS12970	-1.3211	0.0025	NWMN_RS05360	-1.3086	0.0000
57				NWMN_RS09375	-1.0862	0.0021	NWMN_RS02445	-1.1781	0.0004	NWMN_RS01370	-1.3157	0.0024	NWMN_RS09990	-1.3069	0.0090
58				NWMN_RS11380	-1.0858	0.0008	NWMN_RS09875	-1.1686	0.0032	NWMN_RS14280	-1.3105	0.0067	NWMN_RS03535	-1.3001	0.0000
59				NWMN_RS04960	-1.0850	0.0008	NWMN_RS13250	-1.1654	0.0027	NWMN_RS12790	-1.3091	0.0000	NWMN_RS04750	-1.2884	0.0000
60				NWMN_RS00820	-1.0845	0.0282	NWMN_RS05150	-1.1649	0.0035	NWMN_RS09400	-1.3079	0.0466	NWMN_RS09940	-1.2846	0.0236
61				NWMN_RS11500	-1.0833	0.0024	NWMN_RS11485	-1.1540	0.0001	NWMN_RS03535	-1.3027	0.0000	NWMN_RS11185	-1.2815	0.0005
62				NWMN_RS11840	-1.0817	0.0006	NWMN_RS11990	-1.1412	0.0094	NWMN_RS02450	-1.3001	0.0002	NWMN_RS06170	-1.2721	0.0002
63				NWMN_RS09760	-1.0735	0.0068	NWMN_RS12800	-1.1319	0.0001	NWMN_RS14390	-1.2927	0.0008	NWMN_RS13770	-1.2642	0.0347
64				NWMN_RS05545	-1.0708	0.0077	NWMN_RS03715	-1.1133	0.0000	NWMN_RS02775	-1.2831	0.0140	NWMN_RS05445	-1.2640	0.0004
65				NWMN_RS00200	-1.0700	0.0071	NWMN_RS14295	-1.1048	0.0025	NWMN_RS11500	-1.2816	0.0002	NWMN_RS13435	-1.2611	0.0001
66				NWMN_RS03560	-1.0696	0.0004	NWMN_RS04960	-1.1010	0.0006	NWMN_RS04335	-1.2769	0.0000	NWMN_RS13130	-1.2585	0.0002
67				NWMN_RS14880	-1.0688	0.0183	NWMN_RS02770	-1.0960	0.0336	NWMN_RS14910	-1.2672	0.0073	NWMN_RS07400	-1.2584	0.0016
68				NWMN_RS03715	-1.0679	0.0000	NWMN_RS02775	-1.0922	0.0402	NWMN_RS04780	-1.2494	0.0334	NWMN_RS07925	-1.2569	0.0222
69				NWMN_RS14285	-1.0641	0.0185	NWMN_RS02450	-1.0915	0.0019	NWMN_RS11670	-1.2464	0.0000	NWMN_RS06950	-1.2564	0.0003
70				NWMN_RS09205	-1.0613	0.0030	NWMN_RS06155	-1.0905	0.0020	NWMN_RS12480	-1.2385	0.0013	NWMN_RS00120	-1.2452	0.0000
71				NWMN_RS02450	-1.0568	0.0028	NWMN_RS12975	-1.0891	0.0060	NWMN_RS06030	-1.2357	0.0003	NWMN_RS09745	-1.2372	0.0001
72				NWMN_RS07065	-1.0530	0.0037	NWMN_RS04580	-1.0860	0.0001	NWMN_RS11725	-1.2345	0.0001	NWMN_RS09825	-1.2346	0.0018
73				NWMN_RS09380	-1.0512	0.0442	NWMN_RS00120	-1.0846	0.0002	NWMN_RS13850	-1.2299	0.0010	NWMN_RS14285	-1.2329	0.0050
74				NWMN_RS12800	-1.0496	0.0003	NWMN_RS03800	-1.0845	0.0000	NWMN_RS01110	-1.2253	0.0073	NWMN_RS13145	-1.2325	0.0002
75				NWMN_RS14735	-1.0467	0.0220	NWMN_RS06790	-1.0785	0.0327	NWMN_RS09950	-1.2249	0.0051	NWMN_RS03920	-1.2312	0.0000
76				NWMN_RS09360	-1.0439	0.0000	NWMN_RS01110	-1.0762	0.0192	NWMN_RS14295	-1.2243	0.0007	NWMN_RS13950	-1.2311	0.0148
77				NWMN_RS06735	-1.0378	0.0053	NWMN_RS13610	-1.0760	0.0190	NWMN_RS07220	-1.2229	0.0497	NWMN_RS13880	-1.2141	0.0004
78				NWMN_RS13950	-1.0280	0.0445	NWMN_RS07065	-1.0731	0.0025	NWMN_RS12740	-1.2089	0.0004	NWMN_RS07255	-1.2112	0.0006
79				NWMN_RS05375	-1.0254	0.0000	NWMN_RS14180	-1.0596	0.0016	NWMN_RS09815	-1.2083	0.0007	NWMN_RS09670	-1.2100	0.0030
80				NWMN_RS14715	-1.0247	0.0243	NWMN_RS09880	-1.0554	0.0096	NWMN_RS02380	-1.2069	0.0003	NWMN_RS13850	-1.2040	0.0017
81				NWMN_RS05370	-1.0235	0.0001	NWMN_RS04710	-1.0477	0.0005	NWMN_RS03660	-1.2042	0.0058	NWMN_RS10600	-1.1997	0.0001
82				NWMN_RS12830	-1.0090	0.0092	NWMN_RS05545	-1.0460	0.0095	NWMN_RS09700	-1.2017	0.0001	NWMN_RS12970	-1.1993	0.0072
83				NWMN_RS08105	-1.0068	0.0079	NWMN_RS13435	-1.0439	0.0013	NWMN_RS09675	-1.1988	0.0071	NWMN_RS09965	-1.1986	0.0093
84				NWMN_RS08745	-1.0063	0.0003	NWMN_RS09980	-1.0436	0.0241	NWMN_RS03755	-1.1971	0.0078	NWMN_RS13655	-1.1978	0.0064
85				NWMN_RS11540	-0.9935	0.0055	NWMN_RS11205	-1.0420	0.0022	NWMN_RS07030	-1.1967	0.0001	NWMN_RS07085	-1.1913	0.0006
86				NWMN_RS05530	-0.9928	0.0005	NWMN_RS03010	-1.0360	0.0471	NWMN_RS03330	-1.1960	0.0000	NWMN_RS04600	-1.1904	0.0001
87				NWMN_RS04715	-0.9880	0.0009	NWMN_RS12935	-1.0337	0.0222	NWMN_RS07255	-1.1942	0.0005	NWMN_RS03095	-1.1897	0.0134
88				NWMN_RS12775	-0.9775	0.0328	NWMN_RS05375	-1.0333	0.0000	NWMN_RS11540	-1.1857	0.0005	NWMN_RS00320	-1.1768	0.0015
89				NWMN_RS03605	-0.9714	0.0099	NWMN_RS03005	-1.0308	0.0020	NWMN_RS03255	-1.1849	0.0347	NWMN_RS06155	-1.1741	0.0017
90				NWMN_RS11145	-0.9712	0.0127	NWMN_RS06170	-1.0287	0.0028	NWMN_RS07430	-1.1801	0.0021	NWMN_RS07135	-1.1735	0.0002
91				NWMN_RS04965	-0.9644	0.0040	NWMN_RS06990	-1.0277	0.0203	NWMN_RS07965	-1.1744	0.0000	NWMN_RS00715	-1.1713	0.0000
92				NWMN_RS04705	-0.9627	0.0007	NWMN_RS04935	-1.0257	0.0001	NWMN_RS07085	-1.1741	0.0007	NWMN_RS08115	-1.1709	0.0002
93				NWMN_RS07430	-0.9605	0.0172	NWMN_RS05480	-1.0223	0.0002	NWMN_RS03245	-1.1707	0.0236	NWMN_RS09890	-1.1662	0.0085
94				NWMN_RS10680	-0.9593	0.0046	NWMN_RS14350	-1.0088	0.0359	NWMN_RS14290	-1.1706	0.0001	NWMN_RS11540	-1.1658	0.0006
95				NWMN_RS04315	-0.9538	0.0298	NWMN_RS14290	-1.0068	0.0009	NWMN_RS05035	-1.1700	0.0008	NWMN_RS07030	-1.1630	0.0002
96				NWMN_RS09815	-0.9519	0.0122	NWMN_RS02095	-1.0010	0.0211	NWMN_RS13435	-1.1698	0.0003	NWMN_RS03010	-1.1558	0.0314
97				NWMN_RS03835	-0.9497	0.0020	NWMN_RS06030	-0.9972	0.0045	NWMN_RS12795	-1.1695	0.0007	NWMN_RS12800	-1.1473	0.0002
98				NWMN_RS12845	-0.9493	0.0389	NWMN_RS12790	-0.9911	0.0009	NWMN_RS13845	-1.1558	0.0111	NWMN_RS14185	-1.1465	0.0317
99				NWMN_RS04755	-0.9475	0.0226	NWMN_RS06260	-0.9910	0.0032	NWMN_RS05030	-1.1541	0.0008	NWMN_RS03615	-1.1384	0.0033
100				NWMN_RS13575	-0.9460	0.0091	NWMN_RS04050	-0.9896	0.0019	NWMN_RS13460	-1.1449	0.0000	NWMN_RS13445	-1.1369	0.0018
101				NWMN_RS14910	-0.9417	0.0219	NWMN_RS13945	-0.9874	0.0039	NWMN_RS04960	-1.1404	0.0004	NWMN_RS04935	-1.1369	0.0000
102				NWMN_RS05365	-0.9374	0.0162	NWMN_RS04600	-0.9850	0.0013	NWMN_RS05360	-1.1398	0.0000	NWMN_RS04375	-1.1347	0.0000
103				NWMN_RS05445	-0.9361	0.0115	NWMN_RS09895	-0.9840	0.0149	NWMN_RS09880	-1.1352	0.0052	NWMN_RS02970	-1.1314	0.0003
104				NWMN_RS06035	-0.9350	0.0119	NWMN_RS12475	-0.9837	0.0007	NWMN_RS10715	-1.1346	0.0086	NWMN_RS14280	-1.1284	0.0229
105				NWMN_RS01430	-0.9337	0.0054	NWMN_RS03745	-0.9762	0.0044	NWMN_RS06170	-1.1318	0.0009	NWMN_RS09735	-1.1281	0.0007
106				NWMN_RS04175	-0.9318	0.0031	NWMN_RS01955	-0.9743	0.0050	NWMN_RS13770	-1.1289	0.0404	NWMN_RS09815	-1.1275	0.0020
107				NWMN_RS12955	-0.9318	0.0020	NWMN_RS11500	-0.9654	0.0065	NWMN_RS09895	-1.1262	0.0056	NWMN_RS03330	-1.1230	0.0001
108				NWMN_RS07860	-0.9260	0.0041	NWMN_RS07085	-0.9619	0.0071	NWMN_RS06135	-1.1116	0.0288	NWMN_RS12460	-1.1147	0.0002
109				NWMN_RS01865	-0.9206	0.0091	NWMN_RS09280	-0.9618	0.0001	NWMN_RS04320	-1.1113	0.0024	NWMN_RS04650	-1.1112	0.0090
110				NWMN_RS04600	-0.9168	0.0039	NWMN_RS11725	-0.9618	0.0031	NWMN_RS13605	-1.1096	0.0176	NWMN_RS09675	-1.1104	0.0160
111				NWMN_RS10780	-0.9114	0.0323	NWMN_RS05445	-0.9611	0.0075	NWMN_RS07210	-1.1070	0.0092	NWMN_RS03560	-1.1040	0.0009

TABLE S4A continued

	Time 2 min			Time 5 min			Time 10 min			Time 20 min			Time 30 min		
#	gene ID	log2 Fold Change	p adj. value	gene ID	log2 Fold Change	p adj. value	gene ID	log2 Fold Change	p adj. value	gene ID	log2 Fold Change	p adj. value	gene ID	log2 Fold Change	p adj. value
112				NWMN_RS12795	-0.9089	0.0069	NWMN_RS04875	-0.9587	0.0001	NWMN_RS04935	-1.1069	0.0000	NWMN_RS09205	-1.1027	0.0020
113				NWMN_RS09435	-0.9054	0.0323	NWMN_RS02265	-0.9583	0.0101	NWMN_RS05080	-1.0926	0.0452	NWMN_RS13250	-1.1003	0.0132
114				NWMN_RS09210	-0.8981	0.0134	NWMN_RS09800	-0.9566	0.0090	NWMN_RS06260	-1.0862	0.0013	NWMN_RS12805	-1.0992	0.0148
115				NWMN_RS08885	-0.8928	0.0137	NWMN_RS02945	-0.9562	0.0000	NWMN_RS03450	-1.0844	0.0010	NWMN_RS01115	-1.0969	0.0032
116				NWMN_RS05155	-0.8924	0.0432	NWMN_RS04965	-0.9561	0.0035	NWMN_RS03005	-1.0836	0.0013	NWMN_RS01890	-1.0940	0.0002
117				NWMN_RS03545	-0.8913	0.0040	NWMN_RS05365	-0.9541	0.0178	NWMN_RS15765	-1.0766	0.0124	NWMN_RS11795	-1.0936	0.0010
118				NWMN_RS09355	-0.8857	0.0168	NWMN_RS02760	-0.9503	0.0261	NWMN_RS03010	-1.0744	0.0405	NWMN_RS11815	-1.0930	0.0001
119				NWMN_RS03800	-0.8843	0.0001	NWMN_RS04350	-0.9301	0.0028	NWMN_RS02945	-1.0650	0.0000	NWMN_RS11820	-1.0930	0.0001
120				NWMN_RS14390	-0.8811	0.0302	NWMN_RS09675	-0.9201	0.0410	NWMN_RS04600	-1.0631	0.0005	NWMN_RS14290	-1.0922	0.0005
121				NWMN_RS04580	-0.8805	0.0015	NWMN_RS08115	-0.9193	0.0040	NWMN_RS10645	-1.0627	0.0029	NWMN_RS12480	-1.0902	0.0062
122				NWMN_RS14290	-0.8753	0.0052	NWMN_RS05340	-0.9171	0.0033	NWMN_RS09865	-1.0596	0.0009	NWMN_RS04320	-1.0875	0.0046
123				NWMN_RS06155	-0.8752	0.0163	NWMN_RS09060	-0.9129	0.0209	NWMN_RS02970	-1.0556	0.0005	NWMN_RS15125	-1.0872	0.0353
124				NWMN_RS08115	-0.8704	0.0088	NWMN_RS09275	-0.9103	0.0002	NWMN_RS13145	-1.0550	0.0019	NWMN_RS02450	-1.0807	0.0035
125				NWMN_RS06650	-0.8704	0.0037	NWMN_RS14285	-0.9102	0.0421	NWMN_RS04750	-1.0546	0.0000	NWMN_RS05370	-1.0801	0.0000
126				NWMN_RS13160	-0.8702	0.0014	NWMN_RS03920	-0.8875	0.0005	NWMN_RS14285	-1.0523	0.0168	NWMN_RS09950	-1.0749	0.0170
127				NWMN_RS04375	-0.8694	0.0013	NWMN_RS04750	-0.8872	0.0002	NWMN_RS09375	-1.0481	0.0041	NWMN_RS11940	-1.0739	0.0420
128				NWMN_RS03365	-0.8663	0.0489	NWMN_RS03440	-0.8864	0.0006	NWMN_RS06990	-1.0456	0.0180	NWMN_RS07170	-1.0688	0.0003
129				NWMN_RS12980	-0.8634	0.0042	NWMN_RS00435	-0.8835	0.0002	NWMN_RS13150	-1.0441	0.0308	NWMN_RS03150	-1.0677	0.0003
130				NWMN_RS00120	-0.8610	0.0040	NWMN_RS07295	-0.8812	0.0109	NWMN_RS00715	-1.0425	0.0001	NWMN_RS13750	-1.0645	0.0023
131				NWMN_RS03920	-0.8543	0.0007	NWMN_RS03480	-0.8801	0.0000	NWMN_RS07295	-1.0404	0.0023	NWMN_RS09935	-1.0622	0.0415
132				NWMN_RS05025	-0.8502	0.0007	NWMN_RS15765	-0.8789	0.0452	NWMN_RS13250	-1.0377	0.0120	NWMN_RS11260	-1.0606	0.0402
133				NWMN_RS13485	-0.8492	0.0000	NWMN_RS11670	-0.8787	0.0004	NWMN_RS01865	-1.0336	0.0087	NWMN_RS02090	-1.0589	0.0012
134				NWMN_RS03440	-0.8457	0.0013	NWMN_RS06250	-0.8755	0.0050	NWMN_RS12460	-1.0323	0.0005	NWMN_RS07110	-1.0531	0.0005
135				NWMN_RS05435	-0.8433	0.0022	NWMN_RS05345	-0.8733	0.0000	NWMN_RS08115	-1.0319	0.0011	NWMN_RS12475	-1.0460	0.0009
136				NWMN_RS04350	-0.8426	0.0092	NWMN_RS12480	-0.8691	0.0290	NWMN_RS10610	-1.0302	0.0136	NWMN_RS09740	-1.0443	0.0030
137				NWMN_RS06250	-0.8332	0.0086	NWMN_RS07725	-0.8685	0.0031	NWMN_RS02140	-1.0291	0.0014	NWMN_RS03715	-1.0414	0.0000
138				NWMN_RS05600	-0.8331	0.0387	NWMN_RS05370	-0.8683	0.0008	NWMN_RS11380	-1.0264	0.0020	NWMN_RS02380	-1.0394	0.0027
139				NWMN_RS14175	-0.8327	0.0021	NWMN_RS12980	-0.8646	0.0041	NWMN_RS11185	-1.0247	0.0061	NWMN_RS12710	-1.0350	0.0127
140				NWMN_RS10790	-0.8287	0.0387	NWMN_RS06740	-0.8643	0.0000	NWMN_RS11820	-1.0245	0.0002	NWMN_RS14365	-1.0329	0.0013
141				NWMN_RS03745	-0.8262	0.0169	NWMN_RS07110	-0.8642	0.0046	NWMN_RS05890	-1.0206	0.0409	NWMN_RS04640	-1.0303	0.0004
142				NWMN_RS02380	-0.8257	0.0162	NWMN_RS09385	-0.8640	0.0002	NWMN_RS05365	-1.0198	0.0170	NWMN_RS02140	-1.0283	0.0017
143				NWMN_RS11185	-0.8256	0.0360	NWMN_RS04715	-0.8626	0.0040	NWMN_RS13655	-1.0191	0.0160	NWMN_RS04645	-1.0277	0.0002
144				NWMN_RS07170	-0.8247	0.0077	NWMN_RS04175	-0.8537	0.0060	NWMN_RS09915	-1.0189	0.0392	NWMN_RS07190	-1.0269	0.0070
145				NWMN_RS04585	-0.8234	0.0157	NWMN_RS05145	-0.8482	0.0013	NWMN_RS09155	-1.0179	0.0000	NWMN_RS03325	-1.0237	0.0026
146				NWMN_RS08580	-0.8211	0.0293	NWMN_RS04375	-0.8480	0.0017	NWMN_RS12980	-1.0120	0.0009	NWMN_RS11245	-1.0235	0.0012
147				NWMN_RS11995	-0.8199	0.0004	NWMN_RS03560	-0.8440	0.0072	NWMN_RS00320	-1.0108	0.0072	NWMN_RS11045	-1.0231	0.0082
148				NWMN_RS04045	-0.8195	0.0079	NWMN_RS07255	-0.8432	0.0150	NWMN_RS04710	-1.0101	0.0009	NWMN_RS06260	-1.0227	0.0034
149				NWMN_RS06160	-0.8173	0.0092	NWMN_RS13010	-0.8418	0.0498	NWMN_RS04965	-1.0082	0.0021	NWMN_RS07965	-1.0219	0.0003
150				NWMN_RS08385	-0.8122	0.0093	NWMN_RS13075	-0.8407	0.0001	NWMN_RS07625	-1.0013	0.0021	NWMN_RS03725	-1.0196	0.0003
151				NWMN_RS02430	-0.8093	0.0345	NWMN_RS14965	-0.8400	0.0073	NWMN_RS13870	-1.0012	0.0094	NWMN_RS13150	-1.0131	0.0389
152				NWMN_RS10530	-0.8074	0.0033	NWMN_RS10600	-0.8389	0.0063	NWMN_RS03350	-0.9921	0.0092	NWMN_RS02690	-1.0122	0.0000
153				NWMN_RS03705	-0.8052	0.0053	NWMN_RS12885	-0.8359	0.0022	NWMN_RS13985	-0.9891	0.0004	NWMN_RS09155	-1.0086	0.0001
154				NWMN_RS13250	-0.8040	0.0351	NWMN_RS05530	-0.8329	0.0061	NWMN_RS03600	-0.9772	0.0054	NWMN_RS04960	-1.0032	0.0024
155				NWMN_RS05950	-0.8001	0.0116	NWMN_RS04800	-0.8302	0.0013	NWMN_RS06155	-0.9772	0.0073	NWMN_RS10715	-1.0022	0.0252
156				NWMN_RS14615	-0.7989	0.0118	NWMN_RS11285	-0.8297	0.0005	NWMN_RS04745	-0.9766	0.0022	NWMN_RS11450	-1.0001	0.0105
157				NWMN_RS05360	-0.7964	0.0034	NWMN_RS05025	-0.8296	0.0010	NWMN_RS13880	-0.9754	0.0047	NWMN_RS09700	-0.9986	0.0018
158				NWMN_RS03460	-0.7962	0.0033	NWMN_RS07400	-0.8264	0.0399	NWMN_RS03715	-0.9701	0.0002	NWMN_RS04050	-0.9948	0.0023
159				NWMN_RS09105	-0.7953	0.0375	NWMN_RS04785	-0.8236	0.0381	NWMN_RS10600	-0.9694	0.0014	NWMN_RS04055	-0.9930	0.0020
160				NWMN_RS14295	-0.7895	0.0444	NWMN_RS02270	-0.8168	0.0128	NWMN_RS09745	-0.9668	0.0019	NWMN_RS05600	-0.9914	0.0134
161				NWMN_RS03655	-0.7881	0.0163	NWMN_RS12740	-0.8102	0.0158	NWMN_RS07170	-0.9542	0.0012	NWMN_RS14025	-0.9841	0.0121
162				NWMN_RS02190	-0.7848	0.0102	NWMN_RS01170	-0.8092	0.0338	NWMN_RS12935	-0.9534	0.0361	NWMN_RS04940	-0.9835	0.0012
163				NWMN_RS13945	-0.7805	0.0312	NWMN_RS06650	-0.8040	0.0067	NWMN_RS05480	-0.9529	0.0006	NWMN_RS09925	-0.9832	0.0421
164				NWMN_RS08100	-0.7792	0.0070	NWMN_RS08550	-0.8019	0.0017	NWMN_RS02690	-0.9500	0.0001	NWMN_RS07390	-0.9784	0.0099
165				NWMN_RS12895	-0.7782	0.0444	NWMN_RS13460	-0.8014	0.0014	NWMN_RS03560	-0.9484	0.0031	NWMN_RS03155	-0.9751	0.0000
166				NWMN_RS13115	-0.7733	0.0005	NWMN_RS06605	-0.7996	0.0042	NWMN_RS10800	-0.9478	0.0139	NWMN_RS06250	-0.9736	0.0026
167				NWMN_RS13145	-0.7714	0.0362	NWMN_RS04515	-0.7953	0.0103	NWMN_RS14715	-0.9444	0.0344	NWMN_RS06150	-0.9644	0.0099
168				NWMN_RS03725	-0.7690	0.0060	NWMN_RS03510	-0.7947	0.0007	NWMN_RS06695	-0.9432	0.0000	NWMN_RS03800	-0.9620	0.0000
169				NWMN_RS09700	-0.7676	0.0137	NWMN_RS09105	-0.7944	0.0385	NWMN_RS03125	-0.9414	0.0000	NWMN_RS10645	-0.9604	0.0092
170				NWMN_RS11985	-0.7640	0.0169	NWMN_RS07170	-0.7863	0.0093	NWMN_RS05525	-0.9392	0.0032	NWMN_RS03440	-0.9596	0.0003

TABLE S4A continued

	Time 2 min			Time 5 min			Time 10 min			Time 20 min			Time 30 min		
#	gene ID	log2 Fold Change	p adj. value	gene ID	log2 Fold Change	p adj. value	gene ID	log2 Fold Change	p adj. value	gene ID	log2 Fold Change	p adj. value	gene ID	log2 Fold Change	p adj. value
171				NWMN_RS05175	-0.7630	0.0000	NWMN_RS06735	-0.7859	0.0390	NWMN_RS12605	-0.9383	0.0036	NWMN_RS04965	-0.9590	0.0040
172				NWMN_RS03360	-0.7618	0.0301	NWMN_RS05360	-0.7841	0.0037	NWMN_RS07110	-0.9380	0.0019	NWMN_RS03120	-0.9584	0.0001
173				NWMN_RS06170	-0.7597	0.0419	NWMN_RS10645	-0.7839	0.0311	NWMN_RS09740	-0.9369	0.0079	NWMN_RS11225	-0.9583	0.0005
174				NWMN_RS12665	-0.7583	0.0434	NWMN_RS13145	-0.7822	0.0278	NWMN_RS03120	-0.9360	0.0001	NWMN_RS11725	-0.9549	0.0045
175				NWMN_RS09385	-0.7524	0.0015	NWMN_RS03505	-0.7800	0.0006	NWMN_RS04645	-0.9265	0.0010	NWMN_RS12960	-0.9544	0.0017
176				NWMN_RS12475	-0.7506	0.0115	NWMN_RS03155	-0.7757	0.0011	NWMN_RS03765	-0.9259	0.0088	NWMN_RS12935	-0.9509	0.0384
177				NWMN_RS07095	-0.7501	0.0095	NWMN_RS04705	-0.7756	0.0075	NWMN_RS04050	-0.9237	0.0043	NWMN_RS04175	-0.9498	0.0021
178				NWMN_RS05345	-0.7497	0.0005	NWMN_RS04640	-0.7685	0.0075	NWMN_RS03800	-0.9216	0.0000	NWMN_RS05545	-0.9442	0.0299
179				NWMN_RS11580	-0.7488	0.0182	NWMN_RS13500	-0.7683	0.0050	NWMN_RS06740	-0.9200	0.0000	NWMN_RS03050	-0.9433	0.0101
180				NWMN_RS00675	-0.7479	0.0007	NWMN_RS06265	-0.7659	0.0043	NWMN_RS03095	-0.9186	0.0399	NWMN_RS14615	-0.9419	0.0031
181				NWMN_RS07160	-0.7468	0.0107	NWMN_RS09740	-0.7638	0.0347	NWMN_RS07335	-0.9170	0.0010	NWMN_RS13890	-0.9414	0.0005
182				NWMN_RS03155	-0.7435	0.0022	NWMN_RS09205	-0.7637	0.0376	NWMN_RS09690	-0.9145	0.0330	NWMN_RS07760	-0.9409	0.0343
183				NWMN_RS07295	-0.7406	0.0425	NWMN_RS03605	-0.7627	0.0460	NWMN_RS03050	-0.9139	0.0108	NWMN_RS09435	-0.9392	0.0464
184				NWMN_RS03615	-0.7404	0.0494	NWMN_RS02380	-0.7608	0.0260	NWMN_RS05445	-0.9136	0.0120	NWMN_RS05365	-0.9388	0.0388
185				NWMN_RS04935	-0.7370	0.0092	NWMN_RS02690	-0.7576	0.0028	NWMN_RS02445	-0.9127	0.0085	NWMN_RS03450	-0.9383	0.0061
186				NWMN_RS05510	-0.7343	0.0058	NWMN_RS04645	-0.7562	0.0092	NWMN_RS05530	-0.9122	0.0050	NWMN_RS10680	-0.9370	0.0061
187				NWMN_RS12470	-0.7302	0.0402	NWMN_RS07095	-0.7556	0.0081	NWMN_RS09800	-0.9106	0.0135	NWMN_RS03005	-0.9361	0.0074
188				NWMN_RS10735	-0.7300	0.0260	NWMN_RS05440	-0.7540	0.0154	NWMN_RS05930	-0.9048	0.0031	NWMN_RS04635	-0.9358	0.0004
189				NWMN_RS12675	-0.7284	0.0310	NWMN_RS10485	-0.7534	0.0002	NWMN_RS11045	-0.9036	0.0167	NWMN_RS12785	-0.9280	0.0005
190				NWMN_RS06950	-0.7284	0.0494	NWMN_RS13105	-0.7514	0.0300	NWMN_RS07760	-0.9028	0.0407	NWMN_RS05480	-0.9264	0.0010
191				NWMN_RS07725	-0.7274	0.0157	NWMN_RS12605	-0.7498	0.0167	NWMN_RS05370	-0.9013	0.0005	NWMN_RS10800	-0.9256	0.0173
192				NWMN_RS02970	-0.7273	0.0201	NWMN_RS01865	-0.7489	0.0420	NWMN_RS13075	-0.8980	0.0000	NWMN_RS12980	-0.9193	0.0038
193				NWMN_RS03555	-0.7225	0.0126	NWMN_RS13485	-0.7486	0.0002	NWMN_RS11990	-0.8971	0.0474	NWMN_RS15685	-0.9122	0.0247
194				NWMN_RS03005	-0.7209	0.0439	NWMN_RS07995	-0.7454	0.0042	NWMN_RS12735	-0.8962	0.0009	NWMN_RS07295	-0.9085	0.0098
195				NWMN_RS13550	-0.7203	0.0052	NWMN_RS04745	-0.7424	0.0215	NWMN_RS13750	-0.8955	0.0101	NWMN_RS08380	-0.9059	0.0045
196				NWMN_RS13480	-0.7197	0.0086	NWMN_RS12010	-0.7422	0.0260	NWMN_RS12960	-0.8929	0.0029	NWMN_RS03065	-0.9058	0.0002
197				NWMN_RS04220	-0.7167	0.0125	NWMN_RS08385	-0.7399	0.0180	NWMN_RS09205	-0.8914	0.0134	NWMN_RS13985	-0.9050	0.0021
198				NWMN_RS08915	-0.7115	0.0311	NWMN_RS15840	-0.7387	0.0290	NWMN_RS04715	-0.8874	0.0031	NWMN_RS06735	-0.9048	0.0196
199				NWMN_RS09280	-0.7115	0.0042	NWMN_RS07335	-0.7378	0.0097	NWMN_RS05150	-0.8871	0.0326	NWMN_RS13075	-0.9009	0.0000
200				NWMN_RS06010	-0.7104	0.0016	NWMN_RS12595	-0.7335	0.0154	NWMN_RS03505	-0.8862	0.0001	NWMN_RS04565	-0.8998	0.0062
201				NWMN_RS13460	-0.7082	0.0062	NWMN_RS03450	-0.7319	0.0339	NWMN_RS03155	-0.8855	0.0002	NWMN_RS04745	-0.8971	0.0065
202				NWMN_RS10725	-0.7055	0.0343	NWMN_RS04225	-0.7294	0.0123	NWMN_RS10760	-0.8825	0.0001	NWMN_RS07020	-0.8959	0.0164
203				NWMN_RS04055	-0.7055	0.0250	NWMN_RS07860	-0.7269	0.0264	NWMN_RS10665	-0.8818	0.0002	NWMN_RS07160	-0.8932	0.0035
204				NWMN_RS06500	-0.7011	0.0404	NWMN_RS09865	-0.7264	0.0287	NWMN_RS05345	-0.8808	0.0000	NWMN_RS12735	-0.8917	0.0011
205				NWMN_RS03450	-0.6990	0.0495	NWMN_RS04565	-0.7263	0.0306	NWMN_RS10485	-0.8749	0.0000	NWMN_RS13155	-0.8907	0.0081
206				NWMN_RS10535	-0.6975	0.0144	NWMN_RS06320	-0.7240	0.0008	NWMN_RS08200	-0.8715	0.0245	NWMN_RS09070	-0.8906	0.0030
207				NWMN_RS05520	-0.6971	0.0070	NWMN_RS12950	-0.7220	0.0353	NWMN_RS08380	-0.8692	0.0065	NWMN_RS04350	-0.8866	0.0057
208				NWMN_RS00215	-0.6944	0.0457	NWMN_RS13160	-0.7206	0.0097	NWMN_RS02265	-0.8633	0.0223	NWMN_RS12740	-0.8846	0.0134
209				NWMN_RS07130	-0.6933	0.0202	NWMN_RS13890	-0.7203	0.0053	NWMN_RS01790	-0.8628	0.0008	NWMN_RS13460	-0.8833	0.0006
210				NWMN_RS03950	-0.6922	0.0133	NWMN_RS14175	-0.7197	0.0112	NWMN_RS12895	-0.8582	0.0286	NWMN_RS03490	-0.8813	0.0003
211				NWMN_RS13500	-0.6905	0.0155	NWMN_RS03460	-0.7196	0.0075	NWMN_RS04640	-0.8565	0.0029	NWMN_RS09880	-0.8765	0.0383
212				NWMN_RS07720	-0.6902	0.0374	NWMN_RS05550	-0.7183	0.0027	NWMN_RS03725	-0.8493	0.0023	NWMN_RS13140	-0.8761	0.0219
213				NWMN_RS10540	-0.6822	0.0211	NWMN_RS09315	-0.7149	0.0094	NWMN_RS13445	-0.8483	0.0194	NWMN_RS04660	-0.8761	0.0075
214				NWMN_RS10080	-0.6814	0.0233	NWMN_RS01465	-0.7135	0.0328	NWMN_RS11750	-0.8480	0.0143	NWMN_RS00725	-0.8711	0.0463
215				NWMN_RS07185	-0.6806	0.0037	NWMN_RS04660	-0.7096	0.0325	NWMN_RS06605	-0.8453	0.0024	NWMN_RS12950	-0.8696	0.0296
216				NWMN_RS03945	-0.6796	0.0233	NWMN_RS12985	-0.7095	0.0248	NWMN_RS10495	-0.8427	0.0266	NWMN_RS07335	-0.8693	0.0021
217				NWMN_RS04790	-0.6766	0.0211	NWMN_RS07965	-0.7088	0.0111	NWMN_RS07775	-0.8424	0.0002	NWMN_RS09865	-0.8668	0.0091
218				NWMN_RS07135	-0.6750	0.0480	NWMN_RS03610	-0.7072	0.0265	NWMN_RS03325	-0.8382	0.0135	NWMN_RS01430	-0.8610	0.0180
219				NWMN_RS05535	-0.6743	0.0016	NWMN_RS07155	-0.7070	0.0150	NWMN_RS05545	-0.8379	0.0454	NWMN_RS05465	-0.8605	0.0054
220				NWMN_RS10070	-0.6708	0.0380	NWMN_RS13880	-0.7037	0.0498	NWMN_RS02385	-0.8376	0.0075	NWMN_RS11750	-0.8520	0.0152
221				NWMN_RS04225	-0.6707	0.0278	NWMN_RS07030	-0.7030	0.0315	NWMN_RS12985	-0.8334	0.0082	NWMN_RS03965	-0.8519	0.0024
222				NWMN_RS04890	-0.6688	0.0346	NWMN_RS12600	-0.7014	0.0343	NWMN_RS03150	-0.8298	0.0033	NWMN_RS11580	-0.8512	0.0147
223				NWMN_RS05440	-0.6668	0.0420	NWMN_RS07625	-0.6990	0.0395	NWMN_RS10640	-0.8254	0.0235	NWMN_RS12605	-0.8508	0.0126
224				NWMN_RS08230	-0.6645	0.0329	NWMN_RS08745	-0.6984	0.0180	NWMN_RS04350	-0.8241	0.0094	NWMN_RS02445	-0.8493	0.0186
225				NWMN_RS03455	-0.6635	0.0040	NWMN_RS08710	-0.6979	0.0252	NWMN_RS09275	-0.8235	0.0010	NWMN_RS02945	-0.8489	0.0003
226				NWMN_RS05945	-0.6624	0.0278	NWMN_RS09005	-0.6959	0.0121	NWMN_RS13890	-0.8228	0.0017	NWMN_RS05470	-0.8477	0.0078
227				NWMN_RS03710	-0.6607	0.0118	NWMN_RS09070	-0.6926	0.0176	NWMN_RS02145	-0.8202	0.0059	NWMN_RS04795	-0.8455	0.0055
228				NWMN_RS10730	-0.6582	0.0098	NWMN_RS05175	-0.6904	0.0003	NWMN_RS05520	-0.8149	0.0016	NWMN_RS04580	-0.8438	0.0061
229				NWMN_RS10600	-0.6536	0.0453	NWMN_RS11995	-0.6903	0.0038	NWMN_RS03700	-0.8145	0.0173	NWMN_RS13870	-0.8436	0.0357

TABLE S4A continued

	Time 2 min			Time 5 min			Time 10 min			Time 20 min			Time 30 min		
#	gene ID	log2 Fold Change	p adj. value	gene ID	log2 Fold Change	p adj. value	gene ID	log2 Fold Change	p adj. value	gene ID	log2 Fold Change	p adj. value	gene ID	log2 Fold Change	p adj. value
230				NWMN_RS12985	-0.6478	0.0447	NWMN_RS03545	-0.6883	0.0314	NWMN_RS09280	-0.8097	0.0014	NWMN_RS05510	-0.8422	0.0018
231				NWMN_RS06320	-0.6457	0.0035	NWMN_RS04045	-0.6870	0.0260	NWMN_RS04175	-0.8095	0.0095	NWMN_RS04515	-0.8416	0.0113
232				NWMN_RS12885	-0.6446	0.0278	NWMN_RS03125	-0.6846	0.0009	NWMN_RS04375	-0.8091	0.0033	NWMN_RS13585	-0.8409	0.0306
233				NWMN_RS05180	-0.6418	0.0002	NWMN_RS15775	-0.6832	0.0410	NWMN_RS04565	-0.8055	0.0147	NWMN_RS03700	-0.8402	0.0159
234				NWMN_RS05955	-0.6376	0.0167	NWMN_RS06700	-0.6803	0.0083	NWMN_RS03965	-0.8048	0.0042	NWMN_RS13875	-0.8399	0.0447
235				NWMN_RS07675	-0.6305	0.0163	NWMN_RS12735	-0.6783	0.0150	NWMN_RS08105	-0.8026	0.0419	NWMN_RS04710	-0.8392	0.0082
236				NWMN_RS04605	-0.6268	0.0416	NWMN_RS07135	-0.6771	0.0410	NWMN_RS05535	-0.8016	0.0003	NWMN_RS08845	-0.8382	0.0006
237				NWMN_RS07250	-0.6207	0.0157	NWMN_RS06785	-0.6758	0.0464	NWMN_RS14525	-0.7980	0.0052	NWMN_RS08580	-0.8374	0.0235
238				NWMN_RS00435	-0.6174	0.0132	NWMN_RS02385	-0.6755	0.0346	NWMN_RS01795	-0.7973	0.0110	NWMN_RS02430	-0.8374	0.0304
239				NWMN_RS07995	-0.6170	0.0173	NWMN_RS08230	-0.6747	0.0297	NWMN_RS12030	-0.7959	0.0417	NWMN_RS05490	-0.8348	0.0191
240				NWMN_RS07715	-0.6154	0.0302	NWMN_RS04055	-0.6721	0.0323	NWMN_RS05145	-0.7930	0.0036	NWMN_RS14060	-0.8338	0.0001
241				NWMN_RS10060	-0.6100	0.0065	NWMN_RS09360	-0.6700	0.0135	NWMN_RS09070	-0.7905	0.0070	NWMN_RS06265	-0.8318	0.0021
242				NWMN_RS07965	-0.6100	0.0352	NWMN_RS03065	-0.6698	0.0050	NWMN_RS06145	-0.7898	0.0043	NWMN_RS07720	-0.8307	0.0078
243				NWMN_RS14505	-0.6049	0.0129	NWMN_RS02970	-0.6669	0.0323	NWMN_RS05495	-0.7877	0.0034	NWMN_RS09280	-0.8301	0.0015
244				NWMN_RS04335	-0.5977	0.0274	NWMN_RS06420	-0.6632	0.0080	NWMN_RS08275	-0.7853	0.0013	NWMN_RS07095	-0.8290	0.0053
245				NWMN_RS02660	-0.5946	0.0010	NWMN_RS00065	-0.6606	0.0006	NWMN_RS02270	-0.7849	0.0169	NWMN_RS05525	-0.8284	0.0147
246				NWMN_RS07410	-0.5919	0.0023	NWMN_RS05180	-0.6595	0.0001	NWMN_RS08845	-0.7833	0.0013	NWMN_RS05355	-0.8282	0.0034
247				NWMN_RS11495	0.5858	0.0247	NWMN_RS07115	-0.6554	0.0025	NWMN_RS05355	-0.7807	0.0059	NWMN_RS03350	-0.8258	0.0369
248				NWMN_RS07530	0.5863	0.0365	NWMN_RS07695	-0.6529	0.0022	NWMN_RS04635	-0.7806	0.0025	NWMN_RS12455	-0.8235	0.0199
249				NWMN_RS08855	0.5887	0.0137	NWMN_RS03725	-0.6528	0.0209	NWMN_RS06735	-0.7790	0.0417	NWMN_RS04620	-0.8218	0.0085
250				NWMN_RS11085	0.5961	0.0302	NWMN_RS03835	-0.6519	0.0411	NWMN_RS07135	-0.7785	0.0166	NWMN_RS09105	-0.8215	0.0440
251				NWMN_RS07980	0.5992	0.0276	NWMN_RS06395	-0.6500	0.0303	NWMN_RS02950	-0.7773	0.0007	NWMN_RS07970	-0.8206	0.0001
252				NWMN_RS02590	0.6010	0.0380	NWMN_RS07730	-0.6497	0.0034	NWMN_RS09210	-0.7769	0.0356	NWMN_RS10640	-0.8186	0.0285
253				NWMN_RS07005	0.6023	0.0211	NWMN_RS06695	-0.6484	0.0035	NWMN_RS04940	-0.7758	0.0120	NWMN_RS04105	-0.8177	0.0314
254				NWMN_RS06730	0.6024	0.0463	NWMN_RS05950	-0.6480	0.0462	NWMN_RS12885	-0.7754	0.0051	NWMN_RS04735	-0.8162	0.0295
255				NWMN_RS08840	0.6062	0.0311	NWMN_RS04885	-0.6446	0.0077	NWMN_RS09005	-0.7748	0.0054	NWMN_RS04740	-0.8155	0.0104
256				NWMN_RS12500	0.6233	0.0475	NWMN_RS05520	-0.6432	0.0132	NWMN_RS05340	-0.7737	0.0156	NWMN_RS09375	-0.8132	0.0342
257				NWMN_RS09230	0.6323	0.0177	NWMN_RS05945	-0.6406	0.0343	NWMN_RS04040	-0.7720	0.0213	NWMN_RS10575	-0.8112	0.0101
258				NWMN_RS04530	0.6429	0.0155	NWMN_RS05525	-0.6396	0.0352	NWMN_RS04055	-0.7718	0.0142	NWMN_RS10760	-0.8079	0.0005
259				NWMN_RS09715	0.6540	0.0139	NWMN_RS10510	-0.6356	0.0018	NWMN_RS08580	-0.7691	0.0365	NWMN_RS06390	-0.8018	0.0055
260				NWMN_RS08285	0.6542	0.0293	NWMN_RS02765	-0.6355	0.0260	NWMN_RS12785	-0.7686	0.0044	NWMN_RS07860	-0.8014	0.0147
261				NWMN_RS12590	0.6553	0.0065	NWMN_RS07130	-0.6312	0.0346	NWMN_RS05950	-0.7670	0.0200	NWMN_RS00050	-0.8007	0.0347
262				NWMN_RS07535	0.6626	0.0029	NWMN_RS05355	-0.6312	0.0314	NWMN_RS11795	-0.7642	0.0281	NWMN_RS01790	-0.7934	0.0034
263				NWMN_RS00500	0.6639	0.0007	NWMN_RS12460	-0.6307	0.0464	NWMN_RS08205	-0.7617	0.0202	NWMN_RS11720	-0.7918	0.0031
264				NWMN_RS03160	0.6679	0.0006	NWMN_RS06390	-0.6253	0.0257	NWMN_RS03440	-0.7606	0.0041	NWMN_RS13945	-0.7904	0.0282
265				NWMN_RS08760	0.6701	0.0211	NWMN_RS07185	-0.6185	0.0080	NWMN_RS07155	-0.7599	0.0081	NWMN_RS13480	-0.7828	0.0063
266				NWMN_RS12570	0.6732	0.0163	NWMN_RS06600	-0.6180	0.0110	NWMN_RS00675	-0.7583	0.0017	NWMN_RS06160	-0.7804	0.0151
267				NWMN_RS08125	0.6745	0.0058	NWMN_RS06010	-0.6137	0.0081	NWMN_RS03460	-0.7579	0.0048	NWMN_RS05945	-0.7799	0.0151
268				NWMN_RS00765	0.6792	0.0169	NWMN_RS08220	-0.6137	0.0484	NWMN_RS06150	-0.7542	0.0406	NWMN_RS04040	-0.7773	0.0278
269				NWMN_RS02480	0.6857	0.0127	NWMN_RS10055	-0.6134	0.0049	NWMN_RS14365	-0.7529	0.0241	NWMN_RS03045	-0.7729	0.0006
270				NWMN_RS10655	0.6861	0.0420	NWMN_RS03120	-0.6072	0.0169	NWMN_RS05945	-0.7455	0.0153	NWMN_RS09210	-0.7724	0.0420
271				NWMN_RS12555	0.6878	0.0326	NWMN_RS03135	-0.6047	0.0050	NWMN_RS06265	-0.7448	0.0059	NWMN_RS04615	-0.7694	0.0059
272				NWMN_RS02935	0.6959	0.0371	NWMN_RS04635	-0.5893	0.0247	NWMN_RS07115	-0.7430	0.0005	NWMN_RS03730	-0.7688	0.0478
273				NWMN_RS00235	0.6962	0.0063	NWMN_RS01790	-0.5884	0.0217	NWMN_RS03670	-0.7421	0.0478	NWMN_RS05970	-0.7590	0.0249
274				NWMN_RS07470	0.6966	0.0111	NWMN_RS07520	0.5879	0.0169	NWMN_RS06690	-0.7342	0.0319	NWMN_RS06605	-0.7521	0.0089
275				NWMN_RS08415	0.6969	0.0425	NWMN_RS07530	0.5929	0.0296	NWMN_RS11485	-0.7339	0.0202	NWMN_RS10665	-0.7505	0.0021
276				NWMN_RS09125	0.7002	0.0334	NWMN_RS07535	0.5945	0.0080	NWMN_RS03855	-0.7337	0.0156	NWMN_RS06740	-0.7502	0.0006
277				NWMN_RS04415	0.7061	0.0471	NWMN_RS05190	0.5996	0.0186	NWMN_RS04580	-0.7310	0.0135	NWMN_RS11920	-0.7489	0.0441
278				NWMN_RS09820	0.7065	0.0159	NWMN_RS07905	0.6059	0.0329	NWMN_RS08740	-0.7305	0.0101	NWMN_RS04655	-0.7440	0.0010
279				NWMN_RS11620	0.7065	0.0474	NWMN_RS08185	0.6356	0.0026	NWMN_RS06950	-0.7280	0.0439	NWMN_RS05340	-0.7421	0.0228
280				NWMN_RS02940	0.7112	0.0193	NWMN_RS08990	0.6371	0.0411	NWMN_RS13155	-0.7274	0.0307	NWMN_RS04715	-0.7418	0.0170
281				NWMN_RS05565	0.7136	0.0034	NWMN_RS12555	0.6382	0.0490	NWMN_RS10730	-0.7229	0.0047	NWMN_RS09080	-0.7404	0.0274
282				NWMN_RS13790	0.7180	0.0181	NWMN_RS05580	0.6399	0.0022	NWMN_RS07725	-0.7206	0.0180	NWMN_RS08275	-0.7342	0.0030
283				NWMN_RS07000	0.7221	0.0391	NWMN_RS12575	0.6407	0.0289	NWMN_RS13945	-0.7158	0.0447	NWMN_RS10485	-0.7327	0.0005
284				NWMN_RS08650	0.7248	0.0221	NWMN_RS01220	0.6438	0.0080	NWMN_RS05105	-0.7156	0.0014	NWMN_RS05930	-0.7324	0.0226
285				NWMN_RS04525	0.7267	0.0042	NWMN_RS05395	0.6487	0.0472	NWMN_RS14180	-0.7138	0.0438	NWMN_RS04875	-0.7314	0.0057
286				NWMN_RS11105	0.7287	0.0398	NWMN_RS03770	0.6501	0.0396	NWMN_RS03510	-0.7134	0.0042	NWMN_RS07245	-0.7301	0.0245
287				NWMN_RS08290	0.7311	0.0183	NWMN_RS12590	0.6564	0.0059	NWMN_RS09000	-0.7132	0.0053	NWMN_RS07250	-0.7300	0.0030
288				NWMN_RS02340	0.7323	0.0432	NWMN_RS12560	0.6588	0.0257	NWMN_RS01890	-0.7104	0.0107	NWMN_RS12435	-0.7299	0.0453

TABLE S4A continued

	Time 2 min			Time 5 min			Time 10 min			Time 20 min			Time 30 min		
#	gene ID	log2 Fold Change	p adj. value	gene ID	log2 Fold Change	p adj. value	gene ID	log2 Fold Change	p adj. value	gene ID	log2 Fold Change	p adj. value	gene ID	log2 Fold Change	p adj. value
289				NWMN_RS11660	0.7338	0.0142	NWMN_RS05575	0.6645	0.0009	NWMN_RS14615	-0.7094	0.0265	NWMN_RS10080	-0.7296	0.0170
290				NWMN_RS12910	0.7347	0.0281	NWMN_RS04675	0.6654	0.0245	NWMN_RS03950	-0.7079	0.0116	NWMN_RS11770	-0.7295	0.0030
291				NWMN_RS02475	0.7431	0.0295	NWMN_RS14620	0.6678	0.0250	NWMN_RS09420	-0.7027	0.0141	NWMN_RS01275	-0.7272	0.0121
292				NWMN_RS03075	0.7434	0.0360	NWMN_RS00465	0.6775	0.0448	NWMN_RS13500	-0.6949	0.0125	NWMN_RS13160	-0.7262	0.0134
293				NWMN_RS14395	0.7445	0.0126	NWMN_RS11085	0.6790	0.0101	NWMN_RS04875	-0.6926	0.0086	NWMN_RS04705	-0.7254	0.0163
294				NWMN_RS05995	0.7524	0.0187	NWMN_RS06200	0.6820	0.0390	NWMN_RS05375	-0.6872	0.0048	NWMN_RS09000	-0.7245	0.0049
295				NWMN_RS12560	0.7764	0.0079	NWMN_RS05255	0.6823	0.0181	NWMN_RS06250	-0.6840	0.0348	NWMN_RS11670	-0.7222	0.0090
296				NWMN_RS12755	0.7799	0.0004	NWMN_RS00235	0.6847	0.0072	NWMN_RS10735	-0.6764	0.0454	NWMN_RS07775	-0.7210	0.0022
297				NWMN_RS09130	0.7809	0.0189	NWMN_RS05410	0.6864	0.0402	NWMN_RS04630	-0.6756	0.0040	NWMN_RS04625	-0.7210	0.0160
298				NWMN_RS12585	0.7890	0.0000	NWMN_RS12860	0.6879	0.0164	NWMN_RS06395	-0.6743	0.0257	NWMN_RS09065	-0.7180	0.0405
299				NWMN_RS00085	0.7894	0.0325	NWMN_RS08175	0.6972	0.0006	NWMN_RS03065	-0.6742	0.0057	NWMN_RS05955	-0.7176	0.0099
300				NWMN_RS01375	0.7903	0.0380	NWMN_RS13430	0.7000	0.0213	NWMN_RS14060	-0.6670	0.0022	NWMN_RS03655	-0.7169	0.0336
301				NWMN_RS00105	0.7925	0.0202	NWMN_RS12910	0.7067	0.0329	NWMN_RS11815	-0.6656	0.0177	NWMN_RS12600	-0.7139	0.0400
302				NWMN_RS03080	0.7994	0.0144	NWMN_RS00045	0.7074	0.0015	NWMN_RS03055	-0.6651	0.0005	NWMN_RS12955	-0.7107	0.0410
303				NWMN_RS02840	0.8003	0.0459	NWMN_RS08180	0.7114	0.0069	NWMN_RS04705	-0.6642	0.0259	NWMN_RS06445	-0.7097	0.0480
304				NWMN_RS07750	0.8163	0.0008	NWMN_RS13305	0.7123	0.0498	NWMN_RS06600	-0.6623	0.0065	NWMN_RS05025	-0.7034	0.0088
305				NWMN_RS13730	0.8215	0.0205	NWMN_RS10455	0.7131	0.0395	NWMN_RS11285	-0.6605	0.0075	NWMN_RS04605	-0.7032	0.0236
306				NWMN_RS03170	0.8220	0.0076	NWMN_RS04910	0.7131	0.0065	NWMN_RS06650	-0.6596	0.0308	NWMN_RS11380	-0.6998	0.0458
307				NWMN_RS06200	0.8311	0.0119	NWMN_RS05425	0.7162	0.0407	NWMN_RS05465	-0.6586	0.0256	NWMN_RS12985	-0.6997	0.0340
308				NWMN_RS04440	0.8338	0.0284	NWMN_RS03170	0.7182	0.0190	NWMN_RS07245	-0.6582	0.0429	NWMN_RS05345	-0.6980	0.0017
309				NWMN_RS01415	0.8341	0.0209	NWMN_RS11585	0.7364	0.0009	NWMN_RS03490	-0.6522	0.0099	NWMN_RS05145	-0.6920	0.0158
310				NWMN_RS09550	0.8493	0.0221	NWMN_RS07840	0.7415	0.0165	NWMN_RS10575	-0.6520	0.0406	NWMN_RS06650	-0.6911	0.0242
311				NWMN_RS04420	0.8502	0.0279	NWMN_RS05980	0.7487	0.0297	NWMN_RS07695	-0.6511	0.0024	NWMN_RS13500	-0.6810	0.0166
312				NWMN_RS04445	0.8518	0.0308	NWMN_RS09475	0.7515	0.0234	NWMN_RS03135	-0.6508	0.0041	NWMN_RS08205	-0.6782	0.0424
313				NWMN_RS03785	0.8520	0.0172	NWMN_RS05995	0.7539	0.0149	NWMN_RS00435	-0.6489	0.0095	NWMN_RS02385	-0.6779	0.0373
314				NWMN_RS08935	0.8596	0.0498	NWMN_RS14595	0.7549	0.0209	NWMN_RS13060	-0.6443	0.0190	NWMN_RS14055	-0.6754	0.0030
315				NWMN_RS13775	0.8641	0.0297	NWMN_RS04670	0.7552	0.0153	NWMN_RS08385	-0.6407	0.0453	NWMN_RS04630	-0.6716	0.0047
316				NWMN_RS01840	0.8728	0.0221	NWMN_RS07650	0.7592	0.0073	NWMN_RS07250	-0.6350	0.0102	NWMN_RS05105	-0.6710	0.0035
317				NWMN_RS08990	0.8745	0.0041	NWMN_RS04450	0.7595	0.0241	NWMN_RS03945	-0.6348	0.0373	NWMN_RS09275	-0.6683	0.0113
318				NWMN_RS09310	0.8758	0.0147	NWMN_RS01150	0.7706	0.0248	NWMN_RS14055	-0.6309	0.0052	NWMN_RS08325	-0.6663	0.0447
319				NWMN_RS03525	0.8864	0.0276	NWMN_RS04440	0.7740	0.0372	NWMN_RS04615	-0.6306	0.0237	NWMN_RS02145	-0.6654	0.0314
320				NWMN_RS08540	0.9093	0.0265	NWMN_RS00500	0.7827	0.0001	NWMN_RS04985	-0.6301	0.0195	NWMN_RS04300	-0.6650	0.0446
321				NWMN_RS02815	0.9131	0.0233	NWMN_RS14825	0.7830	0.0281	NWMN_RS09175	-0.6299	0.0000	NWMN_RS10710	-0.6636	0.0399
322				NWMN_RS04480	0.9218	0.0214	NWMN_RS07845	0.8008	0.0019	NWMN_RS02955	-0.6276	0.0036	NWMN_RS01470	-0.6635	0.0162
323				NWMN_RS12825	0.9222	0.0060	NWMN_RS06080	0.8117	0.0003	NWMN_RS05025	-0.6267	0.0173	NWMN_RS09420	-0.6635	0.0232
324				NWMN_RS04185	0.9302	0.0277	NWMN_RS07010	0.8124	0.0169	NWMN_RS07095	-0.6246	0.0348	NWMN_RS12810	-0.6624	0.0326
325				NWMN_RS06515	0.9316	0.0209	NWMN_RS03180	0.8153	0.0057	NWMN_RS11265	-0.6239	0.0180	NWMN_RS13060	-0.6590	0.0191
326				NWMN_RS13810	0.9395	0.0085	NWMN_RS07980	0.8215	0.0011	NWMN_RS05510	-0.6237	0.0213	NWMN_RS05375	-0.6584	0.0082
327				NWMN_RS04685	0.9508	0.0125	NWMN_RS07460	0.8239	0.0274	NWMN_RS08100	-0.6222	0.0388	NWMN_RS08740	-0.6557	0.0286
328				NWMN_RS04830	0.9567	0.0433	NWMN_RS12580	0.8280	0.0003	NWMN_RS02565	-0.6188	0.0316	NWMN_RS14125	-0.6547	0.0295
329				NWMN_RS03175	0.9644	0.0033	NWMN_RS02840	0.8379	0.0313	NWMN_RS05500	-0.6170	0.0107	NWMN_RS06395	-0.6502	0.0362
330				NWMN_RS01225	0.9677	0.0306	NWMN_RS10520	0.8388	0.0000	NWMN_RS06710	-0.6161	0.0020	NWMN_RS05495	-0.6499	0.0196
331				NWMN_RS06520	0.9693	0.0060	NWMN_RS05260	0.8423	0.0153	NWMN_RS02660	-0.6145	0.0030	NWMN_RS11210	-0.6441	0.0335
332				NWMN_RS04160	0.9703	0.0491	NWMN_RS12585	0.8429	0.0000	NWMN_RS00065	-0.6119	0.0019	NWMN_RS07015	-0.6382	0.0006
333				NWMN_RS03805	0.9723	0.0222	NWMN_RS09840	0.8483	0.0122	NWMN_RS06330	-0.6114	0.0061	NWMN_RS10550	-0.6361	0.0166
334				NWMN_RS00850	0.9774	0.0100	NWMN_RS13510	0.8487	0.0294	NWMN_RS14125	-0.6114	0.0370	NWMN_RS11265	-0.6312	0.0189
335				NWMN_RS05315	0.9776	0.0298	NWMN_RS11105	0.8515	0.0115	NWMN_RS04800	-0.6104	0.0244	NWMN_RS11215	-0.6295	0.0402
336				NWMN_RS04675	0.9858	0.0005	NWMN_RS01840	0.8531	0.0280	NWMN_RS05175	-0.6101	0.0019	NWMN_RS03280	-0.6265	0.0246
337				NWMN_RS00840	0.9939	0.0360	NWMN_RS04445	0.8531	0.0250	NWMN_RS04655	-0.6088	0.0080	NWMN_RS03060	-0.6260	0.0061
338				NWMN_RS01420	0.9980	0.0129	NWMN_RS14340	0.8606	0.0327	NWMN_RS07970	-0.6088	0.0035	NWMN_RS10085	-0.6252	0.0135
339				NWMN_RS11395	1.0000	0.0038	NWMN_RS08895	0.8619	0.0007	NWMN_RS06390	-0.6079	0.0334	NWMN_RS05000	-0.6245	0.0036
340				NWMN_RS03690	1.0040	0.0309	NWMN_RS04475	0.8691	0.0207	NWMN_RS09090	-0.6076	0.0148	NWMN_RS07130	-0.6242	0.0453
341				NWMN_RS07455	1.0130	0.0092	NWMN_RS14875	0.8729	0.0402	NWMN_RS08405	-0.5997	0.0001	NWMN_RS03650	-0.6238	0.0156
342				NWMN_RS00750	1.0165	0.0013	NWMN_RS03075	0.8807	0.0083	NWMN_RS07015	-0.5988	0.0012	NWMN_RS02660	-0.6169	0.0055
343				NWMN_RS04670	1.0255	0.0006	NWMN_RS07455	0.8824	0.0216	NWMN_RS05180	-0.5974	0.0008	NWMN_RS11220	-0.6153	0.0190
344				NWMN_RS01425	1.0318	0.0011	NWMN_RS07590	0.8917	0.0399	NWMN_RS11675	-0.5961	0.0321	NWMN_RS09385	-0.6130	0.0128
345				NWMN_RS13345	1.0422	0.0411	NWMN_RS05565	0.8988	0.0001	NWMN_RS08550	-0.5951	0.0309	NWMN_RS12885	-0.6098	0.0341
346				NWMN_RS12865	1.0491	0.0278	NWMN_RS12755	0.9005	0.0000	NWMN_RS04225	-0.5912	0.0474	NWMN_RS09790	-0.6077	0.0441
347				NWMN_RS03035	1.0503	0.0116	NWMN_RS11155	0.9013	0.0380	NWMN_RS13480	-0.5861	0.0386	NWMN_RS09090	-0.6044	0.0190

TABLE S4A continued

	Time 2 min			Time 5 min			Time 10 min			Time 20 min			Time 30 min		
#	gene ID	log2 Fold Change	p adj. value	gene ID	log2 Fold Change	p adj. value	gene ID	log2 Fold Change	p adj. value	gene ID	log2 Fold Change	p adj. value	gene ID	log2 Fold Change	p adj. value
348				NWMN_RS11405	1.0532	0.0035	NWMN_RS09755	0.9208	0.0090	NWMN_RS13975	0.5932	0.0242	NWMN_RS04380	-0.6030	0.0142
349				NWMN_RS10660	1.0872	0.0436	NWMN_RS06350	0.9339	0.0482	NWMN_RS06730	0.6086	0.0358	NWMN_RS04385	-0.5944	0.0236
350				NWMN_RS07050	1.0952	0.0175	NWMN_RS14625	0.9389	0.0020	NWMN_RS05190	0.6112	0.0156	NWMN_RS05175	-0.5922	0.0036
351				NWMN_RS14105	1.0961	0.0000	NWMN_RS11150	0.9523	0.0161	NWMN_RS00500	0.6217	0.0047	NWMN_RS09750	-0.5876	0.0279
352				NWMN_RS05990	1.0995	0.0096	NWMN_RS02460	0.9561	0.0446	NWMN_RS12585	0.6332	0.0023	NWMN_RS07115	-0.5854	0.0083
353				NWMN_RS13255	1.1103	0.0181	NWMN_RS11395	0.9583	0.0045	NWMN_RS02195	0.6372	0.0406	NWMN_RS08250	0.5957	0.0335
354				NWMN_RS09165	1.1293	0.0115	NWMN_RS07465	0.9616	0.0004	NWMN_RS11775	0.6391	0.0004	NWMN_RS06825	0.5973	0.0042
355				NWMN_RS09470	1.1302	0.0016	NWMN_RS11405	0.9681	0.0066	NWMN_RS08315	0.6431	0.0014	NWMN_RS00500	0.6045	0.0101
356				NWMN_RS00125	1.1347	0.0181	NWMN_RS08890	0.9709	0.0001	NWMN_RS08990	0.6474	0.0370	NWMN_RS08340	0.6151	0.0219
357				NWMN_RS11400	1.1356	0.0006	NWMN_RS07005	0.9731	0.0000	NWMN_RS08310	0.6480	0.0020	NWMN_RS06730	0.6205	0.0333
358				NWMN_RS01830	1.1612	0.0373	NWMN_RS03070	0.9779	0.0003	NWMN_RS11085	0.6634	0.0135	NWMN_RS11085	0.6345	0.0223
359				NWMN_RS07460	1.1727	0.0012	NWMN_RS04415	0.9799	0.0029	NWMN_RS05395	0.6642	0.0403	NWMN_RS08670	0.6441	0.0196
360				NWMN_RS12880	1.1736	0.0375	NWMN_RS05485	0.9818	0.0184	NWMN_RS04670	0.6645	0.0361	NWMN_RS08655	0.6446	0.0404
361				NWMN_RS09475	1.2021	0.0001	NWMN_RS03740	0.9843	0.0037	NWMN_RS11715	0.6864	0.0038	NWMN_RS07840	0.6457	0.0408
362				NWMN_RS07490	1.2073	0.0281	NWMN_RS12760	0.9867	0.0027	NWMN_RS00105	0.6868	0.0406	NWMN_RS01220	0.6581	0.0078
363				NWMN_RS12750	1.2162	0.0198	NWMN_RS09235	0.9880	0.0064	NWMN_RS11400	0.6925	0.0474	NWMN_RS02940	0.6611	0.0256
364				NWMN_RS00835	1.2270	0.0021	NWMN_RS01880	0.9925	0.0180	NWMN_RS11780	0.7057	0.0355	NWMN_RS07105	0.6665	0.0257
365				NWMN_RS07495	1.2362	0.0162	NWMN_RS00630	0.9934	0.0144	NWMN_RS06555	0.7084	0.0373	NWMN_RS04910	0.6786	0.0136
366				NWMN_RS00845	1.2371	0.0033	NWMN_RS04075	0.9939	0.0410	NWMN_RS06200	0.7096	0.0307	NWMN_RS11200	0.6850	0.0392
367				NWMN_RS00455	1.2374	0.0355	NWMN_RS11400	0.9942	0.0028	NWMN_RS12580	0.7107	0.0035	NWMN_RS01835	0.6886	0.0311
368				NWMN_RS13180	1.2448	0.0214	NWMN_RS00400	0.9945	0.0143	NWMN_RS08865	0.7136	0.0330	NWMN_RS09475	0.6912	0.0401
369				NWMN_RS00275	1.2542	0.0207	NWMN_RS00085	0.9980	0.0038	NWMN_RS07890	0.7150	0.0318	NWMN_RS04230	0.6946	0.0192
370				NWMN_RS14005	1.2551	0.0018	NWMN_RS04685	0.9983	0.0080	NWMN_RS13965	0.7159	0.0277	NWMN_RS03175	0.7045	0.0373
371				NWMN_RS03520	1.2640	0.0103	NWMN_RS04165	0.9992	0.0038	NWMN_RS12760	0.7169	0.0414	NWMN_RS06580	0.7091	0.0136
372				NWMN_RS00830	1.2767	0.0139	NWMN_RS03090	1.0065	0.0108	NWMN_RS11395	0.7228	0.0393	NWMN_RS08865	0.7190	0.0359
373				NWMN_RS03775	1.2783	0.0027	NWMN_RS04760	1.0111	0.0061	NWMN_RS00465	0.7298	0.0291	NWMN_RS07140	0.7236	0.0246
374				NWMN_RS00740	1.2835	0.0040	NWMN_RS02330	1.0130	0.0041	NWMN_RS04290	0.7339	0.0279	NWMN_RS06200	0.7272	0.0277
375				NWMN_RS01350	1.3005	0.0457	NWMN_RS00775	1.0130	0.0029	NWMN_RS02340	0.7376	0.0468	NWMN_RS14960	0.7365	0.0341
376				NWMN_RS03695	1.3172	0.0030	NWMN_RS04425	1.0157	0.0042	NWMN_RS13970	0.7398	0.0052	NWMN_RS08805	0.7386	0.0324
377				NWMN_RS00130	1.3317	0.0001	NWMN_RS12620	1.0158	0.0004	NWMN_RS00160	0.7511	0.0348	NWMN_RS03770	0.7411	0.0257
378				NWMN_RS00315	1.3533	0.0014	NWMN_RS11895	1.0192	0.0163	NWMN_RS11405	0.7522	0.0410	NWMN_RS11195	0.7423	0.0446
379				NWMN_RS14935	1.3624	0.0483	NWMN_RS03080	1.0276	0.0007	NWMN_RS04215	0.7536	0.0469	NWMN_RS04440	0.7430	0.0458
380				NWMN_RS03780	1.3761	0.0041	NWMN_RS11110	1.0329	0.0161	NWMN_RS12560	0.7566	0.0090	NWMN_RS11390	0.7541	0.0357
381				NWMN_RS02335	1.4011	0.0115	NWMN_RS03685	1.0368	0.0110	NWMN_RS14030	0.7592	0.0498	NWMN_RS06725	0.7579	0.0000
382				NWMN_RS00745	1.4109	0.0016	NWMN_RS04260	1.0538	0.0315	NWMN_RS14630	0.7617	0.0089	NWMN_RS14845	0.7674	0.0307
383				NWMN_RS13820	1.4131	0.0165	NWMN_RS13135	1.0589	0.0297	NWMN_RS02235	0.7741	0.0450	NWMN_RS11090	0.7703	0.0299
384				NWMN_RS13815	1.5289	0.0002	NWMN_RS14600	1.0595	0.0015	NWMN_RS07455	0.7746	0.0458	NWMN_RS06560	0.7740	0.0236
385				NWMN_RS11480	1.5394	0.0163	NWMN_RS14380	1.0620	0.0019	NWMN_RS08345	0.7843	0.0406	NWMN_RS04455	0.7741	0.0313
386				NWMN_RS05130	1.6517	0.0221	NWMN_RS01835	1.0727	0.0002	NWMN_RS08360	0.7866	0.0006	NWMN_RS02935	0.7802	0.0138
387				NWMN_RS06310	1.6570	0.0072	NWMN_RS11115	1.0732	0.0059	NWMN_RS06520	0.7886	0.0249	NWMN_RS09715	0.7835	0.0022
388				NWMN_RS13190	1.6584	0.0002	NWMN_RS04185	1.0785	0.0068	NWMN_RS03070	0.7913	0.0065	NWMN_RS04450	0.7838	0.0192
389				NWMN_RS13900	1.7215	0.0425	NWMN_RS00755	1.0795	0.0008	NWMN_RS07840	0.7970	0.0089	NWMN_RS11385	0.7906	0.0446
390				NWMN_RS13185	1.7898	0.0004	NWMN_RS14415	1.0810	0.0297	NWMN_RS12825	0.7999	0.0167	NWMN_RS06635	0.7910	0.0196
391				NWMN_RS07320	1.7956	0.0077	NWMN_RS10655	1.0833	0.0004	NWMN_RS05425	0.8013	0.0196	NWMN_RS06745	0.7930	0.0115
392				NWMN_RS13195	1.8339	0.0016	NWMN_RS01885	1.0853	0.0095	NWMN_RS02845	0.8092	0.0103	NWMN_RS07665	0.7956	0.0001
393				NWMN_RS06295	1.9060	0.0122	NWMN_RS11120	1.0889	0.0412	NWMN_RS00150	0.8129	0.0404	NWMN_RS05410	0.7978	0.0169
394				NWMN_RS14150	1.9106	0.0066	NWMN_RS00780	1.0986	0.0061	NWMN_RS12555	0.8143	0.0096	NWMN_RS06510	0.7995	0.0373
395				NWMN_RS13200	2.0104	0.0007	NWMN_RS04420	1.1065	0.0022	NWMN_RS07985	0.8143	0.0478	NWMN_RS14030	0.8001	0.0386
396				NWMN_RS06305	2.0368	0.0016	NWMN_RS07000	1.1099	0.0005	NWMN_RS02245	0.8191	0.0170	NWMN_RS02845	0.8020	0.0121
397				NWMN_RS14155	2.2884	0.0016	NWMN_RS13925	1.1142	0.0297	NWMN_RS13065	0.8239	0.0132	NWMN_RS08700	0.8048	0.0434
398				NWMN_RS14160	2.3089	0.0052	NWMN_RS12865	1.1148	0.0165	NWMN_RS13775	0.8241	0.0426	NWMN_RS05965	0.8056	0.0234
399				NWMN_RS00865	2.3530	0.0002	NWMN_RS13440	1.1175	0.0217	NWMN_RS10105	0.8379	0.0158	NWMN_RS06630	0.8115	0.0480
400				NWMN_RS06300	2.3550	0.0006	NWMN_RS07470	1.1215	0.0000	NWMN_RS08935	0.8506	0.0429	NWMN_RS02340	0.8177	0.0315
401				NWMN_RS13205	2.4388	0.0000	NWMN_RS03175	1.1226	0.0003	NWMN_RS14215	0.8555	0.0296	NWMN_RS05425	0.8203	0.0181
402				NWMN_RS13210	2.6809	0.0000	NWMN_RS03680	1.1270	0.0004	NWMN_RS05270	0.8611	0.0138	NWMN_RS02225	0.8240	0.0455
403				NWMN_RS13230	2.8966	0.0008	NWMN_RS14630	1.1468	0.0000	NWMN_RS09475	0.8672	0.0074	NWMN_RS09345	0.8254	0.0210
404				NWMN_RS13220	3.4534	0.0007	NWMN_RS02625	1.1573	0.0151	NWMN_RS03080	0.8681	0.0054	NWMN_RS08650	0.8347	0.0060
405				NWMN_RS13225	3.6584	0.0001	NWMN_RS03675	1.1577	0.0050	NWMN_RS05980	0.8797	0.0088	NWMN_RS07980	0.8451	0.0011
406							NWMN_RS05270	1.1587	0.0005	NWMN_RS10755	0.8836	0.0358	NWMN_RS10520	0.8525	0.0000

TABLE S4A continued

	Time 2 min			Time 5 min			Time 10 min			Time 20 min			Time 30 min		
#	gene ID	log2 Fold Change	p adj. value	gene ID	log2 Fold Change	p adj. value	gene ID	log2 Fold Change	p adj. value	gene ID	log2 Fold Change	p adj. value	gene ID	log2 Fold Change	p adj. value
407							NWMN_RS00315	1.1605	0.0061	NWMN_RS06120	0.8869	0.0082	NWMN_RS02330	0.8659	0.0200
408							NWMN_RS02630	1.1615	0.0159	NWMN_RS14955	0.8870	0.0235	NWMN_RS07670	0.8663	0.0035
409							NWMN_RS10855	1.1617	0.0142	NWMN_RS01420	0.8882	0.0326	NWMN_RS02235	0.8708	0.0247
410							NWMN_RS13065	1.1648	0.0002	NWMN_RS03175	0.8913	0.0060	NWMN_RS00315	0.8727	0.0465
411							NWMN_RS02335	1.1664	0.0410	NWMN_RS03035	0.8916	0.0404	NWMN_RS04445	0.8755	0.0209
412							NWMN_RS04410	1.1716	0.0025	NWMN_RS03525	0.8935	0.0266	NWMN_RS06555	0.8757	0.0087
413							NWMN_RS11885	1.1767	0.0235	NWMN_RS01915	0.8958	0.0334	NWMN_RS04240	0.8772	0.0214
414							NWMN_RS09335	1.1787	0.0005	NWMN_RS12050	0.9036	0.0452	NWMN_RS12755	0.8783	0.0001
415							NWMN_RS04480	1.2050	0.0012	NWMN_RS01835	0.9059	0.0024	NWMN_RS08830	0.8799	0.0285
416							NWMN_RS02325	1.2169	0.0011	NWMN_RS14940	0.9065	0.0464	NWMN_RS00680	0.8861	0.0005
417							NWMN_RS13820	1.2200	0.0366	NWMN_RS07980	0.9083	0.0003	NWMN_RS02325	0.8876	0.0347
418							NWMN_RS13815	1.2232	0.0038	NWMN_RS10520	0.9089	0.0000	NWMN_RS11475	0.8883	0.0195
419							NWMN_RS11475	1.2281	0.0001	NWMN_RS08975	0.9116	0.0308	NWMN_RS00085	0.8911	0.0122
420							NWMN_RS12045	1.2286	0.0018	NWMN_RS01220	0.9219	0.0001	NWMN_RS00150	0.8990	0.0239
421							NWMN_RS11890	1.2452	0.0101	NWMN_RS11845	0.9229	0.0152	NWMN_RS00130	0.8998	0.0237
422							NWMN_RS00745	1.2471	0.0058	NWMN_RS02330	0.9265	0.0102	NWMN_RS14635	0.9026	0.0285
423							NWMN_RS13810	1.2546	0.0001	NWMN_RS00165	0.9281	0.0325	NWMN_RS00160	0.9049	0.0102
424							NWMN_RS12825	1.2560	0.0001	NWMN_RS06540	0.9347	0.0173	NWMN_RS03695	0.9111	0.0481
425							NWMN_RS06115	1.2619	0.0080	NWMN_RS07650	0.9351	0.0007	NWMN_RS09130	0.9150	0.0072
426							NWMN_RS00740	1.2684	0.0037	NWMN_RS10425	0.9469	0.0276	NWMN_RS06120	0.9188	0.0073
427							NWMN_RS02135	1.2719	0.0005	NWMN_RS00125	0.9488	0.0453	NWMN_RS05980	0.9246	0.0062
428							NWMN_RS07050	1.2772	0.0035	NWMN_RS06080	0.9499	0.0000	NWMN_RS06545	0.9365	0.0030
429							NWMN_RS04405	1.2776	0.0001	NWMN_RS01415	0.9513	0.0067	NWMN_RS11105	0.9396	0.0057
430							NWMN_RS13835	1.2837	0.0095	NWMN_RS14990	0.9583	0.0137	NWMN_RS10410	0.9415	0.0110
431							NWMN_RS10865	1.2842	0.0455	NWMN_RS05040	0.9601	0.0471	NWMN_RS14005	0.9428	0.0221
432							NWMN_RS03520	1.2916	0.0071	NWMN_RS11460	0.9688	0.0156	NWMN_RS11100	0.9446	0.0456
433							NWMN_RS13320	1.3119	0.0007	NWMN_RS01885	0.9694	0.0266	NWMN_RS08810	0.9465	0.0121
434							NWMN_RS14680	1.3322	0.0256	NWMN_RS15480	0.9701	0.0173	NWMN_RS14825	0.9546	0.0063
435							NWMN_RS13350	1.3428	0.0464	NWMN_RS12210	0.9734	0.0179	NWMN_RS11715	0.9554	0.0000
436							NWMN_RS04205	1.3574	0.0455	NWMN_RS00470	0.9747	0.0104	NWMN_RS12560	0.9646	0.0006
437							NWMN_RS01420	1.3606	0.0003	NWMN_RS08355	0.9790	0.0018	NWMN_RS14340	0.9676	0.0151
438							NWMN_RS00180	1.3616	0.0022	NWMN_RS07050	0.9812	0.0334	NWMN_RS00815	0.9693	0.0349
439							NWMN_RS08975	1.3630	0.0006	NWMN_RS01180	0.9830	0.0432	NWMN_RS14320	0.9698	0.0167
440							NWMN_RS12745	1.3632	0.0001	NWMN_RS14950	0.9836	0.0335	NWMN_RS01445	0.9929	0.0339
441							NWMN_RS03785	1.3814	0.0000	NWMN_RS14660	0.9850	0.0213	NWMN_RS15745	0.9938	0.0369
442							NWMN_RS14005	1.3848	0.0003	NWMN_RS11105	0.9858	0.0029	NWMN_RS01225	0.9950	0.0339
443							NWMN_RS09310	1.3871	0.0000	NWMN_RS13545	0.9894	0.0326	NWMN_RS13975	0.9962	0.0000
444							NWMN_RS05315	1.3933	0.0007	NWMN_RS13415	0.9977	0.0183	NWMN_RS09340	1.0126	0.0415
445							NWMN_RS14635	1.3933	0.0002	NWMN_RS05315	0.9989	0.0205	NWMN_RS00740	1.0152	0.0298
446							NWMN_RS13355	1.3937	0.0322	NWMN_RS00755	0.9995	0.0025	NWMN_RS11845	1.0171	0.0070
447							NWMN_RS11125	1.4130	0.0161	NWMN_RS12755	1.0005	0.0000	NWMN_RS14985	1.0309	0.0176
448							NWMN_RS05300	1.4136	0.0082	NWMN_RS06350	1.0025	0.0317	NWMN_RS08360	1.0352	0.0000
449							NWMN_RS10105	1.4173	0.0000	NWMN_RS02835	1.0031	0.0005	NWMN_RS02210	1.0378	0.0196
450							NWMN_RS00530	1.4182	0.0257	NWMN_RS13810	1.0157	0.0029	NWMN_RS09115	1.0412	0.0078
451							NWMN_RS04200	1.4222	0.0379	NWMN_RS01225	1.0168	0.0240	NWMN_RS13415	1.0452	0.0139
452							NWMN_RS02285	1.4290	0.0492	NWMN_RS04165	1.0208	0.0030	NWMN_RS02835	1.0498	0.0003
453							NWMN_RS11250	1.4317	0.0417	NWMN_RS08705	1.0268	0.0070	NWMN_RS08345	1.0518	0.0045
454							NWMN_RS14015	1.4380	0.0063	NWMN_RS01980	1.0285	0.0324	NWMN_RS04235	1.0529	0.0179
455							NWMN_RS04695	1.4398	0.0421	NWMN_RS11180	1.0286	0.0117	NWMN_RS02350	1.0593	0.0129
456							NWMN_RS14105	1.4457	0.0000	NWMN_RS14825	1.0391	0.0023	NWMN_RS13540	1.0596	0.0080
457							NWMN_RS00130	1.4467	0.0000	NWMN_RS09310	1.0441	0.0024	NWMN_RS11120	1.0647	0.0475
458							NWMN_RS07610	1.4542	0.0385	NWMN_RS13430	1.0480	0.0002	NWMN_RS00750	1.0683	0.0007
459							NWMN_RS14685	1.4608	0.0019	NWMN_RS04180	1.0496	0.0444	NWMN_RS12555	1.0712	0.0005
460							NWMN_RS14235	1.4608	0.0004	NWMN_RS06800	1.0684	0.0157	NWMN_RS14990	1.0733	0.0053
461							NWMN_RS01415	1.4615	0.0000	NWMN_RS04120	1.0690	0.0017	NWMN_RS05085	1.0751	0.0026
462							NWMN_RS00750	1.4678	0.0000	NWMN_RS11115	1.0761	0.0075	NWMN_RS06080	1.0821	0.0000
463							NWMN_RS07485	1.4730	0.0006	NWMN_RS11705	1.0801	0.0258	NWMN_RS10755	1.0832	0.0092
464							NWMN_RS03355	1.4799	0.0097	NWMN_RS00130	1.0862	0.0031	NWMN_RS11180	1.0902	0.0078
465							NWMN_RS03775	1.4838	0.0003	NWMN_RS00780	1.0885	0.0073	NWMN_RS04185	1.0936	0.0064

TABLE S4A continued

	Time 2 min			Time 5 min			Time 10 min			Time 20 min			Time 30 min		
#	gene ID	log2 Fold Change	p adj. value	gene ID	log2 Fold Change	p adj. value	gene ID	log2 Fold Change	p adj. value	gene ID	log2 Fold Change	p adj. value	gene ID	log2 Fold Change	p adj. value
466							NWMN_RS13545	1.4932	0.0006	NWMN_RS00740	1.0949	0.0153	NWMN_RS12215	1.0975	0.0160
467							NWMN_RS11705	1.4939	0.0012	NWMN_RS02630	1.0966	0.0233	NWMN_RS11115	1.1002	0.0084
468							NWMN_RS14010	1.4999	0.0038	NWMN_RS02135	1.1006	0.0033	NWMN_RS00125	1.1009	0.0190
469							NWMN_RS05225	1.5011	0.0187	NWMN_RS11110	1.1083	0.0100	NWMN_RS11455	1.1045	0.0039
470							NWMN_RS12850	1.5102	0.0008	NWMN_RS12620	1.1088	0.0001	NWMN_RS14325	1.1057	0.0053
471							NWMN_RS01390	1.5204	0.0005	NWMN_RS14115	1.1089	0.0356	NWMN_RS05165	1.1097	0.0016
472							NWMN_RS14385	1.5326	0.0011	NWMN_RS00085	1.1110	0.0011	NWMN_RS15480	1.1136	0.0056
473							NWMN_RS14855	1.5398	0.0097	NWMN_RS13835	1.1146	0.0275	NWMN_RS05040	1.1212	0.0190
474							NWMN_RS06120	1.5432	0.0000	NWMN_RS10765	1.1171	0.0153	NWMN_RS07050	1.1213	0.0147
475							NWMN_RS05230	1.5707	0.0164	NWMN_RS10660	1.1237	0.0323	NWMN_RS10770	1.1249	0.0319
476							NWMN_RS04160	1.5715	0.0004	NWMN_RS12745	1.1330	0.0014	NWMN_RS14015	1.1286	0.0392
477							NWMN_RS14930	1.5794	0.0073	NWMN_RS13925	1.1353	0.0256	NWMN_RS04685	1.1348	0.0036
478							NWMN_RS03690	1.5909	0.0001	NWMN_RS02210	1.1374	0.0081	NWMN_RS12750	1.1360	0.0254
479							NWMN_RS12205	1.5981	0.0355	NWMN_RS07205	1.1378	0.0005	NWMN_RS01915	1.1387	0.0059
480							NWMN_RS08995	1.5981	0.0006	NWMN_RS01385	1.1390	0.0173	NWMN_RS14010	1.1392	0.0347
481							NWMN_RS00440	1.5998	0.0080	NWMN_RS11570	1.1434	0.0317	NWMN_RS12850	1.1397	0.0167
482							NWMN_RS00410	1.6012	0.0495	NWMN_RS14235	1.1460	0.0081	NWMN_RS07490	1.1407	0.0340
483							NWMN_RS03805	1.6071	0.0000	NWMN_RS01080	1.1558	0.0135	NWMN_RS14115	1.1431	0.0315
484							NWMN_RS10460	1.6125	0.0010	NWMN_RS14575	1.1603	0.0356	NWMN_RS11175	1.1485	0.0009
485							NWMN_RS00460	1.6207	0.0000	NWMN_RS11175	1.1620	0.0008	NWMN_RS10690	1.1566	0.0137
486							NWMN_RS12080	1.6338	0.0066	NWMN_RS04185	1.1632	0.0031	NWMN_RS10430	1.1578	0.0005
487							NWMN_RS09165	1.6378	0.0001	NWMN_RS05300	1.1665	0.0344	NWMN_RS00280	1.1643	0.0414
488							NWMN_RS07320	1.6534	0.0180	NWMN_RS08350	1.1667	0.0082	NWMN_RS13020	1.1657	0.0352
489							NWMN_RS12750	1.6565	0.0005	NWMN_RS02015	1.1690	0.0358	NWMN_RS12715	1.1673	0.0018
490							NWMN_RS03810	1.6571	0.0000	NWMN_RS14635	1.1704	0.0028	NWMN_RS08355	1.1718	0.0001
491							NWMN_RS13520	1.6727	0.0000	NWMN_RS11900	1.1762	0.0125	NWMN_RS05565	1.1849	0.0000
492							NWMN_RS03780	1.6875	0.0002	NWMN_RS01390	1.1806	0.0092	NWMN_RS14940	1.1853	0.0074
493							NWMN_RS13180	1.6927	0.0007	NWMN_RS07490	1.1842	0.0258	NWMN_RS13430	1.1860	0.0000
494							NWMN_RS01385	1.6970	0.0002	NWMN_RS13795	1.1914	0.0033	NWMN_RS06100	1.1877	0.0419
495							NWMN_RS14140	1.6971	0.0008	NWMN_RS07300	1.1949	0.0186	NWMN_RS02240	1.1892	0.0075
496							NWMN_RS14935	1.7191	0.0084	NWMN_RS14750	1.1996	0.0349	NWMN_RS08705	1.1946	0.0014
497							NWMN_RS00305	1.7224	0.0005	NWMN_RS01910	1.2006	0.0409	NWMN_RS13425	1.1963	0.0278
498							NWMN_RS03300	1.7254	0.0461	NWMN_RS11800	1.2049	0.0044	NWMN_RS04160	1.1969	0.0099
499							NWMN_RS05130	1.7310	0.0162	NWMN_RS05565	1.2168	0.0000	NWMN_RS14955	1.2071	0.0013
500							NWMN_RS12095	1.7363	0.0286	NWMN_RS04115	1.2205	0.0003	NWMN_RS14105	1.2087	0.0000
501							NWMN_RS12050	1.7386	0.0000	NWMN_RS13505	1.2295	0.0002	NWMN_RS00365	1.2137	0.0304
502							NWMN_RS04340	1.7407	0.0307	NWMN_RS07070	1.2298	0.0456	NWMN_RS06940	1.2225	0.0166
503							NWMN_RS13660	1.7476	0.0012	NWMN_RS11835	1.2329	0.0467	NWMN_RS06800	1.2350	0.0047
504							NWMN_RS03930	1.7482	0.0034	NWMN_RS11475	1.2365	0.0002	NWMN_RS13970	1.2372	0.0000
505							NWMN_RS09605	1.7630	0.0188	NWMN_RS04360	1.2626	0.0355	NWMN_RS13510	1.2447	0.0009
506							NWMN_RS02320	1.7779	0.0299	NWMN_RS02240	1.2654	0.0034	NWMN_RS04120	1.2500	0.0002
507							NWMN_RS07480	1.8227	0.0293	NWMN_RS02335	1.2729	0.0260	NWMN_RS13965	1.2534	0.0000
508							NWMN_RS07495	1.8244	0.0001	NWMN_RS14640	1.2731	0.0434	NWMN_RS14655	1.2557	0.0283
509							NWMN_RS14760	1.8247	0.0191	NWMN_RS14070	1.2746	0.0344	NWMN_RS13255	1.2625	0.0052
510							NWMN_RS13345	1.8305	0.0000	NWMN_RS03520	1.2840	0.0079	NWMN_RS06540	1.2725	0.0007
511							NWMN_RS14240	1.8405	0.0000	NWMN_RS08995	1.2900	0.0075	NWMN_RS14240	1.2732	0.0068
512							NWMN_RS14870	1.8497	0.0020	NWMN_RS07010	1.2944	0.0000	NWMN_RS11800	1.2886	0.0022
513							NWMN_RS15000	1.8685	0.0005	NWMN_RS03695	1.2961	0.0029	NWMN_RS11900	1.2967	0.0059
514							NWMN_RS02315	1.8872	0.0065	NWMN_RS00180	1.3096	0.0035	NWMN_RS07010	1.3137	0.0000
515							NWMN_RS10660	1.8934	0.0001	NWMN_RS00745	1.3112	0.0036	NWMN_RS11460	1.3151	0.0006
516							NWMN_RS12090	1.8959	0.0020	NWMN_RS13335	1.3181	0.0168	NWMN_RS14950	1.3160	0.0031
517							NWMN_RS11480	1.9270	0.0013	NWMN_RS02840	1.3227	0.0002	NWMN_RS08800	1.3200	0.0405
518							NWMN_RS04360	1.9462	0.0005	NWMN_RS04685	1.3310	0.0002	NWMN_RS09165	1.3202	0.0018
519							NWMN_RS07490	1.9550	0.0001	NWMN_RS14340	1.3347	0.0004	NWMN_RS02840	1.3225	0.0003
520							NWMN_RS13645	1.9634	0.0005	NWMN_RS06105	1.3452	0.0339	NWMN_RS14575	1.3300	0.0161
521							NWMN_RS11130	1.9666	0.0000	NWMN_RS11120	1.3477	0.0089	NWMN_RS05135	1.3321	0.0087
522							NWMN_RS04280	1.9727	0.0018	NWMN_RS13515	1.3485	0.0144	NWMN_RS14395	1.3324	0.0000
523							NWMN_RS12085	1.9825	0.0011	NWMN_RS00355	1.3532	0.0406	NWMN_RS14945	1.3331	0.0036
524							NWMN_RS03695	2.0187	0.0000	NWMN_RS12750	1.3585	0.0059	NWMN_RS10425	1.3334	0.0012

TABLE S4A continued

	Time 2 min			Time 5 min			Time 10 min			Time 20 min			Time 30 min		
#	gene ID	log2 Fold Change	p adj. value	gene ID	log2 Fold Change	p adj. value	gene ID	log2 Fold Change	p adj. value	gene ID	log2 Fold Change	p adj. value	gene ID	log2 Fold Change	p adj. value
525							NWMN_RS13650	2.0387	0.0081	NWMN_RS02325	1.3881	0.0002	NWMN_RS06105	1.3339	0.0374
526							NWMN_RS11825	2.0634	0.0001	NWMN_RS13555	1.3924	0.0174	NWMN_RS14215	1.3400	0.0002
527							NWMN_RS14885	2.0669	0.0002	NWMN_RS06100	1.3983	0.0127	NWMN_RS14660	1.3506	0.0010
528							NWMN_RS14360	2.0815	0.0116	NWMN_RS00175	1.4051	0.0009	NWMN_RS01830	1.3571	0.0102
529							NWMN_RS04180	2.1037	0.0000	NWMN_RS10770	1.4083	0.0054	NWMN_RS14645	1.3573	0.0093
530							NWMN_RS13190	2.1188	0.0000	NWMN_RS05880	1.4108	0.0219	NWMN_RS10955	1.3586	0.0366
531							NWMN_RS13555	2.1280	0.0001	NWMN_RS00265	1.4221	0.0399	NWMN_RS09295	1.3612	0.0062
532							NWMN_RS00290	2.1447	0.0006	NWMN_RS00750	1.4340	0.0000	NWMN_RS13775	1.3680	0.0003
533							NWMN_RS00280	2.1530	0.0000	NWMN_RS04210	1.4371	0.0329	NWMN_RS01440	1.3698	0.0308
534							NWMN_RS13185	2.1535	0.0000	NWMN_RS12850	1.4409	0.0015	NWMN_RS01350	1.3777	0.0436
535							NWMN_RS09610	2.1618	0.0007	NWMN_RS14005	1.4442	0.0002	NWMN_RS10865	1.3806	0.0319
536							NWMN_RS01830	2.2064	0.0000	NWMN_RS12865	1.4451	0.0012	NWMN_RS00495	1.3828	0.0323
537							NWMN_RS12880	2.2095	0.0000	NWMN_RS13510	1.4596	0.0000	NWMN_RS05140	1.3839	0.0205
538							NWMN_RS06110	2.2110	0.0001	NWMN_RS14145	1.4639	0.0244	NWMN_RS12865	1.3889	0.0026
539							NWMN_RS13560	2.2194	0.0002	NWMN_RS04760	1.4729	0.0000	NWMN_RS03785	1.4081	0.0000
540							NWMN_RS04270	2.2370	0.0037	NWMN_RS00495	1.4851	0.0159	NWMN_RS14400	1.4115	0.0011
541							NWMN_RS02060	2.2946	0.0008	NWMN_RS11825	1.4887	0.0088	NWMN_RS08350	1.4212	0.0010
542							NWMN_RS14145	2.3655	0.0001	NWMN_RS13660	1.4924	0.0070	NWMN_RS14650	1.4322	0.0158
543							NWMN_RS14275	2.3705	0.0000	NWMN_RS14010	1.4939	0.0039	NWMN_RS11480	1.4368	0.0342
544							NWMN_RS14160	2.3906	0.0029	NWMN_RS07000	1.4966	0.0000	NWMN_RS00475	1.4458	0.0178
545							NWMN_RS13165	2.4014	0.0183	NWMN_RS11130	1.5085	0.0004	NWMN_RS11110	1.4475	0.0005
546							NWMN_RS13195	2.5070	0.0000	NWMN_RS01645	1.5200	0.0027	NWMN_RS07300	1.4493	0.0039
547							NWMN_RS00635	2.5671	0.0001	NWMN_RS12880	1.5378	0.0032	NWMN_RS07205	1.4522	0.0000
548							NWMN_RS13200	2.5936	0.0000	NWMN_RS02465	1.5463	0.0000	NWMN_RS13350	1.4568	0.0308
549							NWMN_RS14150	2.6104	0.0001	NWMN_RS14155	1.5465	0.0406	NWMN_RS02335	1.4578	0.0108
550							NWMN_RS00275	2.6107	0.0000	NWMN_RS07590	1.5485	0.0001	NWMN_RS01180	1.4590	0.0016
551							NWMN_RS00885	2.6338	0.0036	NWMN_RS00400	1.5518	0.0000	NWMN_RS06300	1.4611	0.0428
552							NWMN_RS00865	2.6836	0.0000	NWMN_RS04160	1.5608	0.0004	NWMN_RS01245	1.4655	0.0113
553							NWMN_RS00455	2.7283	0.0000	NWMN_RS11125	1.5613	0.0068	NWMN_RS04115	1.4767	0.0000
554							NWMN_RS13205	2.7322	0.0000	NWMN_RS13645	1.5641	0.0082	NWMN_RS14640	1.4769	0.0196
555							NWMN_RS13210	2.8057	0.0000	NWMN_RS07005	1.5676	0.0000	NWMN_RS06115	1.4789	0.0024
556							NWMN_RS14155	2.8786	0.0000	NWMN_RS14015	1.5764	0.0024	NWMN_RS00745	1.4954	0.0008
557							NWMN_RS13230	3.1760	0.0001	NWMN_RS14150	1.5783	0.0249	NWMN_RS11570	1.4978	0.0035
558							NWMN_RS13215	3.2838	0.0004	NWMN_RS10855	1.5786	0.0005	NWMN_RS03775	1.5124	0.0003
559							NWMN_RS13225	4.4772	0.0000	NWMN_RS14275	1.5793	0.0054	NWMN_RS07005	1.5325	0.0000
560							NWMN_RS13220	4.7351	0.0000	NWMN_RS12080	1.5863	0.0084	NWMN_RS05620	1.5364	0.0414
561										NWMN_RS04205	1.5913	0.0158	NWMN_RS13505	1.5512	0.0000
562										NWMN_RS14765	1.5936	0.0425	NWMN_RS10395	1.5615	0.0209
563										NWMN_RS07895	1.6042	0.0127	NWMN_RS05615	1.5705	0.0389
564										NWMN_RS14755	1.6052	0.0018	NWMN_RS15885	1.5707	0.0161
565										NWMN_RS00170	1.6087	0.0001	NWMN_RS13520	1.5773	0.0001
566										NWMN_RS09165	1.6105	0.0001	NWMN_RS00180	1.5806	0.0003
567										NWMN_RS10865	1.6120	0.0091	NWMN_RS13180	1.5836	0.0018
568										NWMN_RS14105	1.6199	0.0000	NWMN_RS10855	1.5878	0.0005
569										NWMN_RS15435	1.6286	0.0342	NWMN_RS05610	1.5887	0.0190
570										NWMN_RS12090	1.6286	0.0092	NWMN_RS00530	1.5901	0.0114
571										NWMN_RS09255	1.6298	0.0007	NWMN_RS05590	1.5918	0.0151
572										NWMN_RS02460	1.6670	0.0001	NWMN_RS07070	1.5942	0.0078
573										NWMN_RS04200	1.6689	0.0120	NWMN_RS01910	1.5965	0.0049
574										NWMN_RS03785	1.6696	0.0000	NWMN_RS13335	1.6000	0.0030
575										NWMN_RS13350	1.6818	0.0095	NWMN_RS02015	1.6009	0.0028
576										NWMN_RS01905	1.6999	0.0257	NWMN_RS04280	1.6138	0.0137
577										NWMN_RS01025	1.7018	0.0120	NWMN_RS07000	1.6164	0.0000
578										NWMN_RS12085	1.7111	0.0057	NWMN_RS05835	1.6219	0.0314
579										NWMN_RS07610	1.7179	0.0117	NWMN_RS11125	1.6253	0.0049
580										NWMN_RS13355	1.7356	0.0057	NWMN_RS00460	1.6479	0.0000
581										NWMN_RS14240	1.7388	0.0001	NWMN_RS13515	1.6490	0.0023
582										NWMN_RS03775	1.7391	0.0000	NWMN_RS00255	1.6588	0.0389
583										NWMN_RS00460	1.7604	0.0000	NWMN_RS15435	1.6646	0.0352

TABLE S4A continued

	Time 2 min			Time 5 min			Time 10 min			Time 20 min			Time 30 min		
#	gene ID	log2 Fold Change	p adj. value	gene ID	log2 Fold Change	p adj. value	gene ID	log2 Fold Change	p adj. value	gene ID	log2 Fold Change	p adj. value	gene ID	log2 Fold Change	p adj. value
584										NWMN_RS01830	1.8071	0.0003	NWMN_RS05830	1.6652	0.0367
585										NWMN_RS13180	1.8236	0.0002	NWMN_RS13795	1.6948	0.0000
586										NWMN_RS03780	1.8267	0.0000	NWMN_RS14755	1.6954	0.0013
587										NWMN_RS13650	1.8417	0.0180	NWMN_RS07590	1.7491	0.0000
588										NWMN_RS00410	1.8695	0.0201	NWMN_RS04695	1.7524	0.0114
589										NWMN_RS00440	1.8795	0.0014	NWMN_RS04760	1.7565	0.0000
590										NWMN_RS02200	1.8862	0.0007	NWMN_RS03035	1.7927	0.0000
591										NWMN_RS14885	1.8947	0.0009	NWMN_RS03300	1.8192	0.0339
592										NWMN_RS07195	1.9205	0.0002	NWMN_RS15365	1.8306	0.0341
593										NWMN_RS04280	1.9251	0.0024	NWMN_RS00275	1.8331	0.0003
594										NWMN_RS10910	1.9463	0.0259	NWMN_RS04270	1.8374	0.0319
595										NWMN_RS00280	1.9508	0.0002	NWMN_RS10165	1.8504	0.0139
596										NWMN_RS02060	1.9639	0.0051	NWMN_RS02465	1.8696	0.0000
597										NWMN_RS00290	1.9676	0.0020	NWMN_RS06110	1.8761	0.0032
598										NWMN_RS05130	2.0230	0.0043	NWMN_RS09255	1.8796	0.0001
599										NWMN_RS13345	2.0408	0.0000	NWMN_RS05625	1.8806	0.0102
600										NWMN_RS00890	2.0448	0.0348	NWMN_RS13355	1.9129	0.0021
601										NWMN_RS14760	2.0509	0.0076	NWMN_RS13345	1.9239	0.0000
602										NWMN_RS06115	2.1032	0.0000	NWMN_RS02200	1.9317	0.0005
603										NWMN_RS13190	2.1036	0.0000	NWMN_RS02460	1.9518	0.0000
604										NWMN_RS07320	2.1293	0.0014	NWMN_RS00175	1.9882	0.0000
605										NWMN_RS04695	2.1454	0.0013	NWMN_RS02285	2.0223	0.0042
606										NWMN_RS13520	2.2093	0.0000	NWMN_RS13190	2.0558	0.0000
607										NWMN_RS13185	2.4000	0.0000	NWMN_RS14760	2.0705	0.0085
608										NWMN_RS13195	2.4341	0.0000	NWMN_RS14765	2.0822	0.0054
609										NWMN_RS00275	2.4573	0.0000	NWMN_RS00170	2.0879	0.0000
610										NWMN_RS03300	2.4892	0.0023	NWMN_RS03780	2.1317	0.0000
611										NWMN_RS00530	2.6371	0.0000	NWMN_RS13185	2.2177	0.0000
612										NWMN_RS13165	2.6807	0.0072	NWMN_RS00885	2.2454	0.0155
613										NWMN_RS13200	2.6835	0.0000	NWMN_RS13215	2.3025	0.0190
614										NWMN_RS00455	2.7307	0.0000	NWMN_RS13195	2.3111	0.0000
615										NWMN_RS06110	2.7720	0.0000	NWMN_RS07320	2.3325	0.0005
616										NWMN_RS13210	2.8151	0.0000	NWMN_RS05130	2.3911	0.0005
617										NWMN_RS13205	2.8502	0.0000	NWMN_RS00455	2.4134	0.0000
618										NWMN_RS00885	2.8575	0.0014	NWMN_RS13200	2.4842	0.0000
619										NWMN_RS13230	3.1702	0.0002	NWMN_RS13230	2.5297	0.0037
620										NWMN_RS00865	3.1778	0.0000	NWMN_RS13210	2.5370	0.0000
621										NWMN_RS13215	3.3026	0.0004	NWMN_RS07195	2.5490	0.0000
622										NWMN_RS13225	4.4918	0.0000	NWMN_RS13205	2.6473	0.0000
623										NWMN_RS13220	4.7702	0.0000	NWMN_RS00865	3.3947	0.0000
624													NWMN_RS13225	3.4587	0.0002
625													NWMN_RS13220	3.7578	0.0001

TABLE S4B Significantly differentially expressed genes in *Staphylococcus aureus* SH1000

De novo sequenced genome of *S. aureus* SH1000 deposited under Bioproject Accession number PRJNA769253 was used for the differential expression analysis with the DESeq2 R package. Gene IDs were obtained from the RAST annotation pipeline, whereas locus tags were assigned by Prokaryotic genome annotation pipeline (PGAP) during deposition in the NCBI database. Genes with log2-fold changes (L2FC) ± 0.58 with p < 0.05 corresponding to 1.5-fold upregulation or downregulation are listed below.

<table>
<tr><th></th><th colspan="4">Time 2 min</th><th colspan="4">Time 5 min</th><th colspan="4">Time 10 min</th><th colspan="4">Time 20 min</th><th colspan="4">Time 30 min</th></tr>
<tr><th>#</th><th>Gene ID</th><th>Locus Tag</th><th>log2 Fold Change</th><th>p adj. value</th><th>Gene ID</th><th>Locus Tag</th><th>log2 Fold Change</th><th>p adj. value</th><th>Gene ID</th><th>Locus Tag</th><th>log2 Fold Change</th><th>p adj. value</th><th>Gene ID</th><th>Locus Tag</th><th>log2 Fold Change</th><th>p adj. value</th><th>Gene ID</th><th>Locus Tag</th><th>log2 Fold Change</th><th>p adj. value</th></tr>
<tr><td>1</td><td>fig|1280.19811.peg.1793</td><td>LGY17_09320</td><td>-5.6522</td><td>0.0064</td><td>fig|1280.19811.peg.1797</td><td>LGY17_09340</td><td>-5.5499</td><td>0.0163</td><td>fig|1280.19811.peg.1626</td><td>LGY17_08425</td><td>-2.6864</td><td>0.0024</td><td>fig|1280.19811.peg.1484</td><td>LGY17_07675</td><td>-2.1983</td><td>0.0002</td><td>fig|1280.19811.peg.1992</td><td>LGY17_10330</td><td>-2.1966</td><td>0.0367</td></tr>
<tr><td>2</td><td>fig|1280.19811.peg.1797</td><td>LGY17_09340</td><td>-5.5323</td><td>0.0248</td><td>fig|1280.19811.peg.1798</td><td>LGY17_09345</td><td>-5.1951</td><td>0.0319</td><td>fig|1280.19811.peg.1484</td><td>LGY17_07675</td><td>-2.4662</td><td>0.0000</td><td>fig|1280.19811.peg.1626</td><td>LGY17_08425</td><td>-2.1024</td><td>0.0332</td><td>fig|1280.19811.peg.1823</td><td>LGY17_09465</td><td>-1.9308</td><td>0.0075</td></tr>
<tr><td>3</td><td>fig|1280.19811.peg.396</td><td>LGY17_02095</td><td>-5.4840</td><td>0.0003</td><td>fig|1280.19811.peg.396</td><td>LGY17_02095</td><td>-4.7630</td><td>0.0013</td><td>fig|1280.19811.peg.321</td><td>LGY17_01720</td><td>-2.2643</td><td>0.0000</td><td>fig|1280.19811.peg.2015</td><td>LGY17_10450</td><td>-2.0068</td><td>0.0456</td><td>fig|1280.19811.peg.1484</td><td>LGY17_07675</td><td>-1.8326</td><td>0.0036</td></tr>
<tr><td>4</td><td>fig|1280.19811.peg.1794</td><td>LGY17_09325</td><td>-5.3358</td><td>0.0049</td><td>fig|1280.19811.peg.1794</td><td>LGY17_09325</td><td>-4.7241</td><td>0.0130</td><td>fig|1280.19811.peg.2319</td><td>LGY17_12305</td><td>-2.1750</td><td>0.0262</td><td>fig|1280.19811.peg.321</td><td>LGY17_01720</td><td>-1.9542</td><td>0.0004</td><td>fig|1280.19811.peg.321</td><td>LGY17_01720</td><td>-1.7118</td><td>0.0031</td></tr>
<tr><td>5</td><td>fig|1280.19811.peg.395</td><td>LGY17_02090</td><td>-4.4600</td><td>0.0048</td><td>fig|1280.19811.peg.1793</td><td>LGY17_09320</td><td>-4.4738</td><td>0.0416</td><td>fig|1280.19811.peg.1298</td><td>LGY17_06710</td><td>-1.6857</td><td>0.0129</td><td>fig|1280.19811.peg.1622</td><td>LGY17_08405</td><td>-1.8880</td><td>0.0332</td><td>fig|1280.19811.peg.2360</td><td>LGY17_12575</td><td>-1.5046</td><td>0.0053</td></tr>
<tr><td>6</td><td>fig|1280.19811.peg.1795</td><td>LGY17_09330</td><td>-4.4230</td><td>0.0251</td><td>fig|1280.19811.peg.395</td><td>LGY17_02090</td><td>-4.3035</td><td>0.0047</td><td>fig|1280.19811.peg.1622</td><td>LGY17_08405</td><td>-1.6735</td><td>0.0306</td><td>fig|1280.19811.peg.161</td><td>LGY17_00885</td><td>-1.8855</td><td>0.0009</td><td>fig|1280.19811.peg.1561</td><td>LGY17_08065</td><td>-1.4691</td><td>0.0189</td></tr>
<tr><td>7</td><td>fig|1280.19811.peg.1715</td><td>LGY17_08890</td><td>-3.9301</td><td>0.0041</td><td>fig|1280.19811.peg.1795</td><td>LGY17_09330</td><td>-4.0780</td><td>0.0361</td><td>fig|1280.19811.peg.161</td><td>LGY17_00885</td><td>-1.5096</td><td>0.0127</td><td>fig|1280.19811.peg.160</td><td>LGY17_00880</td><td>-1.7058</td><td>0.0032</td><td>fig|1280.19811.peg.161</td><td>LGY17_00885</td><td>-1.4366</td><td>0.0228</td></tr>
<tr><td>8</td><td>fig|1280.19811.peg.1512</td><td>LGY17_07820</td><td>-3.5951</td><td>0.0382</td><td>fig|1280.19811.peg.1796</td><td>LGY17_09335</td><td>-3.7581</td><td>0.0416</td><td>fig|1280.19811.peg.1590</td><td>LGY17_08245</td><td>-1.4780</td><td>0.0435</td><td>fig|1280.19811.peg.1590</td><td>LGY17_08245</td><td>-1.6790</td><td>0.0209</td><td>fig|1280.19811.peg.2356</td><td>LGY17_12550</td><td>-1.3821</td><td>0.0350</td></tr>
<tr><td>9</td><td>fig|1280.19811.peg.2102</td><td>LGY17_10970</td><td>-2.9868</td><td>0.0416</td><td>fig|1280.19811.peg.1513</td><td>LGY17_07825</td><td>-2.9991</td><td>0.0298</td><td>fig|1280.19811.peg.1561</td><td>LGY17_08065</td><td>-1.4470</td><td>0.0195</td><td>fig|1280.19811.peg.1561</td><td>LGY17_08065</td><td>-1.4266</td><td>0.0232</td><td>fig|1280.19811.peg.2357</td><td>LGY17_12560</td><td>-1.3291</td><td>0.0087</td></tr>
<tr><td>10</td><td>fig|1280.19811.peg.2101</td><td>LGY17_10965</td><td>-2.9404</td><td>0.0191</td><td>fig|1280.19811.peg.2319</td><td>LGY17_12305</td><td>-2.8925</td><td>0.0012</td><td>fig|1280.19811.peg.1623</td><td>LGY17_08410</td><td>-1.3866</td><td>0.0004</td><td>fig|1280.19811.peg.1891</td><td>LGY17_09800</td><td>-1.2875</td><td>0.0116</td><td>fig|1280.19811.peg.2298</td><td>LGY17_12185</td><td>-1.1193</td><td>0.0294</td></tr>
<tr><td>11</td><td>fig|1280.19811.peg.1589</td><td>LGY17_08240</td><td>-2.7189</td><td>0.0226</td><td>fig|1280.19811.peg.2102</td><td>LGY17_10970</td><td>-2.8329</td><td>0.0447</td><td>fig|1280.19811.peg.2382</td><td>LGY17_12680</td><td>-1.3850</td><td>0.0441</td><td>fig|1280.19811.peg.2360</td><td>LGY17_12575</td><td>-1.2607</td><td>0.0158</td><td>fig|1280.19811.peg.2361</td><td>LGY17_12580</td><td>-1.0652</td><td>0.0216</td></tr>
<tr><td>12</td><td>fig|1280.19811.peg.1590</td><td>LGY17_08245</td><td>-1.6787</td><td>0.0283</td><td>fig|1280.19811.peg.1589</td><td>LGY17_08240</td><td>-2.4900</td><td>0.0353</td><td>fig|1280.19811.peg.192</td><td>LGY17_01045</td><td>-1.3338</td><td>0.0003</td><td>fig|1280.19811.peg.174</td><td>LGY17_00955</td><td>-1.1593</td><td>0.0387</td><td>fig|1280.19811.peg.667</td><td>LGY17_03470</td><td>-0.9608</td><td>0.0000</td></tr>
<tr><td>13</td><td>fig|1280.19811.peg.1687</td><td>LGY17_08750</td><td>-1.5246</td><td>0.0084</td><td>fig|1280.19811.peg.1645</td><td>LGY17_08520</td><td>-2.3964</td><td>0.0498</td><td>fig|1280.19811.peg.160</td><td>LGY17_00880</td><td>-1.3204</td><td>0.0352</td><td>fig|1280.19811.peg.2357</td><td>LGY17_12560</td><td>-1.0430</td><td>0.0374</td><td>fig|1280.19811.peg.668</td><td>LGY17_03475</td><td>-0.8544</td><td>0.0012</td></tr>
<tr><td>14</td><td>fig|1280.19811.peg.1633</td><td>LGY17_08460</td><td>-1.3035</td><td>0.0428</td><td>fig|1280.19811.peg.842</td><td>LGY17_04360</td><td>-2.1990</td><td>0.0416</td><td>fig|1280.19811.peg.1895</td><td>LGY17_09820</td><td>-1.2523</td><td>0.0016</td><td>fig|1280.19811.peg.406</td><td>LGY17_02145</td><td>-1.0082</td><td>0.0000</td><td>fig|1280.19811.peg.727</td><td>LGY17_03775</td><td>-0.8525</td><td>0.0497</td></tr>
<tr><td>15</td><td>fig|1280.19811.peg.885</td><td>LGY17_04570</td><td>-0.7386</td><td>0.0079</td><td>fig|1280.19811.peg.2224</td><td>LGY17_11785</td><td>-2.1937</td><td>0.0331</td><td>fig|1280.19811.peg.2358</td><td>LGY17_12565</td><td>-1.1723</td><td>0.0376</td><td>fig|1280.19811.peg.626</td><td>LGY17_03270</td><td>-1.0022</td><td>0.0117</td><td>fig|1280.19811.peg.406</td><td>LGY17_02145</td><td>-0.8507</td><td>0.0008</td></tr>
<tr><td>16</td><td>fig|1280.19811.peg.2023</td><td>LGY17_10495</td><td>-0.6782</td><td>0.0079</td><td>fig|1280.19811.peg.2320</td><td>LGY17_12310</td><td>-2.0829</td><td>0.0104</td><td>fig|1280.19811.peg.1297</td><td>LGY17_06705</td><td>-1.1700</td><td>0.0303</td><td>fig|1280.19811.peg.1946</td><td>LGY17_10090</td><td>-0.9875</td><td>0.0314</td><td>fig|1280.19811.peg.2005</td><td>LGY17_10405</td><td>-0.8464</td><td>0.0216</td></tr>
<tr><td>17</td><td>fig|1280.19811.peg.774</td><td>LGY17_04000</td><td>0.8375</td><td>0.0351</td><td>fig|1280.19811.peg.1484</td><td>LGY17_07675</td><td>-1.9038</td><td>0.0024</td><td>fig|1280.19811.peg.667</td><td>LGY17_03470</td><td>-1.1513</td><td>0.0000</td><td>fig|1280.19811.peg.667</td><td>LGY17_03470</td><td>-0.9865</td><td>0.0000</td><td>fig|1280.19811.peg.573</td><td>LGY17_03000</td><td>-0.8160</td><td>0.0014</td></tr>
<tr><td>18</td><td>fig|1280.19811.peg.533</td><td>LGY17_02805</td><td>1.0497</td><td>0.0213</td><td>fig|1280.19811.peg.321</td><td>LGY17_01720</td><td>-1.7379</td><td>0.0028</td><td>fig|1280.19811.peg.1163</td><td>LGY17_06005</td><td>-1.0497</td><td>0.0026</td><td>fig|1280.19811.peg.1623</td><td>LGY17_08410</td><td>-0.9826</td><td>0.0281</td><td>fig|1280.19811.peg.419</td><td>LGY17_02200</td><td>-0.7708</td><td>0.0304</td></tr>
<tr><td>19</td><td></td><td></td><td></td><td></td><td>fig|1280.19811.peg.1590</td><td>LGY17_08245</td><td>-1.7146</td><td>0.0161</td><td>fig|1280.19811.peg.2360</td><td>LGY17_12575</td><td>-1.0215</td><td>0.0303</td><td>fig|1280.19811.peg.2124</td><td>LGY17_11240</td><td>-0.9646</td><td>0.0316</td><td>fig|1280.19811.peg.669</td><td>LGY17_03480</td><td>-0.7442</td><td>0.0158</td></tr>
<tr><td>20</td><td></td><td></td><td></td><td></td><td>fig|1280.19811.peg.2340</td><td>LGY17_12465</td><td>-1.2253</td><td>0.0428</td><td>fig|1280.19811.peg.406</td><td>LGY17_02145</td><td>-1.0210</td><td>0.0000</td><td>fig|1280.19811.peg.192</td><td>LGY17_01045</td><td>-0.9228</td><td>0.0314</td><td>fig|1280.19811.peg.1027</td><td>LGY17_05305</td><td>-0.7427</td><td>0.0163</td></tr>
<tr><td>21</td><td></td><td></td><td></td><td></td><td>fig|1280.19811.peg.1868</td><td>LGY17_09690</td><td>-1.1370</td><td>0.0355</td><td>fig|1280.19811.peg.668</td><td>LGY17_03475</td><td>-1.0015</td><td>0.0000</td><td>fig|1280.19811.peg.668</td><td>LGY17_03475</td><td>-0.9061</td><td>0.0004</td><td>fig|1280.19811.peg.1814</td><td>LGY17_09420</td><td>-0.7190</td><td>0.0417</td></tr>
<tr><td>22</td><td></td><td></td><td></td><td></td><td>fig|1280.19811.peg.747</td><td>LGY17_03875</td><td>-1.0573</td><td>0.0310</td><td>fig|1280.19811.peg.2411</td><td>LGY17_12825</td><td>-0.9356</td><td>0.0114</td><td>fig|1280.19811.peg.1153</td><td>LGY17_05955</td><td>-0.9004</td><td>0.0296</td><td>fig|1280.19811.peg.1041</td><td>LGY17_05375</td><td>-0.7156</td><td>0.0007</td></tr>
<tr><td>23</td><td></td><td></td><td></td><td></td><td>fig|1280.19811.peg.184</td><td>LGY17_01005</td><td>-0.8555</td><td>0.0335</td><td>fig|1280.19811.peg.615</td><td>LGY17_03215</td><td>-0.9224</td><td>0.0003</td><td>fig|1280.19811.peg.1027</td><td>LGY17_05305</td><td>-0.8839</td><td>0.0015</td><td>fig|1280.19811.peg.924</td><td>LGY17_04770</td><td>-0.7023</td><td>0.0175</td></tr>
<tr><td>24</td><td></td><td></td><td></td><td></td><td>fig|1280.19811.peg.789</td><td>LGY17_04085</td><td>-0.8530</td><td>0.0155</td><td>fig|1280.19811.peg.2357</td><td>LGY17_12560</td><td>-0.9093</td><td>0.0421</td><td>fig|1280.19811.peg.419</td><td>LGY17_02200</td><td>-0.8690</td><td>0.0094</td><td>fig|1280.19811.peg.1042</td><td>LGY17_05380</td><td>-0.6234</td><td>0.0290</td></tr>
<tr><td>25</td><td></td><td></td><td></td><td></td><td>fig|1280.19811.peg.852</td><td>LGY17_04410</td><td>-0.7922</td><td>0.0161</td><td>fig|1280.19811.peg.1686</td><td>LGY17_08745</td><td>-0.9064</td><td>0.0257</td><td>fig|1280.19811.peg.669</td><td>LGY17_03480</td><td>-0.8417</td><td>0.0037</td><td>fig|1280.19811.peg.616</td><td>LGY17_03220</td><td>-0.6230</td><td>0.0420</td></tr>
<tr><td>26</td><td></td><td></td><td></td><td></td><td>fig|1280.19811.peg.2411</td><td>LGY17_12825</td><td>-0.7804</td><td>0.0333</td><td>fig|1280.19811.peg.2470</td><td>LGY17_13135</td><td>-0.8995</td><td>0.0415</td><td>fig|1280.19811.peg.1943</td><td>LGY17_10075</td><td>-0.7977</td><td>0.0317</td><td>fig|1280.19811.peg.2081</td><td>LGY17_10860</td><td>-0.6018</td><td>0.0349</td></tr>
<tr><td>27</td><td></td><td></td><td></td><td></td><td>fig|1280.19811.peg.2023</td><td>LGY17_10495</td><td>-0.7655</td><td>0.0012</td><td>fig|1280.19811.peg.1488</td><td>LGY17_07700</td><td>-0.8970</td><td>0.0110</td><td>fig|1280.19811.peg.1041</td><td>LGY17_05375</td><td>-0.7806</td><td>0.0001</td><td>fig|1280.19811.peg.2136</td><td>LGY17_11305</td><td>-0.5983</td><td>0.0242</td></tr>
<tr><td>28</td><td></td><td></td><td></td><td></td><td>fig|1280.19811.peg.1684</td><td>LGY17_08735</td><td>-0.7575</td><td>0.0092</td><td>fig|1280.19811.peg.1917</td><td>LGY17_09935</td><td>-0.8652</td><td>0.0126</td><td>fig|1280.19811.peg.1453</td><td>LGY17_07510</td><td>-0.7799</td><td>0.0012</td><td>fig|1280.19811.peg.823</td><td>LGY17_04260</td><td>-0.5873</td><td>0.0069</td></tr>
<tr><td>29</td><td></td><td></td><td></td><td></td><td>fig|1280.19811.peg.2005</td><td>LGY17_10405</td><td>-0.7224</td><td>0.0278</td><td>fig|1280.19811.peg.419</td><td>LGY17_02200</td><td>-0.8578</td><td>0.0098</td><td>fig|1280.19811.peg.66</td><td>LGY17_00405</td><td>-0.7700</td><td>0.0287</td><td>fig|1280.19811.peg.996</td><td>LGY17_05145</td><td>0.6389</td><td>0.0156</td></tr>
<tr><td>30</td><td></td><td></td><td></td><td></td><td>fig|1280.19811.peg.1667</td><td>LGY17_08645</td><td>-0.6365</td><td>0.0078</td><td>fig|1280.19811.peg.669</td><td>LGY17_03480</td><td>-0.8399</td><td>0.0031</td><td>fig|1280.19811.peg.65</td><td>LGY17_00400</td><td>-0.7667</td><td>0.0167</td><td>fig|1280.19811.peg.1920</td><td>LGY17_09950</td><td>0.6954</td><td>0.0426</td></tr>
<tr><td>31</td><td></td><td></td><td></td><td></td><td>fig|1280.19811.peg.616</td><td>LGY17_03220</td><td>-0.6298</td><td>0.0412</td><td>fig|1280.19811.peg.1684</td><td>LGY17_08735</td><td>-0.8313</td><td>0.0031</td><td>fig|1280.19811.peg.101</td><td>LGY17_00575</td><td>-0.7662</td><td>0.0009</td><td>fig|1280.19811.peg.2147</td><td>LGY17_11365</td><td>0.7591</td><td>0.0386</td></tr>
<tr><td>32</td><td></td><td></td><td></td><td></td><td>fig|1280.19811.peg.406</td><td>LGY17_02145</td><td>-0.5929</td><td>0.0139</td><td>fig|1280.19811.peg.2005</td><td>LGY17_10405</td><td>-0.7925</td><td>0.0114</td><td>fig|1280.19811.peg.1040</td><td>LGY17_05370</td><td>-0.7319</td><td>0.0000</td><td>fig|1280.19811.peg.1110</td><td>LGY17_05730</td><td>0.8145</td><td>0.0465</td></tr>
<tr><td>33</td><td></td><td></td><td></td><td></td><td>fig|1280.19811.peg.1347</td><td>LGY17_06965</td><td>0.6588</td><td>0.0377</td><td>fig|1280.19811.peg.2295</td><td>LGY17_12170</td><td>-0.7853</td><td>0.0039</td><td>fig|1280.19811.peg.2136</td><td>LGY17_11305</td><td>-0.7151</td><td>0.0031</td><td>fig|1280.19811.peg.1890</td><td>LGY17_09795</td><td>0.8339</td><td>0.0475</td></tr>
<tr><td>34</td><td></td><td></td><td></td><td></td><td>fig|1280.19811.peg.2385</td><td>LGY17_12695</td><td>0.6604</td><td>0.0086</td><td>fig|1280.19811.peg.789</td><td>LGY17_04085</td><td>-0.7830</td><td>0.0277</td><td>fig|1280.19811.peg.2284</td><td>LGY17_12115</td><td>-0.7050</td><td>0.0442</td><td>fig|1280.19811.peg.2385</td><td>LGY17_12695</td><td>0.8493</td><td>0.0028</td></tr>
<tr><td>35</td><td></td><td></td><td></td><td></td><td>fig|1280.19811.peg.189</td><td>LGY17_01030</td><td>0.7835</td><td>0.0428</td><td>fig|1280.19811.peg.492</td><td>LGY17_02590</td><td>-0.7717</td><td>0.0339</td><td>fig|1280.19811.peg.24</td><td>LGY17_00130</td><td>-0.6873</td><td>0.0046</td><td>fig|1280.19811.peg.158</td><td>LGY17_00870</td><td>0.8599</td><td>0.0191</td></tr>
<tr><td>36</td><td></td><td></td><td></td><td></td><td>fig|1280.19811.peg.2337</td><td>LGY17_12450</td><td>0.8511</td><td>0.0419</td><td>fig|1280.19811.peg.924</td><td>LGY17_04770</td><td>-0.7679</td><td>0.0049</td><td>fig|1280.19811.peg.2286</td><td>LGY17_12125</td><td>-0.6742</td><td>0.0004</td><td>fig|1280.19811.peg.1860</td><td>LGY17_09650</td><td>0.8742</td><td>0.0044</td></tr>
<tr><td>37</td><td></td><td></td><td></td><td></td><td>fig|1280.19811.peg.2053</td><td>LGY17_10660</td><td>0.9811</td><td>0.0076</td><td>fig|1280.19811.peg.2286</td><td>LGY17_12125</td><td>-0.7662</td><td>0.0000</td><td>fig|1280.19811.peg.615</td><td>LGY17_03215</td><td>-0.6452</td><td>0.0397</td><td>fig|1280.19811.peg.1839</td><td>LGY17_09540</td><td>0.9260</td><td>0.0015</td></tr>
<tr><td>38</td><td></td><td></td><td></td><td></td><td>fig|1280.19811.peg.537</td><td>LGY17_02825</td><td>1.0215</td><td>0.0268</td><td>fig|1280.19811.peg.2094</td><td>LGY17_10930</td><td>-0.7514</td><td>0.0082</td><td>fig|1280.19811.peg.1667</td><td>LGY17_08645</td><td>-0.6380</td><td>0.0154</td><td>fig|1280.19811.peg.1148</td><td>LGY17_05925</td><td>0.9260</td><td>0.0367</td></tr>
<tr><td>39</td><td></td><td></td><td></td><td></td><td>fig|1280.19811.peg.367</td><td>LGY17_01955</td><td>1.0552</td><td>0.0160</td><td>fig|1280.19811.peg.1453</td><td>LGY17_07510</td><td>-0.7475</td><td>0.0006</td><td>fig|1280.19811.peg.659</td><td>LGY17_03430</td><td>-0.6374</td><td>0.0256</td><td>fig|1280.19811.peg.2384</td><td>LGY17_12690</td><td>0.9398</td><td>0.0137</td></tr>
<tr><td>40</td><td></td><td></td><td></td><td></td><td>fig|1280.19811.peg.368</td><td>LGY17_01960</td><td>1.0979</td><td>0.0059</td><td>fig|1280.19811.peg.2282</td><td>LGY17_12105</td><td>-0.7397</td><td>0.0208</td><td>fig|1280.19811.peg.1042</td><td>LGY17_05380</td><td>-0.6345</td><td>0.0182</td><td>fig|1280.19811.peg.1309</td><td>LGY17_06770</td><td>0.9404</td><td>0.0219</td></tr>
<tr><td>41</td><td></td><td></td><td></td><td></td><td>fig|1280.19811.peg.1258</td><td>LGY17_06500</td><td>1.1528</td><td>0.0442</td><td>fig|1280.19811.peg.2038</td><td>LGY17_10570</td><td>-0.7362</td><td>0.0242</td><td>fig|1280.19811.peg.616</td><td>LGY17_03220</td><td>-0.6240</td><td>0.0374</td><td>fig|1280.19811.peg.1452</td><td>LGY17_07505</td><td>0.9494</td><td>0.0228</td></tr>
<tr><td>42</td><td></td><td></td><td></td><td></td><td>fig|1280.19811.peg.365</td><td>LGY17_01945</td><td>1.1656</td><td>0.0237</td><td>fig|1280.19811.peg.2136</td><td>LGY17_11305</td><td>-0.7063</td><td>0.0021</td><td>fig|1280.19811.peg.924</td><td>LGY17_04770</td><td>-0.6118</td><td>0.0456</td><td>fig|1280.19811.peg.1024</td><td>LGY17_05290</td><td>0.9545</td><td>0.0094</td></tr>
<tr><td>43</td><td></td><td></td><td></td><td></td><td>fig|1280.19811.peg.1858</td><td>NA</td><td>1.2164</td><td>0.0098</td><td>fig|1280.19811.peg.1667</td><td>LGY17_08645</td><td>-0.6964</td><td>0.0026</td><td>fig|1280.19811.peg.212</td><td>LGY17_01145</td><td>-0.6058</td><td>0.0392</td><td>fig|1280.19811.peg.2386</td><td>LGY17_12700</td><td>0.9595</td><td>0.0139</td></tr>
<tr><td>44</td><td></td><td></td><td></td><td></td><td>fig|1280.19811.peg.121</td><td>LGY17_00685</td><td>1.2662</td><td>0.0241</td><td>fig|1280.19811.peg.2023</td><td>LGY17_10495</td><td>-0.6631</td><td>0.0093</td><td>fig|1280.19811.peg.2081</td><td>LGY17_10860</td><td>-0.6004</td><td>0.0262</td><td>fig|1280.19811.peg.788</td><td>LGY17_04080</td><td>0.9634</td><td>0.0226</td></tr>
<tr><td>45</td><td></td><td></td><td></td><td></td><td>fig|1280.19811.peg.536</td><td>LGY17_02820</td><td>1.3001</td><td>0.0031</td><td>fig|1280.19811.peg.616</td><td>LGY17_03220</td><td>-0.6082</td><td>0.0400</td><td>fig|1280.19811.peg.823</td><td>LGY17_04260</td><td>-0.5963</td><td>0.0048</td><td>fig|1280.19811.peg.717</td><td>LGY17_03725</td><td>0.9975</td><td>0.0001</td></tr>
<tr><td>46</td><td></td><td></td><td></td><td></td><td>fig|1280.19811.peg.533</td><td>LGY17_02805</td><td>1.3630</td><td>0.0004</td><td>fig|1280.19811.peg.823</td><td>LGY17_04260</td><td>-0.6017</td><td>0.0031</td><td>fig|1280.19811.peg.617</td><td>LGY17_03225</td><td>-0.5856</td><td>0.0477</td><td>fig|1280.19811.peg.533</td><td>LGY17_02805</td><td>1.1035</td><td>0.0214</td></tr>
<tr><td>47</td><td></td><td></td><td></td><td></td><td>fig|1280.19811.peg.534</td><td>LGY17_02810</td><td>1.3861</td><td>0.0106</td><td>fig|1280.19811.peg.2255</td><td>LGY17_11970</td><td>-0.5906</td><td>0.0278</td><td>fig|1280.19811.peg.1132</td><td>LGY17_05850</td><td>0.6109</td><td>0.0499</td><td>fig|1280.19811.peg.1458</td><td>LGY17_07545</td><td>1.1419</td><td>0.0108</td></tr>
<tr><td>48</td><td></td><td></td><td></td><td></td><td>fig|1280.19811.peg.535</td><td>LGY17_02815</td><td>1.4627</td><td>0.0004</td><td>fig|1280.19811.peg.440</td><td>LGY17_02305</td><td>0.6236</td><td>0.0096</td><td>fig|1280.19811.peg.1562</td><td>LGY17_08070</td><td>0.6335</td><td>0.0385</td><td>fig|1280.19811.peg.1098</td><td>LGY17_05670</td><td>1.1488</td><td>0.0216</td></tr>
<tr><td>49</td><td></td><td></td><td></td><td></td><td></td><td></td><td></td><td></td><td>fig|1280.19811.peg.1132</td><td>LGY17_05850</td><td>0.6463</td><td>0.0254</td><td>fig|1280.19811.peg.1997</td><td>LGY17_10365</td><td>0.6774</td><td>0.0350</td><td>fig|1280.19811.peg.1425</td><td>LGY17_07350</td><td>1.1679</td><td>0.0059</td></tr>
<tr><td>50</td><td></td><td></td><td></td><td></td><td></td><td></td><td></td><td></td><td>fig|1280.19811.peg.1920</td><td>LGY17_09950</td><td>0.6688</td><td>0.0286</td><td>fig|1280.19811.peg.1123</td><td>LGY17_05800</td><td>0.7123</td><td>0.0070</td><td>fig|1280.19811.peg.1930</td><td>LGY17_10005</td><td>1.2300</td><td>0.0019</td></tr>
<tr><td>51</td><td></td><td></td><td></td><td></td><td></td><td></td><td></td><td></td><td>fig|1280.19811.peg.1562</td><td>LGY17_08070</td><td>0.6785</td><td>0.0056</td><td>fig|1280.19811.peg.397</td><td>LGY17_02100</td><td>0.7207</td><td>0.0449</td><td>fig|1280.19811.peg.1931</td><td>LGY17_10010</td><td>1.3202</td><td>0.0263</td></tr>
<tr><td>52</td><td></td><td></td><td></td><td></td><td></td><td></td><td></td><td></td><td>fig|1280.19811.peg.1382</td><td>LGY17_07135</td><td>0.6831</td><td>0.0257</td><td>fig|1280.19811.peg.217</td><td>LGY17_01170</td><td>0.7261</td><td>0.0473</td><td>fig|1280.19811.peg.1944</td><td>LGY17_10080</td><td>1.3243</td><td>0.0144</td></tr>
<tr><td>53</td><td></td><td></td><td></td><td></td><td></td><td></td><td></td><td></td><td>fig|1280.19811.peg.1860</td><td>LGY17_09650</td><td>0.7138</td><td>0.0137</td><td>fig|1280.19811.peg.1592</td><td>LGY17_08255</td><td>0.7338</td><td>0.0003</td><td>fig|1280.19811.peg.1748</td><td>LGY17_09070</td><td>1.3311</td><td>0.0109</td></tr>
<tr><td>54</td><td></td><td></td><td></td><td></td><td></td><td></td><td></td><td></td><td>fig|1280.19811.peg.2230</td><td>LGY17_11840</td><td>0.7211</td><td>0.0428</td><td>fig|1280.19811.peg.208</td><td>LGY17_01125</td><td>0.7434</td><td>0.0157</td><td>fig|1280.19811.peg.600</td><td>LGY17_03140</td><td>1.3326</td><td>0.0044</td></tr>
<tr><td>55</td><td></td><td></td><td></td><td></td><td></td><td></td><td></td><td></td><td>fig|1280.19811.peg.1123</td><td>LGY17_05800</td><td>0.7219</td><td>0.0054</td><td>fig|1280.19811.peg.717</td><td>LGY17_03725</td><td>0.7758</td><td>0.0056</td><td>fig|1280.19811.peg.1557</td><td>LGY17_08045</td><td>1.3676</td><td>0.0329</td></tr>
<tr><td>56</td><td></td><td></td><td></td><td></td><td></td><td></td><td></td><td></td><td>fig|1280.19811.peg.1426</td><td>LGY17_07355</td><td>0.7795</td><td>0.0385</td><td>fig|1280.19811.peg.2354</td><td>LGY17_12540</td><td>0.7794</td><td>0.0161</td><td>fig|1280.19811.peg.1258</td><td>LGY17_06500</td><td>1.3980</td><td>0.0163</td></tr>
<tr><td>57</td><td></td><td></td><td></td><td></td><td></td><td></td><td></td><td></td><td>fig|1280.19811.peg.1898</td><td>LGY17_09835</td><td>0.7905</td><td>0.0190</td><td>fig|1280.19811.peg.2147</td><td>LGY17_11365</td><td>0.7840</td><td>0.0259</td><td>fig|1280.19811.peg.535</td><td>LGY17_02815</td><td>1.4068</td><td>0.0021</td></tr>
<tr><td>58</td><td></td><td></td><td></td><td></td><td></td><td></td><td></td><td></td><td>fig|1280.19811.peg.2384</td><td>LGY17_12690</td><td>0.8338</td><td>0.0128</td><td>fig|1280.19811.peg.996</td><td>LGY17_05145</td><td>0.7996</td><td>0.0007</td><td>fig|1280.19811.peg.494</td><td>LGY17_02600</td><td>1.4189</td><td>0.0002</td></tr>
<tr><td>59</td><td></td><td></td><td></td><td></td><td></td><td></td><td></td><td></td><td>fig|1280.19811.peg.1466</td><td>LGY17_07585</td><td>0.8679</td><td>0.0213</td><td>fig|1280.19811.peg.943</td><td>LGY17_04875</td><td>0.8222</td><td>0.0473</td><td>fig|1280.19811.peg.1151</td><td>LGY17_05945</td><td>1.4389</td><td>0.0146</td></tr>
<tr><td>60</td><td></td><td></td><td></td><td></td><td></td><td></td><td></td><td></td><td>fig|1280.19811.peg.1309</td><td>LGY17_06770</td><td>0.8712</td><td>0.0143</td><td>fig|1280.19811.peg.1860</td><td>LGY17_09650</td><td>0.8402</td><td>0.0050</td><td>fig|1280.19811.peg.1749</td><td>LGY17_09075</td><td>1.4701</td><td>0.0020</td></tr>
<tr><td>61</td><td></td><td></td><td></td><td></td><td></td><td></td><td></td><td></td><td>fig|1280.19811.peg.2394</td><td>LGY17_12740</td><td>0.8752</td><td>0.0376</td><td>fig|1280.19811.peg.1889</td><td>LGY17_09790</td><td>0.8460</td><td>0.0442</td><td>fig|1280.19811.peg.1721</td><td>LGY17_08925</td><td>1.4776</td><td>0.0199</td></tr>
<tr><td>62</td><td></td><td></td><td></td><td></td><td></td><td></td><td></td><td></td><td>fig|1280.19811.peg.1890</td><td>LGY17_09795</td><td>0.9125</td><td>0.0166</td><td>fig|1280.19811.peg.1920</td><td>LGY17_09950</td><td>0.8872</td><td>0.0025</td><td>fig|1280.19811.peg.1747</td><td>LGY17_09065</td><td>1.4815</td><td>0.0091</td></tr>
<tr><td>63</td><td></td><td></td><td></td><td></td><td></td><td></td><td></td><td></td><td>fig|1280.19811.peg.786</td><td>LGY17_04065</td><td>0.9140</td><td>0.0224</td><td>fig|1280.19811.peg.498</td><td>LGY17_02625</td><td>0.8959</td><td>0.0288</td><td>fig|1280.19811.peg.235</td><td>LGY17_01260</td><td>1.5041</td><td>0.0022</td></tr>
<tr><td>64</td><td></td><td></td><td></td><td></td><td></td><td></td><td></td><td></td><td>fig|1280.19811.peg.941</td><td>LGY17_04865</td><td>0.9145</td><td>0.0310</td><td>fig|1280.19811.peg.519</td><td>LGY17_02730</td><td>0.9021</td><td>0.0219</td><td>fig|1280.19811.peg.1336</td><td>LGY17_06905</td><td>1.5083</td><td>0.0222</td></tr>
<tr><td>65</td><td></td><td></td><td></td><td></td><td></td><td></td><td></td><td></td><td>fig|1280.19811.peg.1897</td><td>LGY17_09830</td><td>0.9544</td><td>0.0264</td><td>fig|1280.19811.peg.1898</td><td>LGY17_09835</td><td>0.9050</td><td>0.0065</td><td>fig|1280.19811.peg.1556</td><td>LGY17_08040</td><td>1.5421</td><td>0.0028</td></tr>
<tr><td>66</td><td></td><td></td><td></td><td></td><td></td><td></td><td></td><td></td><td>fig|1280.19811.peg.788</td><td>LGY17_04080</td><td>0.9800</td><td>0.0116</td><td>fig|1280.19811.peg.786</td><td>LGY17_04065</td><td>0.9134</td><td>0.0402</td><td>fig|1280.19811.peg.2185</td><td>LGY17_11565</td><td>1.5631</td><td>0.0001</td></tr>
<tr><td>67</td><td></td><td></td><td></td><td></td><td></td><td></td><td></td><td></td><td>fig|1280.19811.peg.864</td><td>LGY17_04470</td><td>0.9937</td><td>0.0022</td><td>fig|1280.19811.peg.1553</td><td>LGY17_08020</td><td>0.9265</td><td>0.0399</td><td>fig|1280.19811.peg.594</td><td>LGY17_03110</td><td>1.6226</td><td>0.0374</td></tr>
<tr><td>68</td><td></td><td></td><td></td><td></td><td></td><td></td><td></td><td></td><td>fig|1280.19811.peg.533</td><td>LGY17_02805</td><td>0.9967</td><td>0.0227</td><td>fig|1280.19811.peg.311</td><td>LGY17_01675</td><td>0.9561</td><td>0.0211</td><td>fig|1280.19811.peg.1289</td><td>LGY17_06665</td><td>1.6529</td><td>0.0001</td></tr>
<tr><td>69</td><td></td><td></td><td></td><td></td><td></td><td></td><td></td><td></td><td>fig|1280.19811.peg.341</td><td>LGY17_01815</td><td>1.0015</td><td>0.0072</td><td>fig|1280.19811.peg.158</td><td>LGY17_00870</td><td>0.9648</td><td>0.0050</td><td>fig|1280.19811.peg.1432</td><td>LGY17_07385</td><td>1.7223</td><td>0.0037</td></tr>
<tr><td>70</td><td></td><td></td><td></td><td></td><td></td><td></td><td></td><td></td><td>fig|1280.19811.peg.2192</td><td>LGY17_11600</td><td>1.0162</td><td>0.0477</td><td>fig|1280.19811.peg.232</td><td>LGY17_01245</td><td>0.9699</td><td>0.0316</td><td>fig|1280.19811.peg.1272</td><td>LGY17_06575</td><td>1.7997</td><td>0.0397</td></tr>
<tr><td>71</td><td></td><td></td><td></td><td></td><td></td><td></td><td></td><td></td><td>fig|1280.19811.peg.311</td><td>LGY17_01675</td><td>1.0231</td><td>0.0050</td><td>fig|1280.19811.peg.788</td><td>LGY17_04080</td><td>0.9726</td><td>0.0173</td><td>fig|1280.19811.peg.663</td><td>LGY17_03450</td><td>1.8154</td><td>0.0270</td></tr>
<tr><td>72</td><td></td><td></td><td></td><td></td><td></td><td></td><td></td><td></td><td>fig|1280.19811.peg.116</td><td>LGY17_00655</td><td>1.0466</td><td>0.0016</td><td>fig|1280.19811.peg.18</td><td>LGY17_00095</td><td>0.9834</td><td>0.0402</td><td>fig|1280.19811.peg.500</td><td>LGY17_02635</td><td>1.8553</td><td>0.0107</td></tr>
<tr><td>73</td><td></td><td></td><td></td><td></td><td></td><td></td><td></td><td></td><td>fig|1280.19811.peg.309</td><td>LGY17_01665</td><td>1.0501</td><td>0.0373</td><td>fig|1280.19811.peg.1458</td><td>LGY17_07545</td><td>0.9844</td><td>0.0368</td><td>fig|1280.19811.peg.595</td><td>LGY17_03115</td><td>1.8597</td><td>0.0008</td></tr>
<tr><td>74</td><td></td><td></td><td></td><td></td><td></td><td></td><td></td><td></td><td>fig|1280.19811.peg.2163</td><td>LGY17_11450</td><td>1.0514</td><td>0.0047</td><td>fig|1280.19811.peg.1309</td><td>LGY17_06770</td><td>0.9953</td><td>0.0085</td><td>fig|1280.19811.peg.1246</td><td>LGY17_06440</td><td>1.8636</td><td>0.0000</td></tr>
</table>

TABLE S4B continued

	Time 2 min				Time 5 min				Time 10 min				Time 20 min				Time 30 min			
#	Gene ID	Locus Tag	log2 Fold Change	p adj. value	Gene ID	Locus Tag	log2 Fold Change	p adj. value	Gene ID	Locus Tag	log2 Fold Change	p adj. value	Gene ID	Locus Tag	log2 Fold Change	p adj. value	Gene ID	Locus Tag	log2 Fold Change	p adj. value
75									fig|1280.19811.peg.1179	LGY17_06110	1.0601	0.0063	fig|1280.19811.peg.2119	LGY17_11215	1.0000	0.0225	fig|1280.19811.peg.787	LGY17_04070	1.8843	0.0068
76									fig|1280.19811.peg.2385	LGY17_12695	1.0603	0.0000	fig|1280.19811.peg.2386	LGY17_12700	1.0125	0.0064	fig|1280.19811.peg.2186	LGY17_11570	1.8914	0.0027
77									fig|1280.19811.peg.1736	LGY17_09000	1.0673	0.0421	fig|1280.19811.peg.2385	LGY17_12695	1.0238	0.0000	fig|1280.19811.peg.790	LGY17_04090	1.9678	0.0029
78									fig|1280.19811.peg.863	LGY17_04465	1.0750	0.0157	fig|1280.19811.peg.1098	LGY17_05670	1.0358	0.0400	fig|1280.19811.peg.2070	LGY17_10750	2.0048	0.0166
79									fig|1280.19811.peg.867	LGY17_04490	1.0820	0.0226	fig|1280.19811.peg.1674	LGY17_08680	1.0801	0.0278	fig|1280.19811.peg.1591	LGY17_08250	2.0455	0.0000
80									fig|1280.19811.peg.119	LGY17_00670	1.1009	0.0345	fig|1280.19811.peg.1930	LGY17_10005	1.0988	0.0060	fig|1280.19811.peg.1481	LGY17_07660	2.0622	0.0109
81									fig|1280.19811.peg.2119	LGY17_11215	1.1035	0.0078	fig|1280.19811.peg.1450	LGY17_07500	1.1125	0.0281	fig|1280.19811.peg.2068	LGY17_10740	2.0648	0.0420
82									fig|1280.19811.peg.236	LGY17_01265	1.1317	0.0069	fig|1280.19811.peg.1148	LGY17_05925	1.1270	0.0048	fig|1280.19811.peg.501	LGY17_02640	2.0807	0.0088
83									fig|1280.19811.peg.1556	LGY17_08040	1.1379	0.0213	fig|1280.19811.peg.1584	LGY17_08210	1.1500	0.0287	fig|1280.19811.peg.1773	LGY17_09205	2.1210	0.0019
84									fig|1280.19811.peg.2127	LGY17_11255	1.1422	0.0345	fig|1280.19811.peg.2127	LGY17_11255	1.1503	0.0356	fig|1280.19811.peg.1390	LGY17_07175	2.1873	0.0000
85									fig|1280.19811.peg.1944	LGY17_10080	1.1639	0.0374	fig|1280.19811.peg.225	LGY17_01210	1.1683	0.0007	fig|1280.19811.peg.1772	LGY17_09200	2.2100	0.0000
86									fig|1280.19811.peg.1258	LGY17_06500	1.1892	0.0295	fig|1280.19811.peg.1425	LGY17_07350	1.1779	0.0041	fig|1280.19811.peg.502	LGY17_02645	2.2692	0.0025
87									fig|1280.19811.peg.2188	LGY17_11580	1.1900	0.0100	fig|1280.19811.peg.296	LGY17_01595	1.1868	0.0314	fig|1280.19811.peg.1483	LGY17_07670	2.2711	0.0000
88									fig|1280.19811.peg.296	LGY17_01595	1.2107	0.0209	fig|1280.19811.peg.1890	LGY17_09795	1.1992	0.0007	fig|1280.19811.peg.2345	LGY17_12495	2.2757	0.0436
89									fig|1280.19811.peg.121	LGY17_00685	1.2391	0.0276	fig|1280.19811.peg.61	LGY17_00380	1.2238	0.0494	fig|1280.19811.peg.1651	LGY17_08560	2.3394	0.0004
90									fig|1280.19811.peg.344	LGY17_01830	1.2425	0.0225	fig|1280.19811.peg.1944	LGY17_10080	1.2363	0.0253	fig|1280.19811.peg.1311	LGY17_06780	2.3722	0.0052
91									fig|1280.19811.peg.1390	LGY17_07175	1.2435	0.0235	fig|1280.19811.peg.1748	LGY17_09070	1.2488	0.0173	fig|1280.19811.peg.236	LGY17_01265	2.3839	0.0000
92									fig|1280.19811.peg.1747	LGY17_09065	1.3091	0.0142	fig|1280.19811.peg.591	NA	1.2592	0.0141	fig|1280.19811.peg.2346	LGY17_12500	2.3913	0.0015
93									fig|1280.19811.peg.1858	NA	1.3101	0.0046	fig|1280.19811.peg.1630	LGY17_08445	1.3018	0.0425	fig|1280.19811.peg.895	NA	2.4254	0.0001
94									fig|1280.19811.peg.1557	LGY17_08045	1.3739	0.0125	fig|1280.19811.peg.494	LGY17_02600	1.3361	0.0006	fig|1280.19811.peg.1775	LGY17_09215	2.4353	0.0012
95									fig|1280.19811.peg.1558	LGY17_08050	1.4780	0.0022	fig|1280.19811.peg.600	LGY17_03140	1.3416	0.0031	fig|1280.19811.peg.606	LGY17_03170	2.4769	0.0000
96									fig|1280.19811.peg.1420	LGY17_07325	1.4798	0.0006	fig|1280.19811.peg.1741	LGY17_09025	1.3425	0.0140	fig|1280.19811.peg.2187	LGY17_11575	2.5706	0.0007
97									fig|1280.19811.peg.1630	LGY17_08445	1.4908	0.0107	fig|1280.19811.peg.1258	LGY17_06500	1.3806	0.0140	fig|1280.19811.peg.590	LGY17_03090	2.6548	0.0000
98									fig|1280.19811.peg.1680	LGY17_08710	1.5680	0.0200	fig|1280.19811.peg.2188	LGY17_11580	1.3884	0.0026	fig|1280.19811.peg.463	LGY17_02420	2.8419	0.0441
99									fig|1280.19811.peg.1336	LGY17_06905	1.5728	0.0140	fig|1280.19811.peg.344	LGY17_01830	1.3970	0.0091	fig|1280.19811.peg.598	LGY17_03130	3.2076	0.0000
100									fig|1280.19811.peg.332	LGY17_01775	1.5796	0.0382	fig|1280.19811.peg.1420	LGY17_07325	1.4097	0.0036	fig|1280.19811.peg.1391	LGY17_07180	3.3345	0.0000
101									fig|1280.19811.peg.1656	LGY17_08585	1.5797	0.0257	fig|1280.19811.peg.653	LGY17_03400	1.4211	0.0080	fig|1280.19811.peg.837	LGY17_04335	3.5170	0.0000
102									fig|1280.19811.peg.504	LGY17_02655	1.8625	0.0457	fig|1280.19811.peg.1746	LGY17_09060	1.4363	0.0397	fig|1280.19811.peg.1310	LGY17_06775	3.5959	0.0003
103									fig|1280.19811.peg.2407	LGY17_12800	1.8991	0.0212	fig|1280.19811.peg.1426	LGY17_07355	1.4683	0.0000	fig|1280.19811.peg.836	LGY17_04330	3.9802	0.0003
104									fig|1280.19811.peg.605	LGY17_03165	1.9149	0.0061	fig|1280.19811.peg.1556	LGY17_08040	1.4789	0.0033	fig|1280.19811.peg.605	LGY17_03165	4.3619	0.0000
105									fig|1280.19811.peg.503	LGY17_02650	2.0488	0.0075	fig|1280.19811.peg.235	LGY17_01260	1.4921	0.0019	fig|1280.19811.peg.604	LGY17_03160	4.3703	0.0000
106									fig|1280.19811.peg.787	LGY17_04070	2.0672	0.0014	fig|1280.19811.peg.1721	LGY17_08925	1.5018	0.0156				
107									fig|1280.19811.peg.1481	LGY17_07660	2.1078	0.0029	fig|1280.19811.peg.1492	LGY17_07720	1.5131	0.0473				
108									fig|1280.19811.peg.1483	LGY17_07670	2.1619	0.0000	fig|1280.19811.peg.1289	LGY17_06665	1.5172	0.0006				
109									fig|1280.19811.peg.1391	LGY17_07180	2.2262	0.0004	fig|1280.19811.peg.1749	LGY17_09075	1.5202	0.0009				
110									fig|1280.19811.peg.837	LGY17_04335	2.3117	0.0148	fig|1280.19811.peg.1147	LGY17_05920	1.5495	0.0145				
111									fig|1280.19811.peg.1310	LGY17_06775	2.3194	0.0477	fig|1280.19811.peg.606	LGY17_03170	1.5610	0.0017				
112									fig|1280.19811.peg.500	LGY17_02635	2.6280	0.0000	fig|1280.19811.peg.1747	LGY17_09065	1.5702	0.0032				
113									fig|1280.19811.peg.501	LGY17_02640	2.7679	0.0001	fig|1280.19811.peg.1336	LGY17_06905	1.5815	0.0140				
114									fig|1280.19811.peg.502	LGY17_02645	2.9452	0.0000	fig|1280.19811.peg.1603	LGY17_08315	1.5923	0.0053				
115													fig|1280.19811.peg.1611	LGY17_08350	1.6047	0.0056				
116													fig|1280.19811.peg.1656	LGY17_08585	1.6623	0.0174				
117													fig|1280.19811.peg.1772	LGY17_09200	1.6835	0.0004				
118													fig|1280.19811.peg.1661	LGY17_08610	1.7158	0.0220				
119													fig|1280.19811.peg.1393	LGY17_07190	1.7629	0.0389				
120													fig|1280.19811.peg.1246	LGY17_06440	1.7861	0.0000				
121													fig|1280.19811.peg.1651	LGY17_08560	1.7945	0.0131				
122													fig|1280.19811.peg.2407	LGY17_12800	1.8162	0.0316				
123													fig|1280.19811.peg.1151	LGY17_05945	1.8956	0.0004				
124													fig|1280.19811.peg.2186	LGY17_11570	1.8978	0.0017				
125													fig|1280.19811.peg.503	LGY17_02650	1.9166	0.0185				
126													fig|1280.19811.peg.1591	LGY17_08250	1.9315	0.0001				
127													fig|1280.19811.peg.787	LGY17_04070	1.9403	0.0041				
128													fig|1280.19811.peg.1272	LGY17_06575	1.9453	0.0193				
129													fig|1280.19811.peg.595	LGY17_03115	1.9745	0.0002				
130													fig|1280.19811.peg.895	NA	2.0038	0.0032				
131													fig|1280.19811.peg.1773	LGY17_09205	2.1057	0.0013				
132													fig|1280.19811.peg.590	LGY17_03090	2.1386	0.0000				
133													fig|1280.19811.peg.1481	LGY17_07660	2.1589	0.0044				
134													fig|1280.19811.peg.500	LGY17_02635	2.1940	0.0011				
135													fig|1280.19811.peg.1390	LGY17_07175	2.1996	0.0000				
136													fig|1280.19811.peg.236	LGY17_01265	2.2325	0.0000				
137													fig|1280.19811.peg.1775	LGY17_09215	2.2530	0.0033				
138													fig|1280.19811.peg.1483	LGY17_07670	2.2946	0.0000				
139													fig|1280.19811.peg.501	LGY17_02640	2.3547	0.0017				
140													fig|1280.19811.peg.2187	LGY17_11575	2.4921	0.0008				
141													fig|1280.19811.peg.598	LGY17_03130	2.5431	0.0000				
142													fig|1280.19811.peg.1373	LGY17_07095	2.5785	0.0327				
143													fig|1280.19811.peg.502	LGY17_02645	2.6830	0.0001				
144													fig|1280.19811.peg.2392	LGY17_12730	3.0419	0.0241				
145													fig|1280.19811.peg.1391	LGY17_07180	3.2278	0.0000				
146													fig|1280.19811.peg.604	LGY17_03160	3.2953	0.0009				
147													fig|1280.19811.peg.836	LGY17_04330	3.3011	0.0053				
148													fig|1280.19811.peg.1310	LGY17_06775	3.3786	0.0010				
149													fig|1280.19811.peg.837	LGY17_04335	3.5994	0.0000				
150													fig|1280.19811.peg.605	LGY17_03165	3.6567	0.0000				
